# The synthesis of chiral β-naphthyl-β-sulfanyl ketones via enantioselective sulfa-Michael reaction in the presence of a bifunctional cinchona/sulfonamide organocatalyst

**DOI:** 10.3762/bjoc.17.43

**Published:** 2021-02-18

**Authors:** Deniz Tözendemir, Cihangir Tanyeli

**Affiliations:** 1 Department of Chemistry, Middle East Technical University, 06800 Ankara, Turkey

**Keywords:** asymmetric synthesis, bifunctional catalysis, cinchona alkaloids, organocatalysis, sulfa-Michael reaction

## Abstract

Cinchona alkaloid-derived organocatalysts are widely employed in various asymmetric transformations, yielding products with high enantiopurity. In this respect, a bifunctional quinine-derived sulfonamide organocatalyst was developed to catalyze the asymmetric sulfa-Michael reaction of naphthalene-1-thiol with *trans*-chalcone derivatives. The target sulfa-Michael adducts were obtained with up to 96% ee under mild conditions and with a low (1 mol %) catalyst loading. Selected enantiomerically enriched sulfa-Michael addition products were subjected to oxidation to obtain the corresponding sulfones.

## Introduction

Derivatives of the naturally occurring cinchona alkaloids have shown remarkable performance as organocatalysts for stereoselective synthesis in the past decade [1–6]. Among them, quinine-derived organocatalysts make a noteworthy appearance in the formation of new stereogenic centres, which can serve as valuable building blocks for the construction of more elaborate structures [7–11]. An outstanding class of quinine derived organocatalysts exhibits a bifunctional mode of activation by the incorporation of an acidic unit, such as urea, thiourea, squaramide or sulfonamide moieties, giving rise to the simultaneous activation of both the nucleophile and the electrophile [12–15]. Quinine derived sulfonamides were first introduced to the literature by Song et al. [16]. Since then, many contributions were made regarding their applications in a variety of reaction types [17–20]. However, sulfa-Michael addition (SMA) reactions remain a rather less explored reaction among asymmetric organocatalytic transformations, mainly because of the high nucleophilicity of thiols causing difficulties in controlling the stereoselectivity [21], despite C–S bond-forming reactions are of great interest in synthetic organic chemistry [22]. Thus, the SMA with thiols and α,β-unsaturated ketones are generally carried out at low temperatures and with high catalyst loading [23–26]. The studies that employ mild conditions and low catalyst loading use thiophenol derivatives or simple alkylthiols as nucleophiles [27–30]. Thionaphthols, however, are overlooked in sulfa-Michael addition reactions. And to our best knowledge, no study is present concerned with SMAs with naphthalene-1-thiol as the nucleophile for the addition to enones.

Encouraged by the good results obtained with enantioselective sulfa-Michael additions of thiols to chalcones with sulfonamide-type organocatalysts in the literature [30–31], in this study, a new quinine sulfonamide organocatalyst derivative was developed to catalyze the enantioselective SMA of naphthalene-1-thiol to *trans*-chalcones under mild conditions and with a low (1 mol %) catalyst loading, to obtain enantiomerically enriched β-naphthyl-β-sulfanyl ketones with up to 96% ee. The target adducts are the core structure of *seco*-raloxifene derivatives, which are potent anti-breast cancer agents [32]. In addition, the same scaffold has also shown urease inhibitor activity [33]. Due to the shown biological attractiveness of those 1,3-biarylsulfanyl derivatives, the enantioenriched products can serve as important building blocks for new drugs. The sulfide moiety of β-naphthyl-β-sulfanyl ketones can be oxidized to form sulfones. Despite that sulfones were outshined by sulfonamides in medicinal chemistry, they have a large array of biologic activities that show promising effects as potent anti-HIV-1 [34], anti-hepatitis C [35], antifungal [36], insecticidal/acaricial [37] and antimalarial [38] agents.

## Results and Discussion

**Scheme 1:**
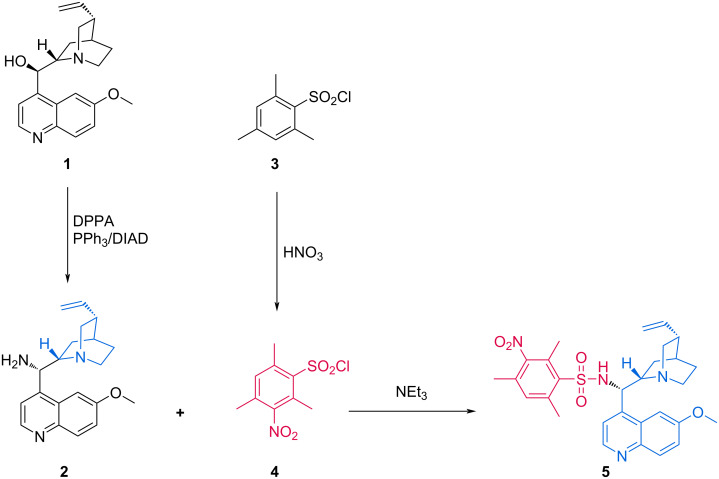
Synthesis of organocatalyst **5**.

We have previously reported the synthesis of new amino-substituted-DMAP-based sulfonamides [39] and quinine-based squaramide-type organocatalysts [40]. Motivated by the excellent results obtained with our aforementioned catalysts, we developed a new chiral bifunctional sulfonamide–quinine organocatalyst that unites both classes. The synthesis of the basic part was initiated by converting quinine to quinineamine via a Mitsunobu reaction, followed by a Staudinger reduction [40]. Then it was coupled with the acidic part, which was obtained by the nitration of 2,4,6-trimethylbenzenesulfonyl chloride [39] to obtain organocatalyst **5** (Scheme 1).

**Figure 1:**
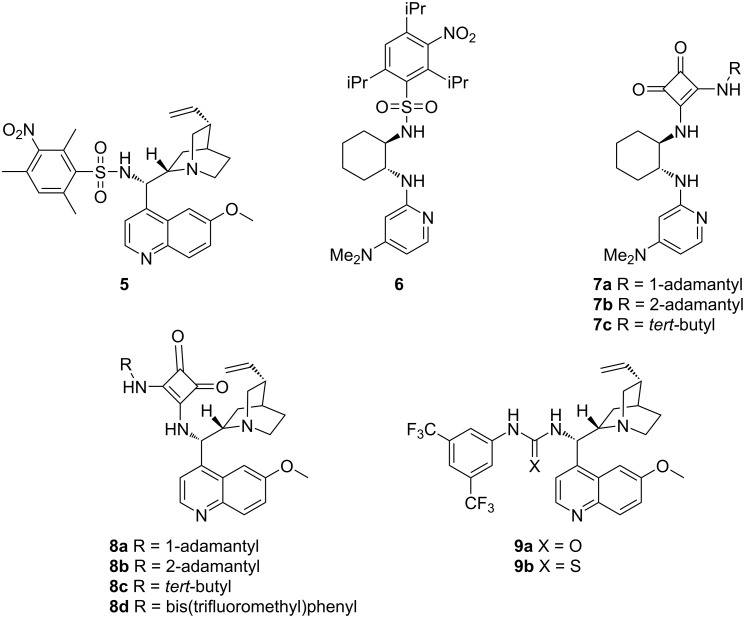
Structures of the screened organocatalysts.

This new organocatalyst was employed in the model asymmetric sulfa-Michael reaction of naphthalene-1-thiol and *trans-*chalcone, in addition to the amino-substituted DMAP and quinine-based organocatalysts (**6**, **7a–c** and **8a–c**) in our library (Figure 1), as well as previously reported quinine derived organocatalysts **8d** and **9a**,**b** in the literature [30,41].

Among the 11 screened organocatalysts, the ones with amino-substituted DMAP cores gave unimpressive ee values (Table 1, entries 2–5). The quinine-derived organocatalysts **8a–d** failed to attain striking stereoselectivity (25–43% ee). The popular urea–quinine and thiourea–quinine organocatalysts both gave the target compound with only 41% ee, which was well below satisfactory (Table 1, entries 10 and 11). The best catalyst in terms of enantioselectivity proved to be the newly designed catalyst **5**, which gave the desired product with 63% ee in 1 hour (Table 1, entry 1). After selecting the best-working catalyst, optimization studies were initiated on the model reaction to determine the conditions to achieve the best enantioselectivity.

**Table 1:** Catalyst and solvent screening.

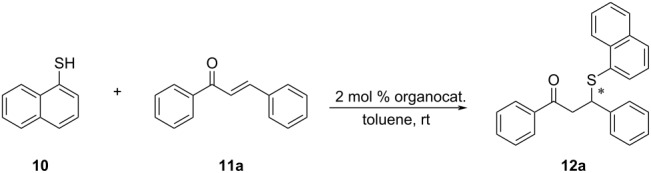					

Entry^a^	Catalyst	Solvent	Time (h)	Yield^b^ (%)	ee^c^ (%)

1	**5**	toluene	1	98	63
2	**6**	toluene	2.5	>99	30
3	**7a**	toluene	0.5	92	46
4	**7b**	toluene	0.5	63	40
5	**7c**	toluene	0.5	79	52
6	**8a**	toluene	0.5	72	41
7	**8b**	toluene	0.5	96	43
8	**8c**	toluene	0.5	90	31
9	**8d**	toluene	0.5	95	25
10	**9a**	toluene	0.5	71	41
11	**9b**	toluene	0.5	94	41
12	**5**	DCM	2.5	42	65
13	**5**	hexane	1	>99	6
14	**5**	THF	4	85	79
15	**5**	CHCl_3_	2	50	65
16	**5**	dioxane	4	90	75
17	**5**	TBME	1	92	57
18	**5**	EtOAc	2	49	65
19	**5**	MeCN	1	81	55
20	**5**	Et_2_O	3	28	57

^a^Unless stated otherwise, all reactions were performed with 0.10 mmol *trans*-chalcone and 0.20 mmol naphthalene-1-thiol in 0.5 mL of solvent, in the presence of 2 mol % organocatalyst at rt. ^b^Isolated yields. ^c^Determined by chiral HPLC analysis, AD-H column, hexane/isopropanol (99:1), 0.8 mL/min, 220 nm.

The first parameter screened was the effect of the solvent. Using THF and dioxane (Table 1, entries 14 and 16, respectively) gave the two highest results, but the use of dioxane is best avoided due to its toxicity. Except for hexane (only 6% ee, Table 1, entry 13), which resulted in an almost racemic product presumably due to solubility issues, all other solvents afforded the target SMA adduct with similar moderate ee values. Thus, THF was selected as the best solvent despite the longer reaction duration.

Then, the catalyst loading was varied between 0.1 and 10 mol % to investigate its effect on the enantioselectivity (Table 2, entries 1–6). At an extremely low catalyst loading of 0.1 mol %, the reaction was too sluggish; the amount of the product was too small and it was not isolated. Using 0.5 mol % of **5** gave rise to 82% ee (Table 2, entry 2), however, the outcome of the reaction with 1 mol % of **5** (83%, Table 2, entry 3) was slightly better than the former and was completed in a shorter time (40 hours, compared to 23 hours, respectively). Thus, that part of the optimization was continued with 1 mol % catalyst loading.

**Table 2:** Further screening results of the model reaction.

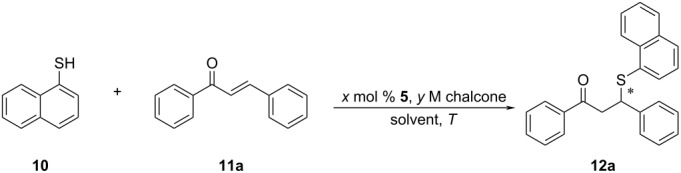						

Entry^a^	Catalyst loading (mol %)	Conc. (M)	*T* (°C)	Time (h)	Yield^b^ (%)	ee^c^ (%)

1	0.1	0.2	rt	72	–	–
2	0.5	0.2	rt	40	81	82
3	1	0.2	rt	23	93	83
4	2	0.2	rt	4	85	79
5	5	0.2	rt	6	95	76
6	10	0.2	rt	3	83	69
7	1	0.05	rt	72	–	–
8	1	0.1	rt	72	–	–
9	1	0.15	rt	26	>99	79
10	1	0.25	rt	25	>99	78
11	1	0.3	rt	20	91	79
12	1	0.4	rt	19	>99	75
13^d^	1	0.2	rt	72	–	–
14^e^	1	0.2	rt	41	91	79
15^f^	1	0.2	rt	19	90	78
16	1	0.2	0	40	96	73
17	1	0.2	−20	41	97	68
18	1	0.2	−40	49	90	62

^a^Unless stated otherwise, all reactions were performed with a 1:2 ratio of *trans*-chalcone/naphthalene-1-thiol in THF, in the presence of organocatalyst **5** at the indicated temperature. ^b^Isolated yields. ^c^Determined by chiral HPLC analysis, AD-H column, 99:1 hexane/isopropanol, 0.8 mL/min, 220 nm. ^d^The reaction was carried out using a 1:1 ratio of *trans*-chalcone/naphthalene-1-thiol. ^e^The reaction was carried out using a 1:1.5 ratio of *trans*-chalcone/naphthalene-1-thiol. ^f^The reaction was carried out using a 1:3 ratio of *trans*-chalcone/naphthalene-1-thiol.

The effect of the concentration of the reaction mixture was investigated by changing the chalcone concentration gradually from 0.05 to 0.4 M (Table 2, entries 3 and 7–12). The best selectivity (83% ee) was obtained at a 0.2 M chalcone concentration (Table 2, entry 3). Diluting the reaction mixture further than 0.15 M had decreased the rate of reaction considerably and the amounts of the products were not sufficient to be isolated. Increasing the concentration led to a small decrease in ee. Using an equimolar mixture of chalcone and naphthalene-1-thiol had a similar outcome on the progress of the reaction as dilution (Table 2, entry 14). Changing the chalcone/naphthalene-1-thiol ratios to 1:1.5 or 1:3 resulted in small losses in enantioselectivity. Hence, the studies were continued with 2 molar equivalents of naphthalene-1-thiol to *trans*-chalcone.

The optimization studies were concluded by investigating the effect of the temperature on the asymmetric induction (Table 2, entries 16–18). Lowering the temperature gradually to −40 °C caused a significant loss in ee, allowing the synthesis of the product **12a** with a final ee of 62% (Table 2, entry 18). This unexpected phenomenon could be linked to an enthalpic factor that favors the formation of the major enantiomer at higher temperature, or due to a change in the reaction mechanism when the temperature was altered. The most enantioenriched product was ultimately obtained at room temperature (83% ee, Table 2, entry 3).

**Scheme 2:**
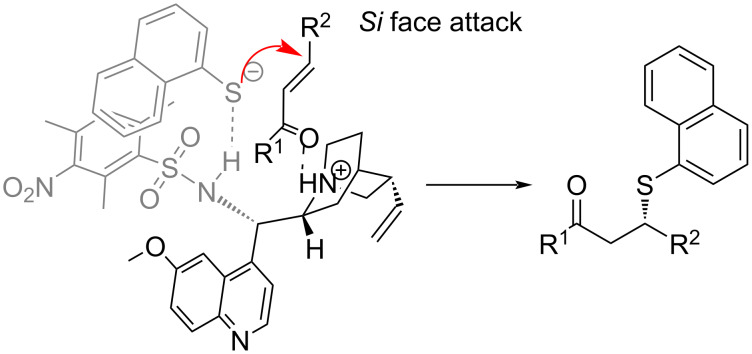
Proposed transition state for the SMA of 1-thionaphthol to *trans*-chalcones.

The absolute configuration of the product was assigned as “*S*” by comparing the obtained optical rotation value with the values in the literature for the organocatalytic SMA of thiols to *trans*-chalcone derivatives [29,42]. A transition state model to explain the origin of the stereoinduction was proposed (Scheme 2), according to the Houk’s Brønsted acid hydrogen bonding model. Guo’s computational work in 2017 on the sulfa-Michael addition of thiols to enones in the presence of cinchona alkaloid-type organocatalysts showed that Houk’s mode of activation was of lower energy than Wynberg’s activation mode, in which the activation and orientation of the nucleophile is done by the quinuclidine core [43]. According to our proposed model, the protonated quinuclidinium ion stabilizes the newly forming alkoxide on the electrophile while the deprotonated nucleophile is oriented by the Brønsted acid moiety.

The substrate scope was extended to substituted chalcones, under the optimized conditions (Table 3). The chalcone derivatives used in this work were obtained by Claisen–Schmidt condensation, using known procedures [44]. Among the chalcone derivatives employed in the model reaction, the best result in terms of enantioselectivity was attained with 4-methyl-substituted chalcone, which allowed the synthesis of the corresponding SMA adduct **12d** with an excellent ee of 91% (Table 3, entry 4). Good to moderate results were obtained with chalcone derivatives possessing either electron-donating or -withdrawing substituents. Compared to the unsubstituted *trans*-chalcone, an unexpected and drastic decrease in enantioselectivity was observed with chalcone derivative **11c**, however (23% ee, Table 3, entry 3).

**Table 3:** Results of the SMA of naphthalene-1-thiol to substituted *trans-*chalcones in THF.

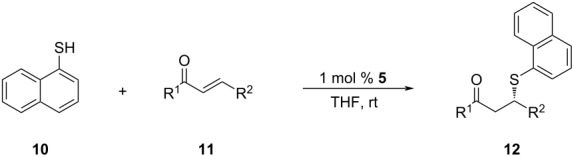						

Entry^a^	**11**	R^1^, R^2^	**12**	Time (h)	Yield^b^ (%)	ee^c^ (%)

1	**11a**	Ph, Ph	**12a**	23	93	83
2	**11b**	Ph, 3-MeC_6_H_4_	**12b**	46	87	78
3	**11c**	Ph, 4-MeC_6_H_4_	**12c**	20	>99	23
4	**11d**	4-MeC_6_H_4_, Ph	**12d**	42	>99	91
5	**11e**	Ph, 3-OMeC_6_H_4_	**12e**	42	83	58
6	**11f**	Ph, 4-OMeC_6_H_4_	**12f**	20	88	63
7	**11g**	Ph, 3,4,5-(OMe)_3_C_6_H_2_	**12g**	21	94	50
8	**11h**	Ph, 2-ClC_6_H_4_	**12h**	23	84	66
9	**11i**	Ph, 3-ClC_6_H_4_	**12i**	22	79	85
10	**11j**	Ph, 4-ClC_6_H_4_	**12j**	21	>99	71
11	**11k**	Ph, 3-BrC_6_H_4_	**12k**	40	66	51
12	**11l**	4-BrC_6_H_4_, Ph	**12l**	23	>99	82
13	**11m**	Ph, 4-CF_3_C_6_H_4_	**12m**	19	79	67
14	**11n**	2-NO_2_C_6_H_4_, Ph	**12n**	21	84	82
15	**11o**	Ph, 4-NO_2_C_6_H_4_	**12o**	21	81	68

^a^Unless stated otherwise, all reactions were performed with 0.20 mmol *trans-*chalcone and 0.40 mmol naphthalene-1-thiol in 1.0 mL of THF, in the presence of 1 mol % **5** at rt. ^b^Isolated yields. ^c^Determined by chiral HPLC analysis.

Intrigued by this unexpected result, we decided to revisit the solvent screening. For this purpose, the sulfa-Michael addition of naphthalene-1-thiol to **11c** was carried out again in toluene, dioxane, DCM and THF (Table 4).

**Table 4:** Solvent screening results for the SMA of naphthalene-1-thiol to chalcone derivative **11c**.

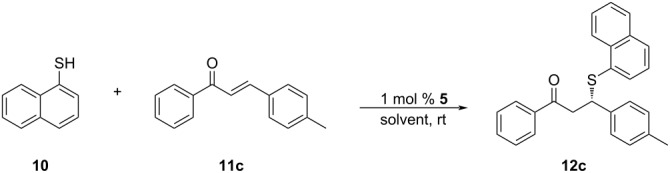				

Entry^a^	Solvent	Time (h)	Yield^b^ (%)	ee^c^ (%)

1	THF	20	>99	23
2	toluene	6	95	78
3	dioxane	20	78	76
4	DCM	6	94	93

^a^Unless stated otherwise, all reactions were performed with 0.20 mmol **11c** and 0.40 mmol naphthalene-1-thiol in 1.0 mL of solvent, in the presence of 1 mol % **5** at rt. ^b^Isolated yields. ^c^Determined by chiral HPLC analysis.

In DCM, 93% ee was attained for adduct **12c**. In the light of this striking result, we decided to repeat the derivatization studies with DCM (Table 5).

**Table 5:** Results for the SMA of naphthalene-1-thiol to substituted *trans*-chalcones in DCM.

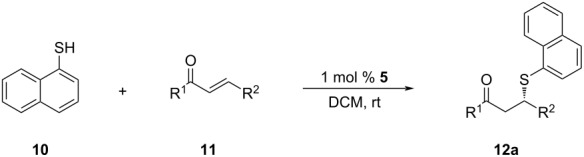						

Entry^a^	**11**	R^1^, R^2^	Product	Time (h)	Yield^b^ (%)	ee^c^ (%)

1	**11a**	Ph, Ph	**12a**	21	90	66
2	**11b**	Ph, 3-MeC_6_H_4_	**12b**	22	77	70
3	**11c**	Ph, 4-MeC_6_H_4_	**12c**	6	94	94
4	**11d**	4-MeC_6_H_4_, Ph	**12d**	21	>99	86
5	**11e**	Ph, 3-OMeC_6_H_4_	**12e**	24	73	63
6	**11f**	Ph, 4-OMeC_6_H_4_	**12f**	23	93	84
7	**11g**	Ph, 3,4,5-(OMe)_3_C_6_H_2_	**12g**	24	>99	96
8	**11h**	Ph, 2-ClC_6_H_4_	**12h**	23	85	44
9	**11i**	Ph, 3-ClC_6_H_4_	**12i**	21	71	15
10	**11j**	Ph, 4-ClC_6_H_4_	**12j**	21	86	51
11	**11k**	Ph, 3-BrC_6_H_4_	**12k**	23	84	26
12	**11l**	4-BrC_6_H_4_, Ph	**12l**	23	57	74
13	**11m**	Ph, 4-CF_3_C_6_H_4_	**12m**	2	88	2
14	**11n**	2-NO_2_C_6_H_4_, Ph	**12n**	21	91	74
15	**11o**	Ph, 4-NO_2_C_6_H_4_	**12o**	23	92	6

^a^Unless stated otherwise, all reactions were performed with 0.20 mmol *trans-*chalcone and 0.40 mmol naphthalene-1-thiol in 1.0 mL of DCM, in the presence of 1 mol % **5** at rt. ^b^Isolated yields. ^c^Determined by chiral HPLC analysis.

Employing DCM as the solvent showed significant improvements in the asymmetric induction for the chalcone derivatives having electron-donating methyl and methoxy substituents (Table 5, entries 3–7), especially with 4-tolyl- and 3,4,5-trimethoxyphenyl substituents. For the 4-tolyl derivative, the 23% ee value obtained with THF (Table 3, entry 3) was increased to 94% when switched to DCM (Table 5, entry 3). For the latter case, a significant improvement in the ee was observed from 50% (Table 3, entry 7) to 96% ee (Table 5, entry 7). The only exception to this pattern was with the 3-methyl derivative of chalcone, which resulted in a small decrease in enantioselectivity (78% to 70% ee, entry 2 in Table 3 and Table 5) when THF was changed to DCM. For halogens and electron-withdrawing substituents, an opposite behavior was observed. The use of DCM instead of THF led to lower ee values for the chalcone derivatives having the aforementioned substituents (Table 5, entries 8–15).

**Figure 2:**
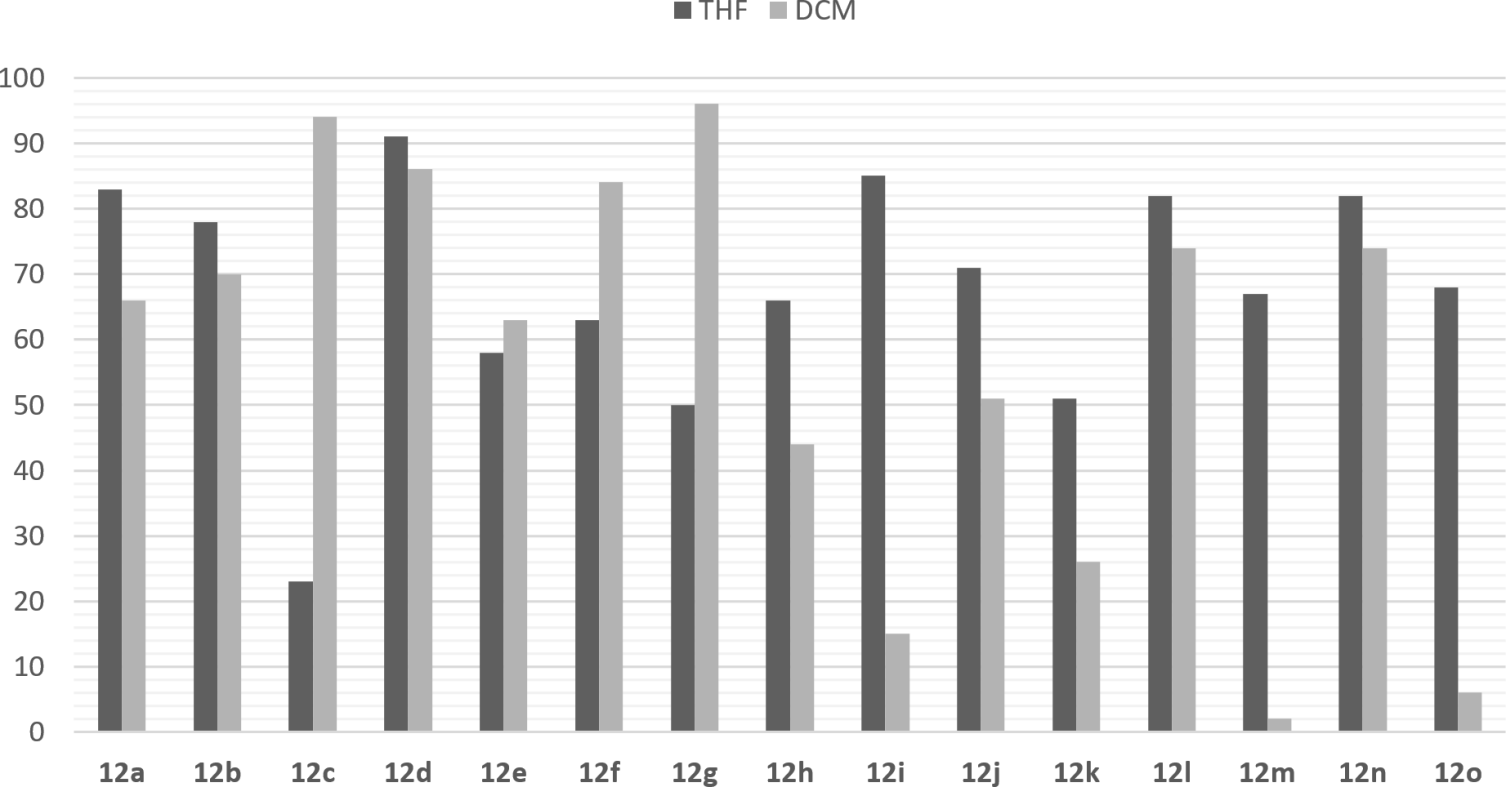
Comparison of the ee values of SMA in the presence of THF and DCM as solvent.

The most dramatic decreases in the selectivity were observed for derivatives **12m** and **12o**, for which the outcomes of the reactions were almost racemic (Table 5, entries 13 and 15). The solvent effects on the SMA of the chalcone derivatives and naphthalene-1-thiol are summarized in Figure 2. This behavior might be related to the better stabilization of the transition state of the substrates containing electron-withdrawing substituents or halogen atoms with THF, or vice versa.

In order to enhance the potential bioactivity of the obtained enantioenriched products, selected SMA adducts (β-naphthyl-β-sulfanyl ketones) were subjected to oxidation with *m*-CPBA (Table 6) [45].

**Table 6:** Synthesis of enantioenriched sulfones from β-naphthyl-β-sulfanyl ketones^a^.

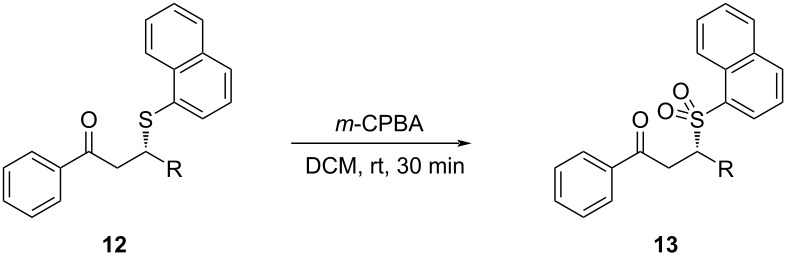				

Entry^a^	R	Product	Yield^b^ (%)	ee^c^ (%)

1	Ph	**13a**	43	86
2	3-OMeC_6_H_4_	**13b**	45	68
3	4-OMeC_6_H_4_	**13c**	62	86
4	4-ClC_6_H_4_	**13d**	28	66

^a^Unless stated otherwise, all reactions were performed with 1 equiv β-aryl-β-sulfanyl ketone derivative and 2.2 equiv *m*-CPBA in DCM.

During the oxidation reaction, it was seen that the enantioenriched sulfa-Michael adducts undergo retro-sulfa-Michael reaction. The low yields of the oxidation and the fluctuations in enantioselectivity compared to the starting sulfa-Michael adducts can be attributed to this unpreventable retro-reaction. Despite this setback, the target sulfones were obtained with moderate to good ee values.

## Conclusion

In conclusion, we report the enantioselective organocatalytic sulfa-Michael addition reaction of naphthalene-1-thiol to *trans*-chalcones, in the presence of a new bifunctional quinine derived sulfonamide organocatalyst. The adducts obtained with moderate to excellent ee values are β-naphthyl-β-sulfanyl ketones, which have potent activity against breast cancer. The easy access to the corresponding sulfones presents a versatile route for the implementation of a new biologically active moiety, the sulfone, to the β-naphthyl-β-sulfanyl ketones. The enantioenriched products of both classes can be evaluated as building blocks of new potential drug molecules.

## Experimental

### Materials and methods

All chemicals were purchased from Sigma-Aldrich or Acros Organics. Column chromatography was performed using silica gel 60 (200–300 mesh) as supporting material. All eluents were distilled prior to use. ^1^H NMR and ^13^C NMR spectra were recorded on a 400 MHz spectrometer, using CDCl_3_ as solvent. Chemical shift values are reported in ppm with TMS as standard, *J* values are given in Hertz. Optical rotations were determined by a polarimeter and reported as [α]DT (*c* in g/100 mL solvent). Enantiomeric excess values were determined by chiral HPLC chromatography using Agilent instrument. All new products were further analyzed by LC/MS–HRMS–TOF or MALDI–ESI–TOFMS.

### Synthesis of organocatalyst **5**

A solution of quinineamine **2** (226.40 mg, 0.70 mmol) and triethylamine (107 μL, 0.77 mmol) in CH_2_Cl_2_ was added to a screw-capped reaction vial. To this mixture, 2,4,6-trimethyl-3-nitrobenzenesulfonyl chloride **4** (184.59 mg, 0.70 mmol) was added at 0 °C. The mixture was then allowed to warm up to room temperature and stirred overnight. The crude product was directly loaded on a silica gel column, with ethyl acetate/triethylamine (98:2) as the eluent to afford the corresponding sulfonamide **5**.

### General procedure for the synthesis of **12a–o**

A screw-capped reaction vial was charged with *trans-*chalcone derivative **11a–o** (0.20 mmol) and organocatalyst **5** (1.10 mg, 0.0020 mmol) and 0.5 mL of THF or DCM. Then a solution of naphthalene-1-thiol (**10**, 55.4 µL, 0.40 mmol) in 1.0 mL of the selected solvent was introduced slowly into the vial with stirring. The mixture was stirred at room temperature and the progress of the reaction was monitored by TLC. Upon the consumption of the chalcone derivative, the reaction mixture was directly subjected to flash column chromatography, using a *n*-hexane/ethyl acetate mixture as eluent to yield the products **12a–o**.

### General procedure for the synthesis of sulfones

A solution of β-naphthyl-β-sulfanyl ketone (0.1 mmol) in 0.8 mL DCM was cooled to 0 °C. *m*-CPBA (0.22 mmol, 37.97 mg) was added to this stirred solution portionwise in 15 minutes. Then, the mixture was allowed to warm up to room temperature and stirred for a total of 30 minutes. After the completion of the reaction, the reaction mixture was diluted with 0.8 mL of DCM. Then washed with 3 × 0.8 mL of 5% K_2_CO_3_ (aq) and 3 × 1 mL of 5% NaHCO_3_ (aq) to remove the excess *m*-CPBA. The aqueous layer was extracted with DCM (3 × 1 mL). The organic layers were then combined, dried over Na_2_SO_4_ and concentrated in vacuo. The crude product was purified by column chromatography on silica with using *n*-hexane/ethyl acetate as eluent to afford the target sulfones.

## Supporting Information

File 1 Copies of ^1^H and ^13^C NMR spectra, HPLC chromatograms and characterization data of the products.
