# Supplementary material for: The synthesis of chiral β-naphthyl-β-sulfanyl ketones via enantioselective sulfa-Michael reaction in the presence of a bifunctional cinchona/sulfonamide organocatalyst
Source: Beilstein J Org Chem. 2021 Feb 18;17:494–503. doi: 10.3762/bjoc.17.43 (PMC7934708; doi:10.3762/bjoc.17.43)
Supplement: File 1 — Copies of 1H and 13C NMR spectra, HPLC chromatograms and characterization data of the products. [file Beilstein_J_Org_Chem-17-494-s001.pdf]

BEILSTEIN JOURNAL OF ORGANIC CHEMISTRY

Supporting Information

for

The synthesis of chiral β-naphthyl-β-sulfanyl ketones via enantioselective sulfa-Michael reaction in the presence of a bifunctional cinchona/sulfonamide organocatalyst

Deniz Tözendemir and Cihangir Tanyeli

Beilstein J. Org. Chem. **2021,** *17,* 494–503. doi:10.3762/bjoc.17.43

Copies of ^{1}H and ^{13}C NMR spectra, HPLC chromatograms and characterization data of the products

License and Terms: This is a supporting information file under the terms of the Creative Commons Attribution License (https://creativecommons.org/licenses/by/4.0). Please note that the reuse, redistribution and reproduction in particular requires that the author(s) and source are credited and that individual graphics may be subject to special legal provisions.
The license is subject to the *Beilstein Journal of Organic Chemistry* terms and conditions: (https://www.beilstein-journals.org/bjoc/terms)

CONTENTS

I. General information S1
II. Characterization of products S1
III. ^{1}H and ^{13}C NMR spectra of catalyst **5** S8
IV. ^{1}H and ^{13}C NMR spectra of products **12a–o** S9
V. HPLC chromatograms of products **12a–o** S24
VI. ^{1}H and ^{13}C NMR spectra of sulfones S39
VII. HPLC chromatograms of sulfones S43

I. General information

All chemicals were purchased from Sigma-Aldrich or Acros Organics. Column chromatography was performed using silica gel 60 (200-300 mesh) as supporting material. All the eluents were distilled prior to use. ^{1}H NMR and ^{13}C NMR spectra were recorded on a 400 MHz spectrometer, using $CDCl_3$ as the solvent. Chemical shift values are reported in ppm with TMS a standard, *J* values are given in hertz. Optical rotations were determined by the use of a polarimeter and reported as $[\alpha]_D^T$ (*c* in g/100 mL, solvent). Enantiomeric excess values were determined by chiral HPLC chromatography using Thermo-Finnigan instrument. All new products were further analyzed by LC/MS–HRMS–TOF or MALDI–ESI–TOFMS.

II. Characterization of products

N-((S)-(6-methoxyquinolin-4-yl)((1S,2S,4S,5R)-5-vinylquinuclidin-2-yl)methyl)-2,4,6-trimethyl-3-nitrobenzenesulfonamide (5)

Off-white solid, 135.3 mg, mp 101 °C, 35% yield. $[\alpha]_D^{30}$= -134.95 (*c* 0.780, MeOH). ^{1}H NMR (400 MHz, $CDCl_3$) (mixture of rotamers) δ 8.58 (d, *J* = 4.2 Hz, 1H), 8.47 (d, *J* = 4.5 Hz, 1H), 7.97 (d, *J* = 9.1 Hz, 1H), 7.87 (d, *J* = 9.2 Hz, 1H), 7.74 – 7.53 (m, 1H), 7.50 – 7.45 (m, 1H), 7.44 – 7.31 (m, 1H), 7.20 (d, *J* = 4.2 Hz, 1H), 7.15 (d, *J* = 4.5 Hz, 1H), 6.54 (s, 1H), 6.44 (s, 1H), 5.78 – 5.56 (m, 1H), 5.31 (s, 1H), 5.10 (d, *J* = 10.6 Hz, 1H), 5.04 – 4.85 (m, 1H), 4.46 (d, *J* = 10.9 Hz, 1H), 3.99 (s, 1H), 3.94 (s, 1H), 3.46 (dd, *J* = 17.9, 9.4 Hz, 1H), 3.38 – 3.25 (m, *J* = 12.0 Hz, 1H), 3.22 – 3.09 (m, *J* = 13.5, 6.8 Hz, 1H), 3.09 – 2.92 (m, 1H), 2.90 – 2.71 (m, 1H), 2.41 (s, 1H), 2.39 (s, 1H), 2.34 (s, 1H), 2.13 (s, 1H), 2.12 (s, 1H), 1.96 (s, 1H), 1.75 (s, 1H), 1.71 – 1.59 (m, 2H), 1.48 – 1.19 (m, *J* = 30.9, 19.8 Hz, 1H), 1.06 – 0.79 (m, *J* = 28.0, 13.4, 7.0 Hz, 1H). ^{13}C NMR (100 MHz, $CDCl_3$) (mixture of rotamers): δ 157.81, 156.81, 151.83, 151.22, 147.04, 146.51, 144.45, 144.18, 141.61, 140.97, 140.91, 140.75, 140.47, 138.89, 135.77, 134.82, 132.60, 132.40, 132.30, 132.13, 131.99, 131.89, 131.82, 131.79, 131.74, 131.33, 130.04, 129.81, 128.42, 128.30, 128.03, 126.34, 123.68, 121.35, 120.17, 119.72, 118.42, 114.72, 103.64, 100.68, 62.88, 61.06, 60.23, 55.76, 55.45, 55.28, 52.78, 40.27, 39.77, 39.42, 39.22, 27.74, 27.51, 27.21, 27.09, 26.06, 24.76, 23.22, 22.50, 16.83, 16.75, 15.88, 15.50, 14.06. IR (neat): 3077, 2934, 2871, 1619, 1589, 1530, 1506, 1475, 1429, 1359, 1262, 1238, 1168, 1138, 1103, 1032, 987, 912, 865, 840, 775, 717, 666, 563, 493 cm^{-1}. HRMS (ESI-TOF) *m/z*: $[M + H]^+$ Calcd. for $C_{29}H_{35}N_4O_5S$ 551.2328; Found 551.2339.

3-(Naphthalen-1-ylthio)-1,3-diphenylpropan-1-one (12a)

White solid, 68.61 mg, mp 99 °C, 93% yield in 23 h, in THF. HPLC (AD-H, 99:1 *n*-Hexane/Isopropanol, 0.8 mL/min, 220 nm): t_{minor} = 33.445 min, t_{major} = 35.736 min, 83% ee, $[\alpha]_D^{23}$ = -70.25 (*c* 1.785, $CHCl_3$). ^{1}H NMR (400 MHz, $CDCl_3$): δ 8.42 (d, *J* = 8.6 Hz, 1H), 7.77 – 7.71 (m, 3H), 7.68 (d, *J* = 8.2 Hz, 1H), 7.48 – 7.38 (m, 4H), 7.31 (t, *J* = 7.7 Hz, 2H), 7.21 – 7.15 (m, 3H), 7.15 – 7.04 (m, 3H), 4.88 (dd, *J* = 7.9, 6.3 Hz, 1H), 3.61 (dd, *J* = 17.2, 8.0 Hz, 1H), 3.53 (dd, *J* =

17.2, 6.2 Hz, 1H).^{13}C NMR (100 MHz, $CDCl_3$): δ 197.04, 141.18, 136.71, 134.45, 134.07, 133.26, 133.20, 131.34, 129.13, 128.63, 128.58, 128.47, 128.10, 127.82, 127.42, 126.82, 126.28, 125.81, 125.47, 48.89, 44.72. δ IR (neat): 3053, 3027, 2891, 2878, 1678, 1595, 1501, 1449, 1418, 1368, 1340, 1251, 1225, 1078, 1019, 1002, 981, 921, 801, 769, 750, 711, 689, 667, 642, 624, 599, 565, 549, 530, 419 cm^{-1}. MS (MALDI-TOF) *m/z*: $[M + Na]^+$ Calcd. for $C_{25}H_{20}NaOS$ 391.113; Found 391.133.

3-(Naphthalen-1-ylthio)-1-phenyl-3-(*m*-tolyl)propan-1-one (12b)

White solid, 66.79 mg, mp 56 °C, 87% yield in 46 h, in THF. HPLC (OD-H, 99:1 *n*-Hexane/Isopropanol, 0.8 mL/min, 220 nm): t_{minor} = 16.063 min, t_{major} = 14.139 min, 78% ee, $[\alpha]_D^{24}$ = -111.4 (*c* 1.660, $CHCl_3$). ^{1}H NMR (400 MHz, $CDCl_3$): δ 8.42 (d, *J* = 8.2 Hz, 1H), 7.75 (d, *J* = 7.4 Hz, 3H), 7.69 (d, *J* = 8.2 Hz, 1H), 7.54 – 7.38 (m, 4H), 7.31 (t, *J* = 7.6 Hz, 2H), 7.23 (t, *J* = 7.7 Hz, 1H), 7.02 (dd, *J* = 15.1, 7.7 Hz, 3H), 6.90 (d, *J* = 6.6 Hz, 1H), 4.95 – 4.76 (m, 1H), 3.62 (dd, *J* = 17.2, 8.1 Hz, 1H), 3.51 (dd, *J* = 17.2, 6.0 Hz, 1H), 2.17 (s, 3H).^{13}C NMR (100 MHz, $CDCl_3$): δ 197.12, 140.90, 138.03, 136.71, 134.41, 134.04, 133.19, 133.05, 131.47, 129.02, 128.58, 128.52, 128.31, 128.20, 128.07, 126.73, 126.22, 125.78, 125.43, 124.73, 48.29, 44.68, 21.38. IR (neat): 3054, 3022, 2920, 2852, 1681, 1594, 1502, 1448, 1415, 1363, 1334, 1249, 1216, 1055, 1019, 1000, 979, 877, 801, 784, 768, 754, 727, 713, 684, 638, 624, 602, 573, 534, 510, 443, 421 cm^{-1}. MS (MALDI-TOF) *m/z*: $[M + Na]^+$ Calcd. for $C_{26}H_{22}NaOS$ 405.129; Found 405.147.

3-(Naphthalen-1-ylthio)-1-phenyl-3-(*p*-tolyl)propan-1-one (12c)

White solid, 71.84 mg, mp 102 °C, 94% yield in 6 h, in DCM. HPLC (AD-H, 98:2 *n*-Hexane/Isopropanol, 0.8 mL/min, 254 nm): t_{minor} = 15.832 min, t_{major} = 14.441 min, 94% ee, $[\alpha]_D^{23}$ = -91.42 (*c* 0.480, $CHCl_3$). ^{1}H NMR (400 MHz, $CDCl_3$): δ 8.43 (d, *J* = 8.3 Hz, 1H), 7.77 – 7.70 (m, 3H), 7.68 (d, *J* = 8.2 Hz, 1H), 7.51 – 7.39 (m, 4H), 7.30 (t, *J* = 7.7 Hz, 2H), 7.26 – 7.19 (m, 1H), 7.14 – 7.06 (m, 2H), 6.95 (d, *J* = 7.9 Hz, 2H), 4.87 (dd, *J* = 8.3, 5.9 Hz, 1H), 3.60 (dd, *J* = 17.1, 8.3 Hz, 1H), 3.49 (dd, *J* = 17.1, 5.9 Hz, 1H), 2.19 (s, 3H). ^{13}C NMR (100 MHz, $CDCl_3$): δ 197.19, 138.06, 137.10, 136.73, 134.37, 134.09, 133.22, 132.89, 131.63, 129.21, 128.99, 128.61, 128.10, 127.68, 126.80, 126.27, 125.80, 125.51, 48.10, 44.86, 21.17. IR (neat): 3053, 3030, 2944, 2922, 2892, 2861, 1681, 1595, 1515, 1502, 1449, 1417, 1361, 1334, 1307, 1251, 1226, 1155, 1113, 1062, 1019, 1001, 978, 954, 918, 801, 770, 759, 733, 701, 684, 649, 623, 583, 564, 534, 521, 435, 417 cm^{-1}. MS (MALDI-TOF) *m/z*: $[M + Na]^+$ Calcd. for $C_{26}H_{22}NaOS$ 405.129; Found 405.190.

3-(Naphthalen-1-ylthio)-3-phenyl-1-(*p*-tolyl)propan-1-one (12d)

White solid, 76.31 mg, mp 110-112 °C, >99% yield in 42 h, in THF. HPLC (AD-H, 98:2 *n*-Hexane/Isopropanol, 0.8 mL/min, 254 nm): t_{minor} = 22.531 min, t_{major} = 20.044 min, 91% ee, $[\alpha]_D^{24}$ = -74.25 (*c* 1.910, $CHCl_3$). ^{1}H NMR (400 MHz, $CDCl_3$): δ 8.41 (d, *J* = 8.3 Hz, 1H), 7.79 – 7.57 (m, 4H), 7.42 (dq, *J* = 15.4, 6.8 Hz, 3H), 7.27 – 6.98 (m, 8H), 4.88 (dd, *J* = 7.7, 6.4 Hz, 1H), 3.53 (qd, *J* = 17.1, 7.1 Hz, 2H), 2.26 (s, 3H). ^{13}C NMR (100 MHz, $CDCl_3$): δ 196.64, 144.06, 141.25, 134.44, 134.31, 134.06, 133.11, 131.43, 129.29, 129.04, 128.53, 128.41, 128.22, 127.80, 127.35, 126.76, 126.22, 125.81, 125.43, 48.48, 44.57, 21.66. IR (neat): 3053, 3029, 2962, 2894, 1666, 1604, 1498, 1456, 1421, 1362, 1328, 1307, 1230, 118, 1019, 972, 942, 815, 800, 767, 698, 666, 594,

559, 524, 491, 456, 416 cm^{-1}. MS (MALDI-TOF) *m/z*: $[M + Na]^+$ Calcd. for $C_{26}H_{22}NaOS$ 405.129; Found 405.147.

3-(3-Methoxyphenyl)-3-(naphthalen-1-ylthio)-1-phenylpropan-1-one (12e)

White solid, 76.46 mg, mp 46-47 °C, 73% yield in 24 h, in DCM. HPLC (OD-H, 90:10 *n*-Hexane/Isopropanol, 1 mL/min, 230 nm): t_{minor} = 10.575 min, t_{major} = 9.406 min, 63% ee, $[\alpha]_D^{24}$ = -92.87 (*c* 2.100, $CHCl_3$). ^1H NMR (400 MHz, $CDCl_3$): δ 8.42 (d, *J* = 8.2 Hz, 1H), 7.75 (t, *J* = 7.3 Hz, 3H), 7.69 (d, *J* = 8.2 Hz, 1H), 7.52 – 7.38 (m, 4H), 7.32 (t, *J* = 7.6 Hz, 2H), 7.26 – 7.19 (m, 1H), 7.05 (t, *J* = 7.9 Hz, 1H), 6.79 (d, *J* = 7.6 Hz, 1H), 6.67 (d, *J* = 17.9 Hz, 1H), 6.62 (dd, *J* = 8.1, 2.0 Hz, 1H), 4.93 – 4.79 (m, 1H), 3.68 – 3.48 (m, 5H). ^{13}C NMR (100 MHz, $CDCl_3$): δ 195.52, 158.04, 141.25, 135.25, 132.97, 132.57, 131.76, 131.73, 129.85, 127.96, 127.62, 127.14, 127.03, 126.62, 125.30, 124.76, 124.31, 123.98, 118.59, 111.92, 111.54, 53.69, 46.89, 43.17. IR (neat): 3055, 3001, 2959, 2918, 2836, 1710, 1685, 1597, 1489, 1448, 1359, 1260, 1221, 1157, 1090, 1042, 981, 873, 774, 692, 530 cm^{-1}. MS (MALDI-TOF) *m/z*: $[M + Na]^+$ Calcd. for $C_{26}H_{22}NaO_2S$ 421.124; Found 421.137.

3-(4-Methoxyphenyl)-3-(naphthalen-1-ylthio)-1-phenylpropan-1-one (12f)

White solid, 74.21 mg, mp 102 °C, 93% yield in 23 h, in DCM. HPLC (AD-H, 90:10 *n*-Hexane/Isopropanol, 1 mL/min, 220 nm): t_{minor} = 15.595 min, t_{major} = 13.816 min, 84% ee, $[\alpha]_D^{23}$ = -120.0 (*c* 1.845, $CHCl_3$). ^1H NMR (400 MHz, $CDCl_3$): δ 8.42 (d, *J* = 8.3 Hz, 1H), 7.79 – 7.69 (m, 3H), 7.67 (d, *J* = 8.2 Hz, 1H), 7.51 – 7.37 (m, 4H), 7.29 (t, *J* = 7.7 Hz, 2H), 7.21 (dd, *J* = 8.0, 7.4 Hz, 1H), 7.13 – 7.07 (m, 2H), 6.73 – 6.60 (m, 2H), 4.86 (dd, *J* = 8.3, 5.9 Hz, 1H), 3.63 (s, *J* = 6.0 Hz, 3H), 3.58 (dd, *J* = 17.1, 8.4 Hz, 1H), 3.47 (dd, J = 17.1, 5.9 Hz, 1H). ^{13}C NMR (100 MHz, $CDCl_3$): δ 197.17, 158.82, 136.76, 134.42, 133.17, 133.11, 133.04, 131.56, 128.98, 128.86, 128.58, 128.53, 128.06, 126.74, 126.22, 125.79, 125.46, 113.84, 55.23, 47.90, 44.90. IR (neat): 3051, 3006, 2953, 2931, 2891, 2834, 1681, 1609, 1594, 1512, 1448, 1415, 1363, 1334, 1293, 1247, 1223, 1176, 1107, 1060, 1029, 1000, 977, 954, 918, 850, 813, 800, 767, 736, 723, 699, 684, 667, 647, 621, 588, 564, 529, 427, 414 cm^{-1}. MS (MALDI-TOF) *m/z*: $[M + Na]^+$ Calcd. for $C_{26}H_{22}NaO_2S$ 421.124; Found 421.154.

3-(Naphthalen-1-ylthio)-1-phenyl-3-(3,4,5-trimethoxyphenyl)propan-1-one (12g)

White solid, 91.53 mg, mp 115-116 °C, >99% yield in 24 h, in DCM. HPLC (AD-H, 90:10 *n*-Hexane/Isopropanol, 1 mL/min, 230 nm): t_{minor} = 22.490 min, t_{major} = 16.387 min, 96% ee, $[\alpha]_D^{23}$ = -123.30 (*c* 2.410, $CHCl_3$). ^1H NMR (400 MHz, $CDCl_3$): δ 8.36 (d, *J* = 8.1 Hz, 1H), 7.84 – 7.76 (m, 2H), 7.76 – 7.66 (m, 2H), 7.52 – 7.37 (m, 4H), 7.34 (t, *J* = 7.6 Hz, 2H), 7.23 (t, *J* = 7.7 Hz, 1H), 6.27 (s, 2H), 4.80 (t, *J* = 7.1 Hz, 1H), 3.66 (s, 3H), 3.63 – 3.48 (m, 8H). ^{13}C NMR (100 MHz, $CDCl_3$): δ 195.70, 151.59, 135.82, 135.43, 135.31, 133.32, 132.67, 132.39, 131.99, 129.83, 127.91, 127.33, 127.12, 126.76, 125.39, 124.92, 124.48, 124.14, 103.46, 59.47, 54.67, 47.61, 43.06. IR (neat): 3057, 3001, 2970, 2934, 2899, 2838, 2827, 1682, 1590, 1513, 1454, 1430, 1366, 1335, 1320, 1254, 1223, 1182, 1125, 1008, 899, 805, 768, 754, 722, 688, 647, 557, 522, 416 cm^{-1}. MS (MALDI-TOF) *m/z*: $[M + Na]^+$ Calcd. for $C_{28}H_{26}NaO_4S$ 481.145; Found 481.137.

3-(2-Chlorophenyl)-3-(naphthalen-1-ylthio)-1-phenylpropan-1-one (12h)

White solid, 67.65 mg, mp 54-55 °C, 84% yield in 23 h, in THF. HPLC (OD-H, 90:10 *n*-Hexane/Isopropanol, 1 mL/min, 254 nm): t_{minor} = 7.968 min, t_{major} = 9.813 min, 66% ee, $[\alpha]_D^{24}$ = -4.622 (*c* 1.350, $CHCl_3$). ^1H NMR (400 MHz, $CDCl_3$): δ 8.73 – 8.41 (m, 1H), 7.97 – 7.79 (m, 4H), 7.68 – 7.52 (m, 4H), 7.50 – 7.32 (m, 5H), 7.18 (dd, *J* = 8.8, 5.1 Hz, 2H), 5.54 (t, *J* = 7.1 Hz, 1H), 3.72 (qd, *J* = 17.3, 7.2 Hz, 2H). ^{13}C NMR (100 MHz, $CDCl_3$): δ196.58, 138.47, 136.51, 134.66, 134.00, 133.91, 133.51, 133.26, 130.73, 129.80, 129.32, 128.61, 128.42, 128.41, 128.08, 126.90, 126.73, 126.24, 125.82, 125.40, 44.68, 44.15. IR (neat): 3052, 2916, 2894, 1687, 1581, 1502, 1472, 1447, 1357, 1312, 1264, 1243, 1202, 1160, 1061, 1032, 981, 911, 797, 770, 749, 732, 679, 650, 588, 557, 538, 503, 459, 418 cm^{-1}. MS (MALDI-TOF) *m/z*: $[M + Na]^+$ Calcd. for $C_{25}H_{19}ClNaOS$ 425.074; Found 425.131.

3-(3-Chlorophenyl)-3-(naphthalen-1-ylthio)-1-phenylpropan-1-one (12i)

White solid, 63.78 mg, mp 85 °C, 79% yield in 22 h, in THF. HPLC (OD-H, 95:5 *n*-Hexane/Isopropanol, 1 mL/min, 220 nm): t_{minor} = 9.514 min, t_{major} = 8.199 min, 85% ee, $[\alpha]_D^{23}$ = -132.7 (*c* 1.590, $CHCl_3$). ^1H NMR (400 MHz, $CDCl_3$): δ 8.39 (d, *J* = 8.3 Hz, 1H), 7.88 – 7.64 (m, 4H), 7.54 – 7.38 (m, 4H), 7.33 (t, *J* = 7.7 Hz, 2H), 7.26 – 7.19 (m, 1H), 7.17 (s, 1H), 7.10 – 6.92 (m, 3H), 4.82 (t, *J* = 7.1 Hz, 1H), 3.67 – 3.45 (m, 2H). ^{13}C NMR (100 MHz, $CDCl_3$): δ 196.56, 143.36, 136.54, 134.49, 134.18, 134.08, 133.68, 133.38, 130.65, 129.55, 129.48, 128.67, 128.57, 128.07, 127.85, 127.49, 126.90, 126.31, 126.08, 125.68, 125.40, 47.89, 44.39. IR (neat): 3054, 3029, 2951, 2920, 2851, 1681, 1593, 1570, 1500, 1431, 1412, 1364, 1333, 1254, 1226, 1185, 1163, 1076, 1001, 984, 904, 834, 798, 769, 752, 729, 683, 64, 625, 604, 533, 508, 436, 419 cm^{-1}. MS (MALDI-TOF) *m/z*: $[M + Na]^+$ Calcd. for $C_{25}H_{19}ClNaOS$ 425.074; Found 425.087.

3-(4-Chlorophenyl)-3-(naphthalen-1-ylthio)-1-phenylpropan-1-one (12j)

White solid, 80.50 mg, mp 121-122 °C, >99% yield in 21 h, in THF. HPLC (AD-H, 90:10 *n*-Hexane/Isopropanol, 1 mL/min, 220 nm): t_{minor} = 11.144 min, t_{major} = 9.939 min, 71% ee, $[\alpha]_D^{24}$ = -125.2 (*c* 2.090, $CHCl_3$). ^1H NMR (400 MHz, $CDCl_3$): δ 8.40 (d, *J* = 8.2 Hz, 1H), 7.88 – 7.72 (m, 3H), 7.70 (d, *J* = 8.2 Hz, 1H), 7.55 – 7.38 (m, 4H), 7.33 (t, *J* = 7.7 Hz, 2H), 7.21 (dd, *J* = 14.7, 6.8 Hz, 1H), 7.13 – 7.03 (m, 4H), 4.83 (dd, *J* = 7.7, 6.5 Hz, 1H), 3.70 – 3.42 (m, 2H). ^{13}C NMR (100 MHz, $CDCl_3$): δ 196.69, 139.85, 136.58, 134.45, 134.09, 133.53, 133.38, 133.00, 130.81, 129.40, 129.14, 128.68, 128.62, 128.54, 128.07, 126.93, 126.33, 125.70, 125.46, 77.42, 77.10, 76.78, 47.75, 44.52. IR (neat): 3050, 3028, 2926, 2897, 1679, 1595, 1493, 1450, 1418, 1359, 1332, 1226, 1154, 1091, 1015, 976, 954, 917, 853, 815, 799, 769, 751, 732, 683, 656, 619, 571, 531, 505, 430 cm^{-1}. MS (MALDI-TOF) *m/z*: $[M + Na]^+$ Calcd. for $C_{25}H_{19}ClNaOS$ 425.074; Found 425.086.

3-(3-Bromophenyl)-3-(naphthalen-1-ylthio)-1-phenylpropan-1-one (12k)

White solid, 59.28 mg, mp 84 °C, 66% yield in 40 h, in THF. HPLC (OD-H, 90:10 *n*-Hexane/Isopropanol, 1 mL/min, 254 nm): t_{minor} = 8.699 min, t_{major} = 7.584 min, 51% ee, $[\alpha]_D^{24}$ = -145.1 (*c* 1.480, $CHCl_3$). ^1H NMR (400 MHz, $CDCl_3$): δ 8.39 (d, *J* = 8.2 Hz, 1H), 7.82 – 7.63 (m, 4H), 7.43 (dd, *J* = 15.1, 7.6 Hz, 4H), 7.37 – 7.27 (m, 3H), 7.29 – 7.11 (m, 2H), 7.03 (d, *J* = 7.6 Hz,

1H), 6.94 (t, J = 7.8 Hz, 1H), 4.80 (t, J = 7.0 Hz, 1H), 3.55 (d, J = 7.0 Hz, 2H). ^{13}C NMR (100 MHz, $CDCl_3$): δ 195.46, 142.56, 135.43, 133.42, 133.00, 132.66, 132.32, 129.68, 129.53, 129.33, 128.77, 128.44, 127.60, 127.51, 127.00, 125.84, 125.47, 125.25, 124.61, 124.34, 121.33, 46.78, 43.28. IR (neat): 3056, 3041, 2927, 2892, 1680, 1594, 1565, 1501, 1475, 1448, 1429, 1415, 1357, 1329, 1219, 1151, 1061, 983, 953, 918, 878, 811, 795, 768, 753, 716, 685, 667, 638, 604, 570, 533, 508, 445, 433, 419 cm^{-1}. MS (MALDI-TOF) *m/z*: $[M + Na]^+$ Calcd. for $C_{25}H_{19}BrNaOS$ 469.024; Found 469.024.

1-(4-Bromophenyl)-3-(naphthalen-1-ylthio)-3-phenylpropan-1-one (12l)

White solid, 78.24 mg, mp 162-163 °C, >99% yield in 23 h, in THF. HPLC (AD-H, 98:2 *n*-Hexane/Isopropanol, 1 mL/min, 254 nm): t_{minor} = 16.944 min, t_{major} = 17.723 min, 82% ee, $[\alpha]_D^{24}$ = -53.21 (c 1.260, $CHCl_3$). ^{1}H NMR (400 MHz, $CDCl_3$): δ 8.40 (d, J = 8.2 Hz, 1H), 7.70 (dd, J = 23.8, 8.0 Hz, 2H), 7.56 (d, J = 8.4 Hz, 2H), 7.51 – 7.31 (m, 5H), 7.27 – 6.99 (m, 6H), 4.84 (t, J = 7.0 Hz, 1H), 3.65 – 3.31 (m, 2H). ^{13}C NMR (100 MHz, $CDCl_3$): δ 194.26, 139.14, 133.59, 132.64, 132.26, 131.53, 130.11, 129.36, 127.79, 127.43, 126.79, 126.70, 126.65, 125.95, 125.71, 125.05, 124.50, 123.94, 123.65, 46.60, 42.89. IR (neat): 3055, 3030, 2899, 1682, 1583, 1500, 1452, 1396, 1365, 1331, 1220, 1177, 1070, 1007, 983, 823, 799, 774, 721, 699, 661, 626, 601, 557, 524, 470, 448, 419 cm^{-1}. MS (MALDI-TOF) *m/z*: $[M + Na]^+$ Calcd. for $C_{25}H_{19}BrNaOS$ 469.024; Found 469.058.

3-(Naphthalen-1-ylthio)-1-phenyl-3-(4-(trifluoromethyl)phenyl)propan-1-one (12m)

White solid, 79.57 mg, mp 118 °C, 79% yield in 19 h, in THF. HPLC (AD-H, 90:10 *n*-Hexane/Isopropanol, 1 mL/min, 254 nm): t_{minor} = 8.847 min, t_{major} = 7.195 min, 67% ee, $[\alpha]_D^{23}$ = -74.02 (c 1.730, $CHCl_3$). ^{1}H NMR (400 MHz, $CDCl_3$): δ 8.37 (d, J = 7.8 Hz, 1H), 7.88 – 7.68 (m, 4H), 7.54 – 7.30 (m, 8H), 7.28 – 7.16 (m, 3H), 4.89 (t, J = 7.1 Hz, 1H), 3.78 – 3.50 (m, 2H). ^{13}C NMR (100 MHz, $CDCl_3$): δ 195.41, 144.34, 135.38, 133.42, 133.01, 132.74, 132.41, 129.34, 128.55, 128.33 (q, J = 32.3 Hz), 127.64, 127.54, 127.02, 126.99, 125.89, 125.29, 124.54, 124.34, 124.25 (dd, J = 7.5, 3.8 Hz), 46.87, 43.15. IR (neat): 3050, 2939, 2902, 1677, 1618, 1596,1503, 1450, 1426, 1365, 1328, 1226, 1157, 1123, 1072, 1018, 986, 954, 918, 860, 821, 797, 769, 758, 708, 684, 647, 634, 620, 601, 563, 529, 425 cm^{-1}. MS (MALDI-TOF) *m/z*: $[M + Na]^+$ Calcd. for $C_{26}H_{19}F_3NaOS$ 459.101; Found 459.123.

3-(Naphthalen-1-ylthio)-1-(2-nitrophenyl)-3-phenylpropan-1-one (12n)

White solid, 73.61 mg, mp 112 °C, 84% yield in 21 h, in THF. HPLC (AD-H, 99:1 *n*-Hexane/Isopropanol, 0.8 mL/min, 254 nm): t_{minor} = 64.775 min, t_{major} = 68.571 min, 82% ee, $[\alpha]_D^{24}$ = -1.545 (c 1.165, $CHCl_3$). ^{1}H NMR (400 MHz, $CDCl_3$): δ 8.52 – 8.22 (m, 1H), 8.07 – 7.82 (m, 1H), 7.78 – 7.60 (m, 2H), 7.55 – 7.31 (m, 5H), 7.20 (t, J = 7.7 Hz, 1H), 7.16 – 7.00 (m, 5H), 6.94 – 6.81 (m, 1H), 4.75 (t, J = 7.3 Hz, 1H), 3.41 (t, J = 8.7 Hz, 2H). ^{13}C NMR (100 MHz, $CDCl_3$): 198.21, 143.90, 138.96, 136.14, 132.76, 132.67, 132.51, 131.58, 129.39, 129.05,127.05, 126.96, 126.27, 126.08, 126.05, 125.31, 124.74, 124.09 123.93, 122.74, 47.22, 46.93. IR (neat): 3058, 3029, 2923, 2910, 2856, 1709, 1573, 1529, 1499, 1455, 1403, 1367, 1343, 1218, 1142, 1019, 987, 935, 856, 790, 767, 747, 732, 715, 697, 668, 634, 618, 572, 547, 508, 451, 420 cm^{-1}. (MALDI-TOF) *m/z*: $[M + Na]^+$ Calcd. for $C_{25}H_{19}NNaO_3S$ 436.098; Found 436.139.

3-(Naphthalen-1-ylthio)-3-(4-nitrophenyl)-1-phenylpropan-1-one (12o)

Yellow solid, 67.23 mg, mp 129-130 °C, 81% yield in 21 h, in THF. HPLC (AD-H, 90:10 *n*-Hexane/Isopropanol, 1 mL/min, 210 nm): t_{minor} = 26.461 min, t_{major} = 24.532 min, 68% ee, $[\alpha]_D^{23}$ = -153.6 (*c* 1.665, $CHCl_3$). ^{1}H NMR (400 MHz, $CDCl_3$): δ 8.37 (d, *J* = 8.1 Hz, 1H), 7.92 (d, *J* = 8.7 Hz, 2H), 7.83 – 7.68 (m, 4H), 7.54 – 7.40 (m, 3H), 7.37 (t, *J* = 7.6 Hz, 3H), 7.26 – 7.15 (m, 3H), 4.90 (t, *J* = 7.1 Hz, 1H), 3.74 – 3.55 (m, 2H). ^{13}C NMR (100 MHz, $CDCl_3$): δ 195.06, 148.01, 145.81, 135.24, 133.43, 133.12, 133.05, 132.59, 128.90, 128.76, 127.73, 127.66, 127.54, 127.01, 126.05, 125.40, 124.47, 124.36, 122.50, 46.75, 42.93. IR (neat): 3073, 3048, 2962, 2900, 2851, 1673, 1595, 1515, 1449, 1260, 1227, 1107, 1016, 975, 956, 918, 857, 798, 796, 751, 716, 682, 647, 618, 566, 536, 514, 427 cm^{-1}. MS (MALDI-TOF) *m/z*: $[M + Na]^+$ Calcd. for $C_{25}H_{19}NNaO_3S$ 436.098; Found 436.151.

3-(Naphthalen-1-ylsulfonyl)-1,3-diphenylpropan-1-one (13a)

White solid, 17.22 mg, mp 135 °C, 43% yield. HPLC (IA, 95:5 *n*-Hexane/Isopropanol, 1 mL/min, 220 nm): t_{minor} = 44.650 min, t_{major} = 47.501 min, 86% ee, $[\alpha]_D^{22}$ = -156.9 (*c* 1.140, $CHCl_3$). ^{1}H NMR (400 MHz, $CDCl_3$): δ 8.74 (d, *J* = 8.6 Hz, 1H), 7.92 (dd, *J* = 28.8, 7.8 Hz, 4H), 7.75 (d, *J* = 7.3 Hz, 1H), 7.69 – 7.62 (m, 1H), 7.59 – 7.48 (m, 2H), 7.44 – 7.36 (m, *J* = 7.7 Hz, 2H), 7.25 (t, *J* = 7.8 Hz, 1H), 7.13 – 6.90 (m, *J* = 28.3, 13.6, 7.1 Hz, 5H), 5.22 (dd, *J* = 9.2, 3.8 Hz, 1H), 4.15 (dd, *J* = 17.9, 3.8 Hz, 1H), 3.94 (dd, *J* = 18.0, 9.3 Hz, 1H). ^{13}C NMR (100 MHz, $CDCl_3$): δ 197.99, 136.16, 135.25, 133.87, 133.68, 132.56, 131.98, 129.41, 129.36, 139.13, 128.84, 128.75, 128.70, 128.33, 128.16, 126.97, 124.32, 123.93, 65.74, 36.79. IR (neat): 3034, 2957, 2915, 2852, 1685, 1596, 1505, 1449, 1420, 1363, 1341, 1303, 1235, 1196, 1150, 1025, 981, 921, 826, 801, 768, 750, 699, 686, 629, 594, 573, 555, 525, 506, 476 cm^{-1}. HRMS (ESI-TOF) *m/z*: $[M + Na]^+$ Calcd. for $C_{25}H_{20}NaO_3S$ 423.1031; Found 423.1022.

3-(3-Methoxyphenyl)-3-(naphthalen-1-ylsulfonyl)-1-phenylpropan-1-one (13b)

White solid, 19.41 mg, mp 93 °C, 45% yield. HPLC (IA, 95:5 *n*-Hexane/Isopropanol, 1 mL/min, 254 nm): t_{minor} = 18.229 min, t_{major} = 20.872 min, 68% ee, $[\alpha]_D^{23}$ = -115.5 (*c* 1.313, $CHCl_3$). ^{1}H NMR (400 MHz, $CDCl_3$): δ. ^{13}C NMR (100 MHz, $CDCl_3$): δ 8.74 (d, *J* = 8.7 Hz, 1H), 7.96 (d, *J* = 8.2 Hz, 1H), 7.88 (t, *J* = 7.5 Hz, 3H), 7.80 (d, *J* = 7.4 Hz, 1H), 7.65 (t, *J* = 7.8 Hz, 1H), 7.58 – 7.48 (m, 2H), 7.40 (t, *J* = 7.6 Hz, 2H), 7.28 (t, *J* = 7.8 Hz, 1H), 6.91 (t, *J* = 8.0 Hz, 1H), 6.65 – 6.51 (m, 2H), 6.41 (s, *J* = 9.9 Hz, 1H), 5.18 (dd, *J* = 9.3, 3.8 Hz, 1H), 4.12 (dd, *J* = 17.9, 3.9 Hz, 1H), 3.93 (dd, *J* = 18.0, 9.3 Hz, 1H), 3.42 (s, *J* = 10.6 Hz, 3H).. ^{13}C NMR (100 MHz, $CDCl_3$): δ 192.87, 157.18, 134.08, 133.07, 131.84, 131.73, 131.54, 129.96, 129.86, 127.34, 127.17, 126.94, 126.69, 126.63, 126.06, 124.84, 122.24, 121.88, 119.52, 112.64, 112.59, 63.71, 52.88, 34.58. IR (neat): 3053, 2960, 2918, 2849, 1726, 1685, 1594, 1493, 1447, 1362, 1305, 1258, 1231, 1149, 1121, 1035, 883, 792, 770, 688, 679, 633, 619, 593, 552, 528, 504, 486 cm^{-1}. HRMS (ESI-TOF) *m/z*: $[M]^+$ Calcd. for $C_{26}H_{22}O_4S$ 430.1239; Found 439.1239.

3-(3-Methoxyphenyl)-3-(naphthalen-1-ylsulfonyl)-1-phenylpropan-1-one (13c)

White solid, 26.61 mg, mp 146 °C, 62% yield. HPLC (IA, 90:10 *n*-Hexane/Isopropanol, 1 mL/min, 254 nm): t_{minor} = 40.307 min, t_{major} = 45.189 min, 86% ee, $[\alpha]_D^{23}$ = -173.1 (*c* 1.773, $CHCl_3$). ^{1}H NMR

(400 MHz, $CDCl_3$): δ 8.73 (d, *J* = 8.6 Hz, 1H), 7.96 (d, *J* = 8.2 Hz, 1H), 7.87 (d, *J* = 7.7 Hz, 3H), 7.78 (d, *J* = 7.3 Hz, 1H), 7.66 (t, *J* = 7.8 Hz, 1H), 7.59 – 7.49 (m, 2H), 7.39 (t, *J* = 7.6 Hz, 2H), 7.29 (t, *J* = 7.8 Hz, 1H), 6.88 (d, *J* = 8.6 Hz, 2H), 6.53 (d, *J* = 8.7 Hz, 2H), 5.16 (dd, *J* = 9.4, 3.7 Hz, 1H), 4.09 (dd, *J* = 17.9, 3.8 Hz, 1H), 3.90 (dd, *J* = 17.9, 9.5 Hz, 1H), 3.61 (s, 3H), 1.51 (s, *J* = 19.6 Hz, 3H). ^{13}C NMR (100 MHz, $CDCl_3$): δ 194.08, 158.82, 135.20, 134.14, 132.87, 132.61, 131.13, 130.94, 129.55, 128.35, 128.08, 127.74, 127.71, 127.13, 125.90, 123.37, 123.31, 123.00, 112.79, 64.17, 54.14, 35.82. IR (neat): 3061, 3004, 2962, 2916, 2850, 1684, 1610, 1580, 1511, 1457, 1421, 1361, 1303, 1254, 1229, 1179, 1154, 1120, 1025, 979, 910, 852, 800, 768, 729, 711, 679, 639, 621, 600, 572, 552, 529, 505, 484, 440 cm^{-1}. HRMS (ESI-TOF) *m/z*: $[M + Na]^+$ Calcd. for; $C_{26}H_{22}NaO_4S$ 453.1136; Found 453.1164.

3-(4-Chlorophenyl)-3-(naphthalen-1-ylsulfonyl)-1-phenylpropan-1-one (13d)

White solid, 12.28 mg, mp 134 °C, 28% yield. HPLC (IA, 90:10 *n*-Hexane/Isopropanol, 1 mL/min, 254 nm): t_{minor} = 25.206 min, t_{major} = 31.435 min, 66% ee, $[\alpha]_D^{23}$ = -135.7 (*c* 1.040, $CHCl_3$). ^1H NMR (400 MHz, $CDCl_3$):): 8.71 (d, *J* = 8.7 Hz, 1H), 8.00 – 7.93 (m, *J* = 6.3 Hz, 1H), 7.90 – 7.82 (m, 3H), 7.77 (d, *J* = 7.4 Hz, 1H), 7.71 – 7.64 (m, 1H), 7.64 – 7.42 (m, 3H), 7.40 (t, *J* = 7.7 Hz, 2H), 7.35 – 7.27 (m, *J* = 15.3, 7.7 Hz, 1H), 6.93 (dd, *J* = 35.0, 8.5 Hz, 4H), 5.17 (dd, *J* = 9.6, 3.6 Hz, 1H), 4.01 (ddd, *J* = 27.6, 18.0, 6.6 Hz, 2H) . ^{13}C NMR (100 MHz, $CDCl_3$): δ 193.63, 134.88, 134.30, 133.67, 132.75, 132.65, 130.89, 130.61, 130.05, 129.50, 128.43, 128.09, 127.80, 127.64, 127.42, 126.98, 125.92, 123.00, 122.87, 63.96, 49.26, 35.60. IR (neat): 3059, 2917, 2849, 1681, 1658, 1593, 1492, 1447, 1420, 1363, 1329, 1142, 1119, 1094, 1015, 979, 924, 858, 798, 772, 775, 707, 689, 631, 620, 598, 564, 533, 507, 481, 456 cm^{-1}. HRMS (ESI-TOF) *m/z*: $[M + Na]^+$ Calcd. for $C_{25}H_{19}ClNaO_3S$ 457.0641; Found 457.0666.

III. ^{1}H and ^{13}C NMR spectra of catalyst 5

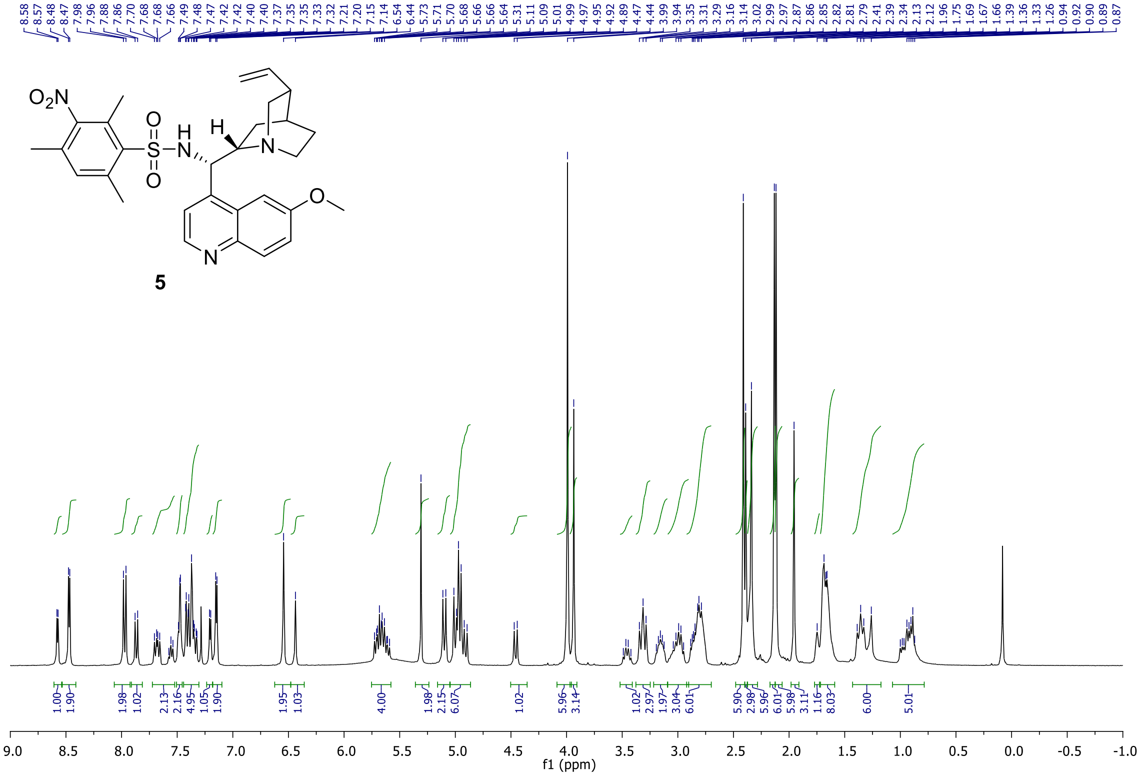

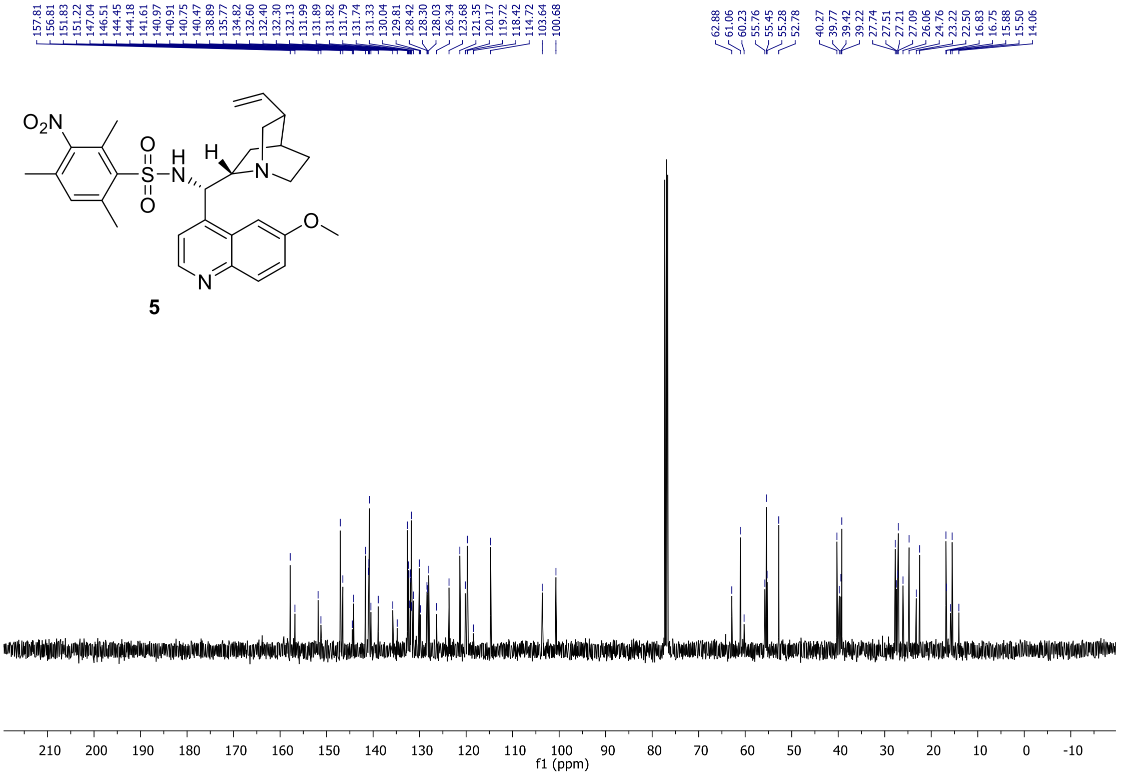

IV. ^{1}H and ^{13}C NMR spectra of products 12a–o

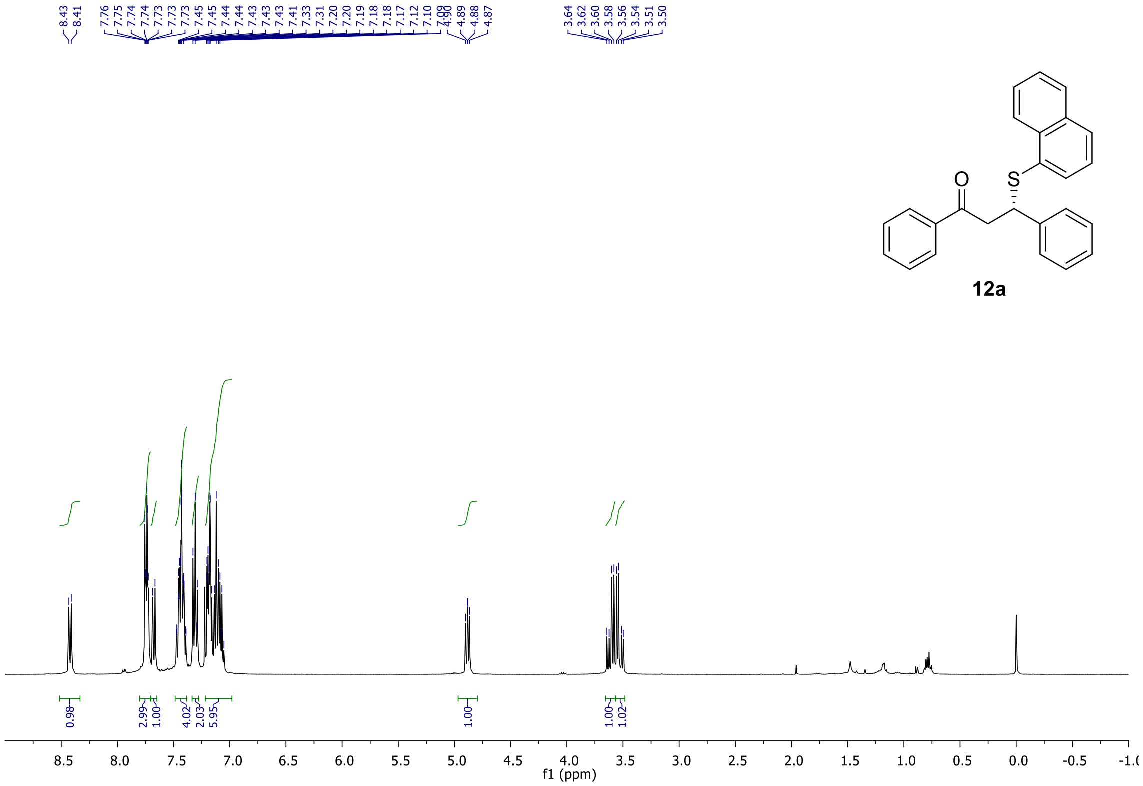

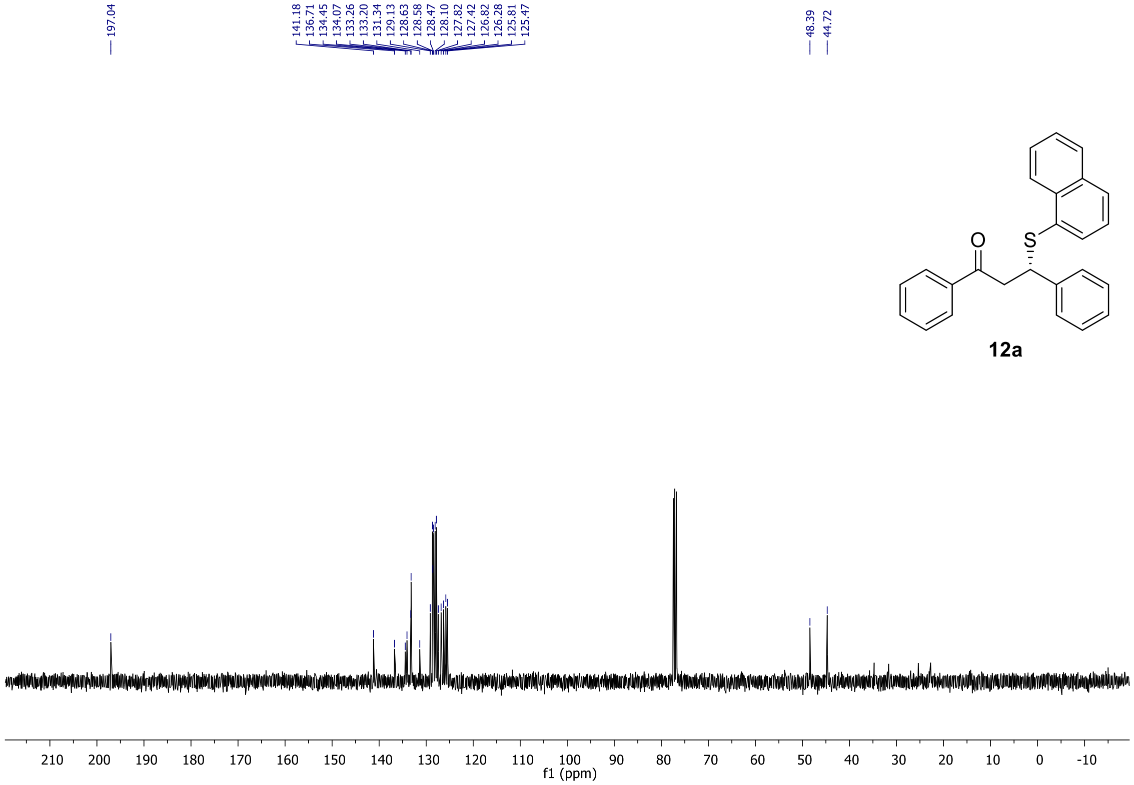

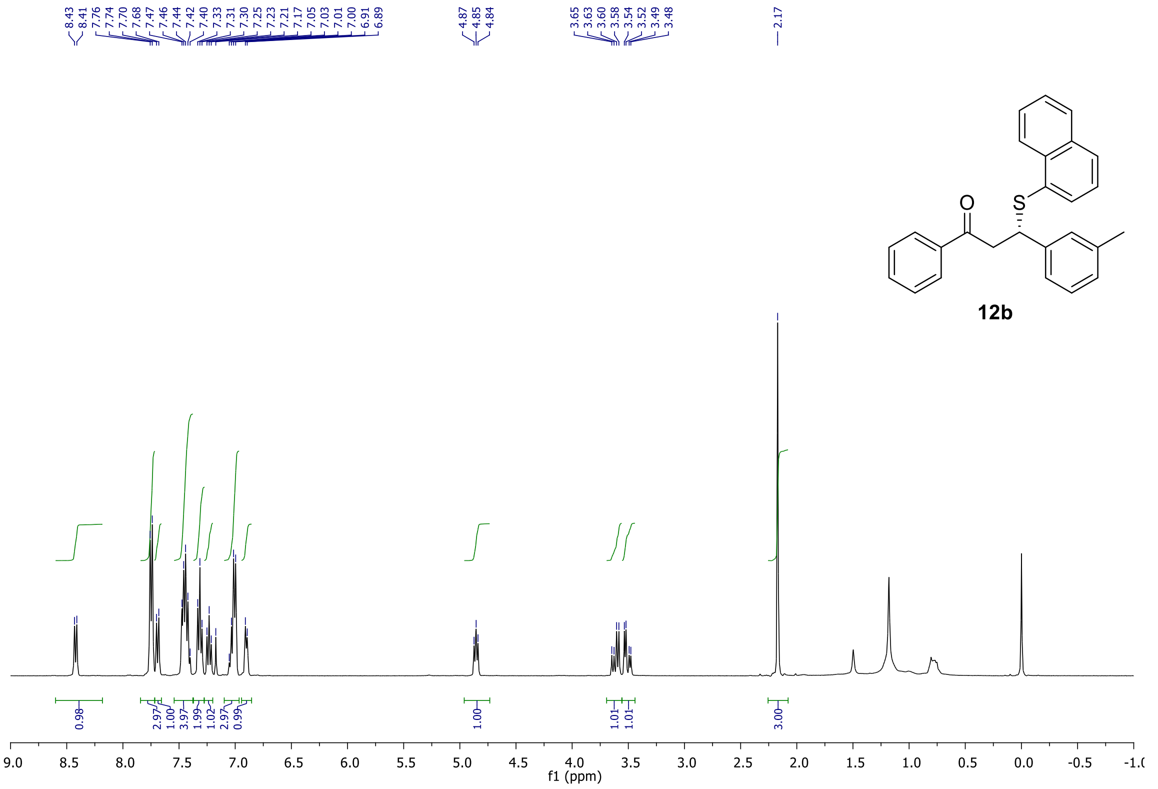

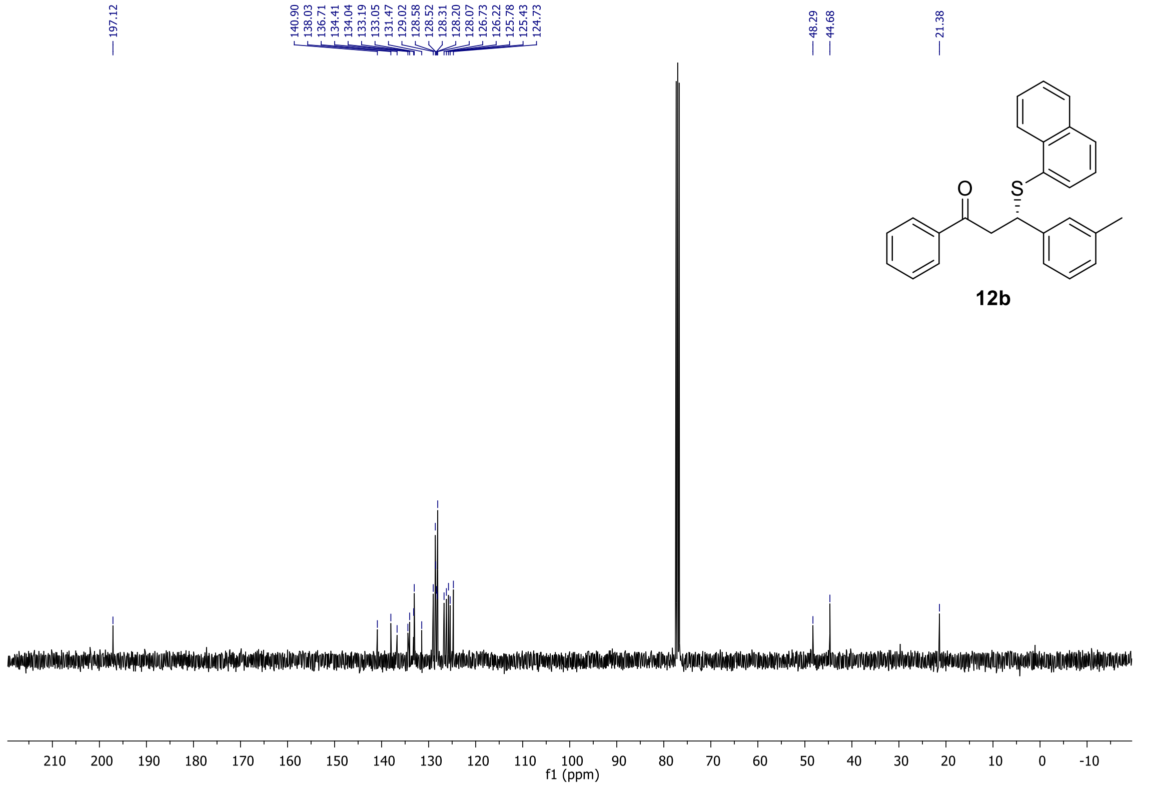

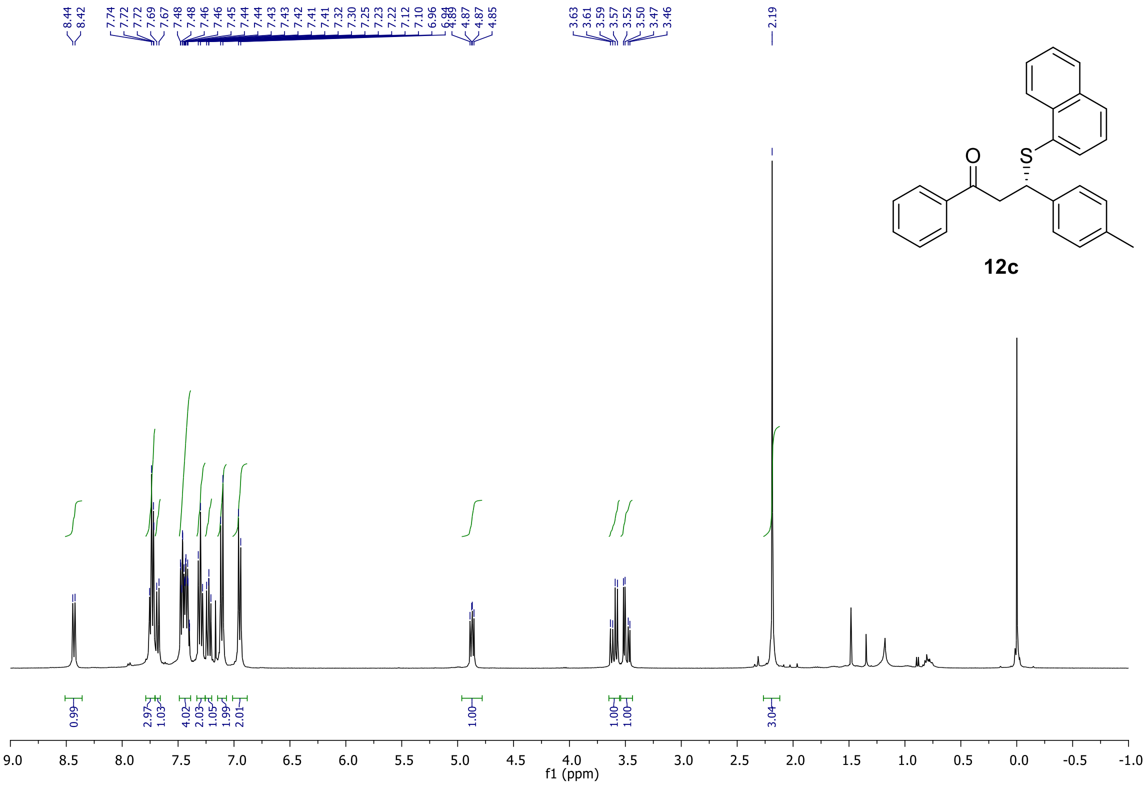
8.44
8.42
7.74
7.72
7.72
7.69
7.67
7.48
7.46
7.46
7.45
7.44
7.44
7.43
7.42
7.41
7.32
7.30
7.25
7.23
7.22
7.12
7.10
6.96
6.94
4.89
4.87
4.87
4.85
3.63
3.61
3.59
3.57
3.52
3.50
3.47
3.46
2.19
O
S
12c
0.99
2.97
1.03
4.02
2.03
1.05
1.99
2.01
1.00
1.00
1.00
3.04
9.0 8.5 8.0 7.5 7.0 6.5 6.0 5.5 5.0 4.5 4.0 3.5 3.0 2.5 2.0 1.5 1.0 0.5 0.0 -0.5 -1.0
f1 (ppm)

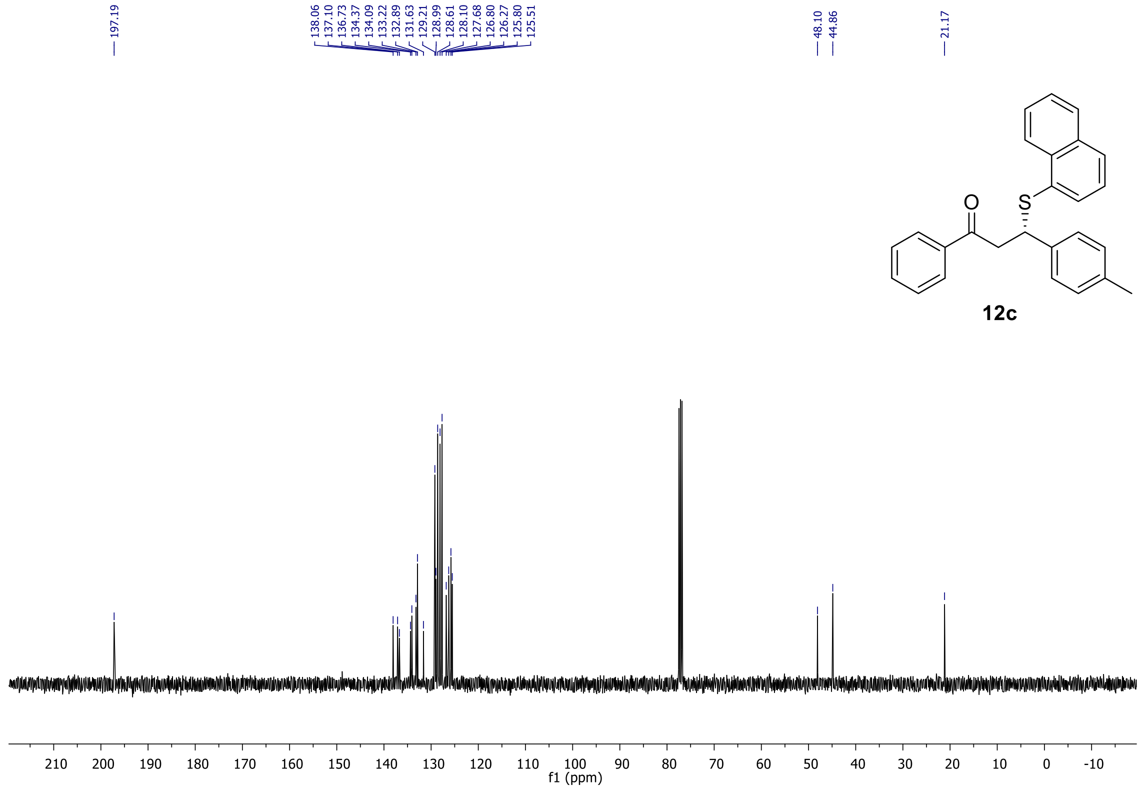
197.19
138.06
137.10
136.73
134.37
134.09
133.22
132.89
131.63
129.21
128.99
128.61
128.10
127.68
126.80
126.27
125.80
125.51
48.10
44.86
21.17
O
S
12c
210 200 190 180 170 160 150 140 130 120 110 100 90 80 70 60 50 40 30 20 10 0 -10
f1 (ppm)

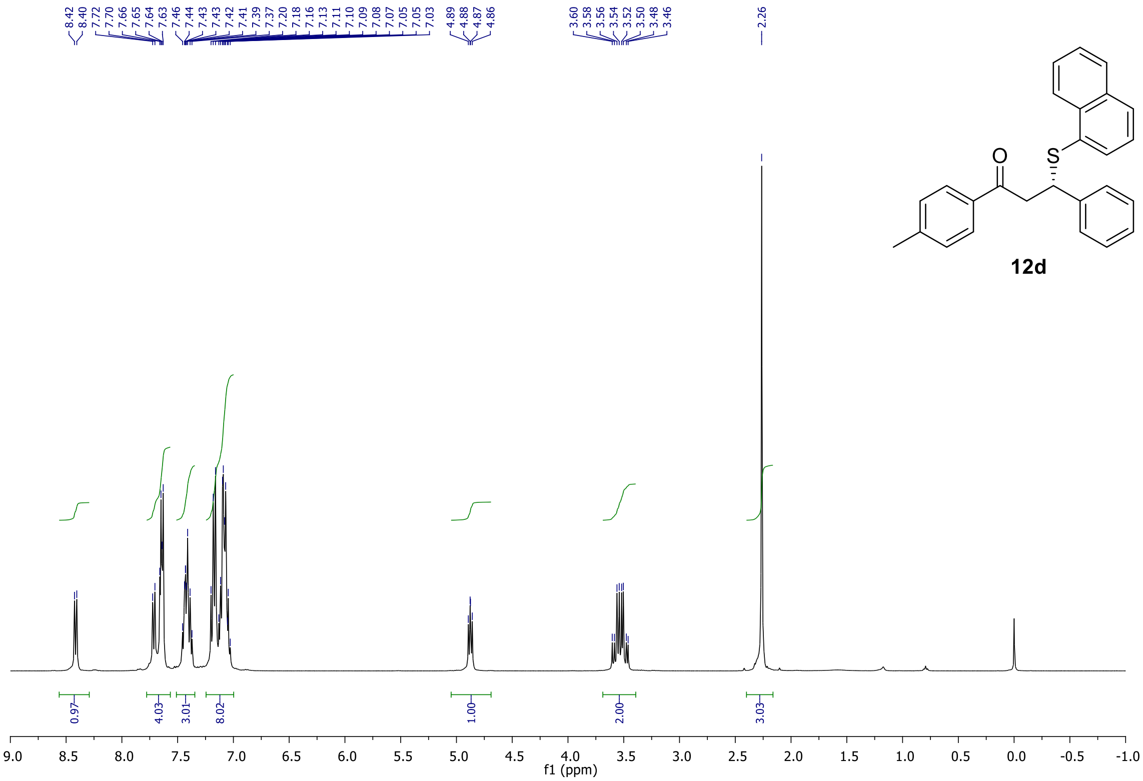
8.42
8.40
7.72
7.70
7.66
7.65
7.64
7.63
7.46
7.44
7.43
7.43
7.42
7.41
7.39
7.37
7.20
7.18
7.16
7.13
7.11
7.10
7.09
7.08
7.07
7.05
7.05
7.03
4.89
4.88
4.87
4.86
3.60
3.58
3.56
3.54
3.52
3.50
3.48
3.46
2.26
O
S
12d
0.97
4.03
3.01
8.02
1.00
2.00
3.03
9.0 8.5 8.0 7.5 7.0 6.5 6.0 5.5 5.0 4.5 4.0 3.5 3.0 2.5 2.0 1.5 1.0 0.5 0.0 -0.5 -1.0
f1 (ppm)

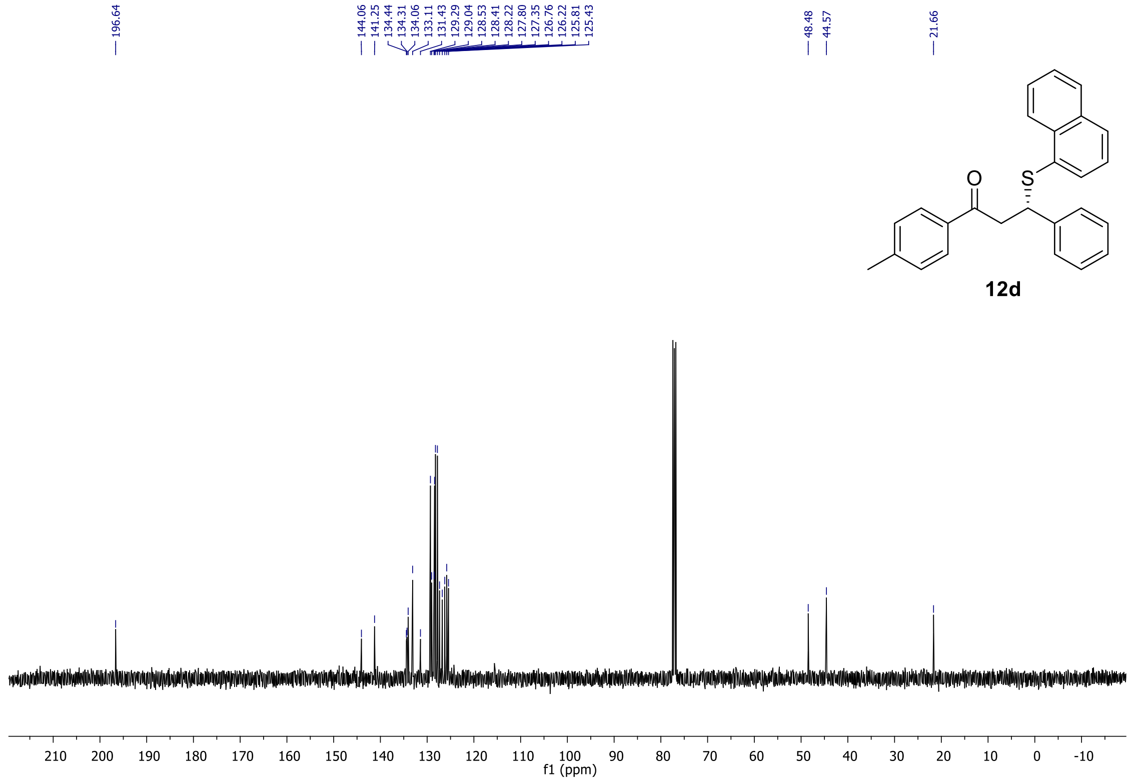
196.64
144.06
141.25
134.44
134.31
134.06
133.11
131.43
129.29
129.04
128.53
128.41
128.22
127.80
127.35
126.76
126.22
125.81
125.43
48.48
44.57
21.66
O
S
12d
210 200 190 180 170 160 150 140 130 120 110 100 90 80 70 60 50 40 30 20 10 0 -10
f1 (ppm)

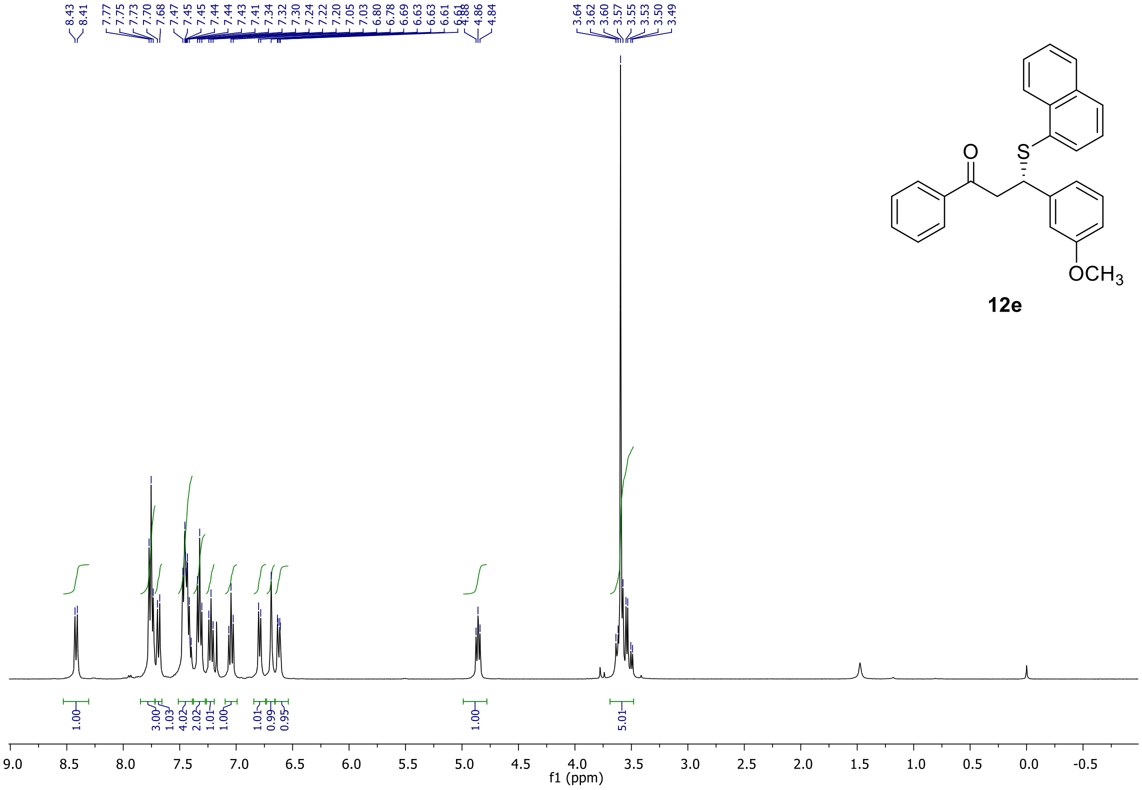
O
S
OCH3
12e
f1 (ppm)

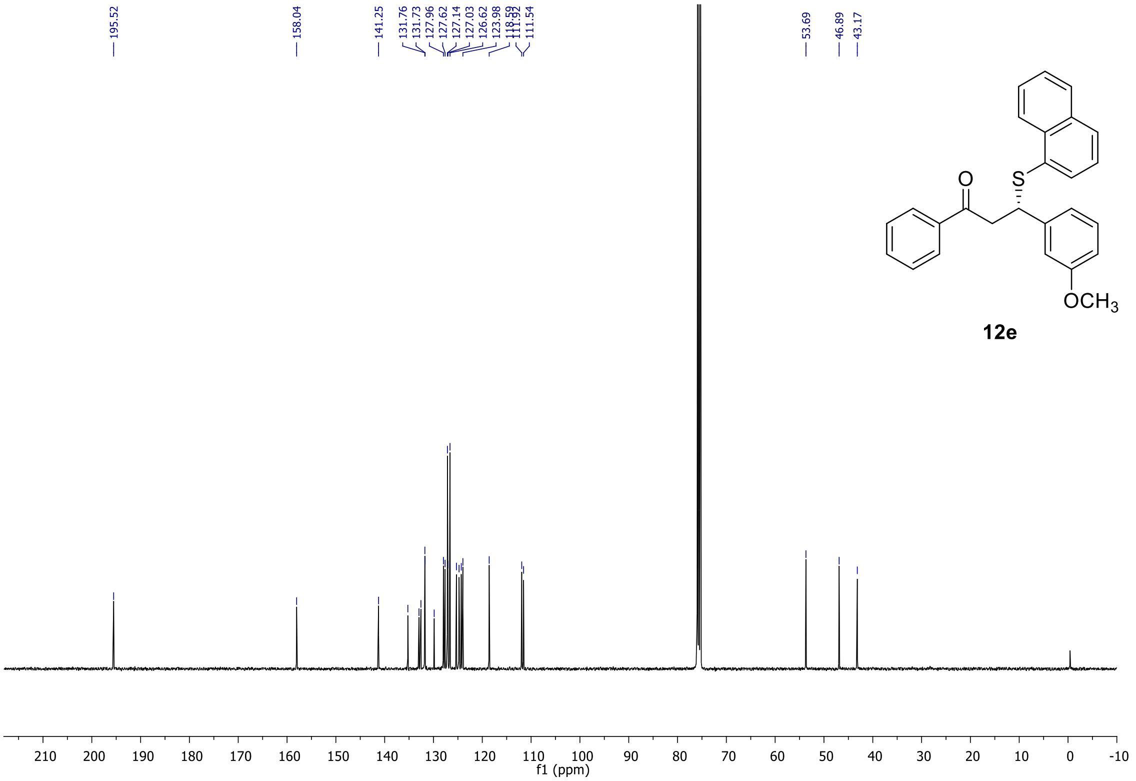
O
S
OCH3
12e
f1 (ppm)

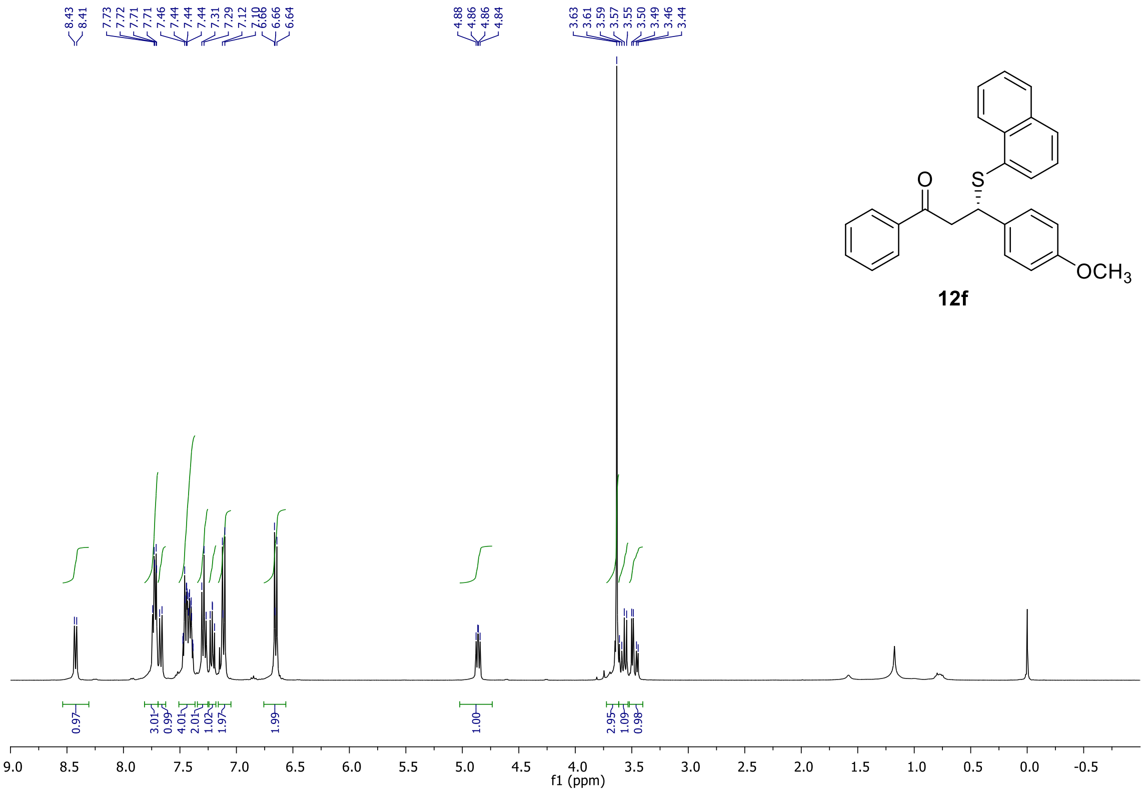

8.43
8.41
7.73
7.72
7.71
7.71
7.46
7.44
7.44
7.44
7.31
7.29
7.12
7.10
6.66
6.66
6.64
4.88
4.86
4.86
4.84
3.63
3.61
3.59
3.57
3.55
3.50
3.49
3.46
3.44
O
S
OCH3
12f
0.97
3.01
0.99
4.01
2.01
1.02
1.97
1.99
1.00
2.95
1.09
0.98
9.0 8.5 8.0 7.5 7.0 6.5 6.0 5.5 5.0 4.5 4.0 3.5 3.0 2.5 2.0 1.5 1.0 0.5 0.0 -0.5
f1 (ppm)

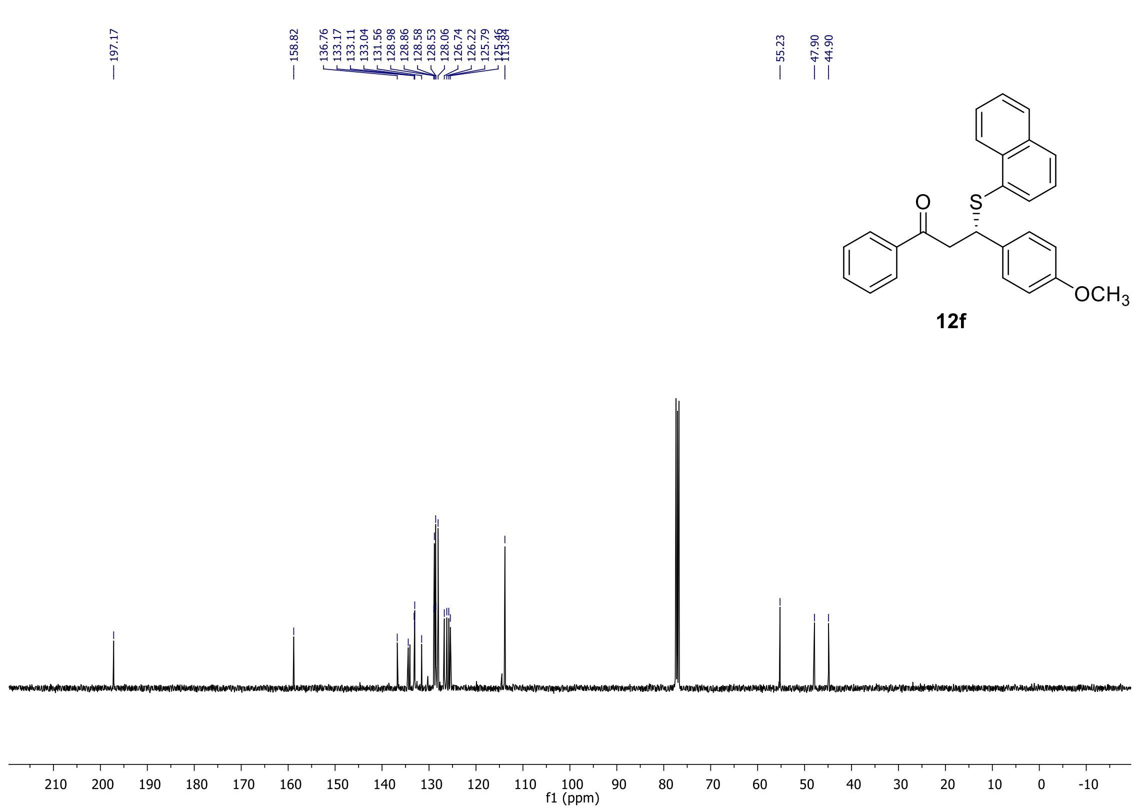

197.17
158.82
136.76
133.17
133.11
133.04
131.56
128.98
128.86
128.58
128.53
128.06
126.74
126.22
125.79
125.46
113.84
55.23
47.90
44.90
O
S
OCH3
12f
210 200 190 180 170 160 150 140 130 120 110 100 90 80 70 60 50 40 30 20 10 0 -10
f1 (ppm)

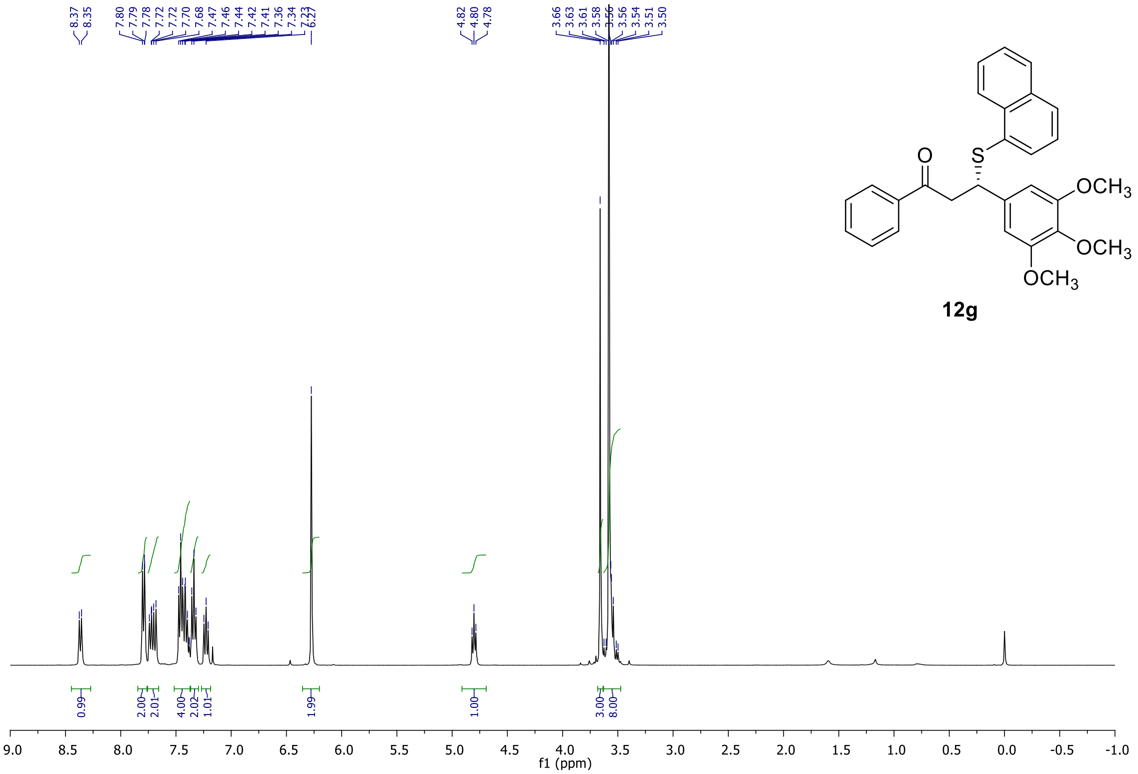
8.37
8.35
7.80
7.79
7.78
7.72
7.72
7.70
7.68
7.47
7.46
7.44
7.42
7.41
7.36
7.34
7.23
6.27
4.82
4.80
4.78
3.66
3.63
3.61
3.58
3.56
3.56
3.54
3.51
3.50
O
S
OCH3
OCH3
OCH3
12g
0.99
2.00
2.01
4.00
2.02
1.01
1.99
1.00
3.00
8.00
f1 (ppm)

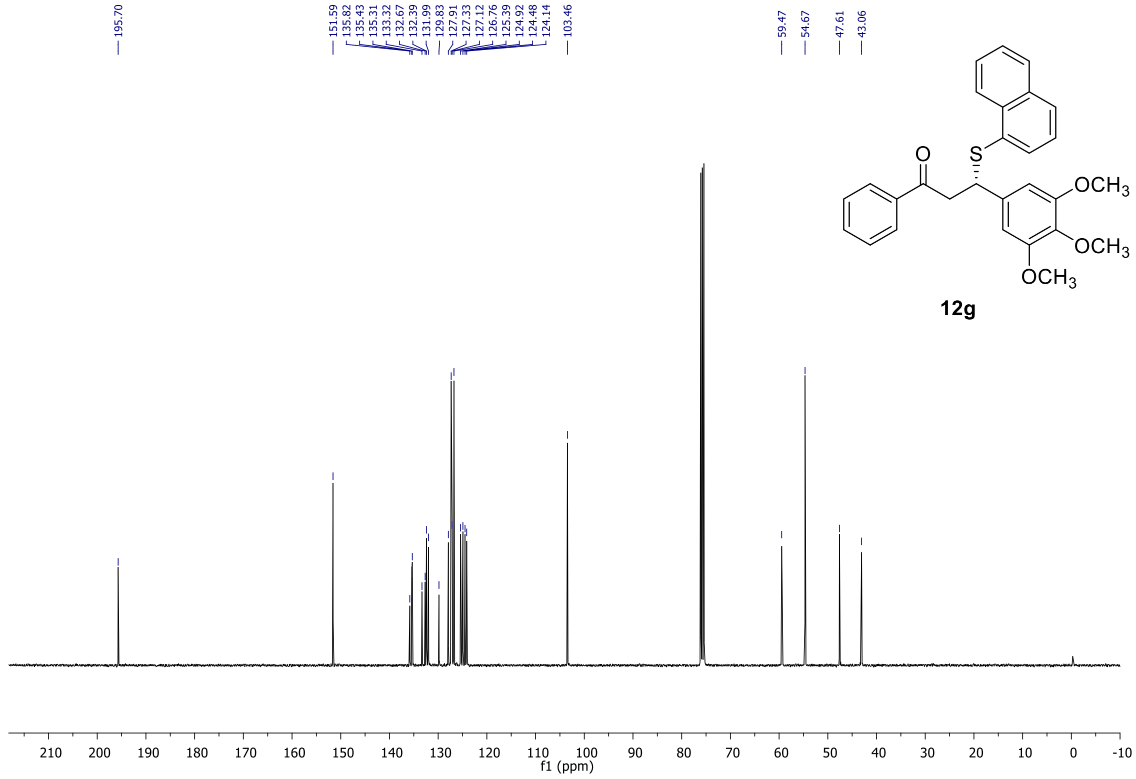
195.70
151.59
135.82
135.43
135.31
133.32
132.67
132.39
131.99
129.83
127.91
127.33
127.12
126.76
125.39
124.92
124.48
124.14
103.46
59.47
54.67
47.61
43.06
O
S
OCH3
OCH3
OCH3
12g
f1 (ppm)

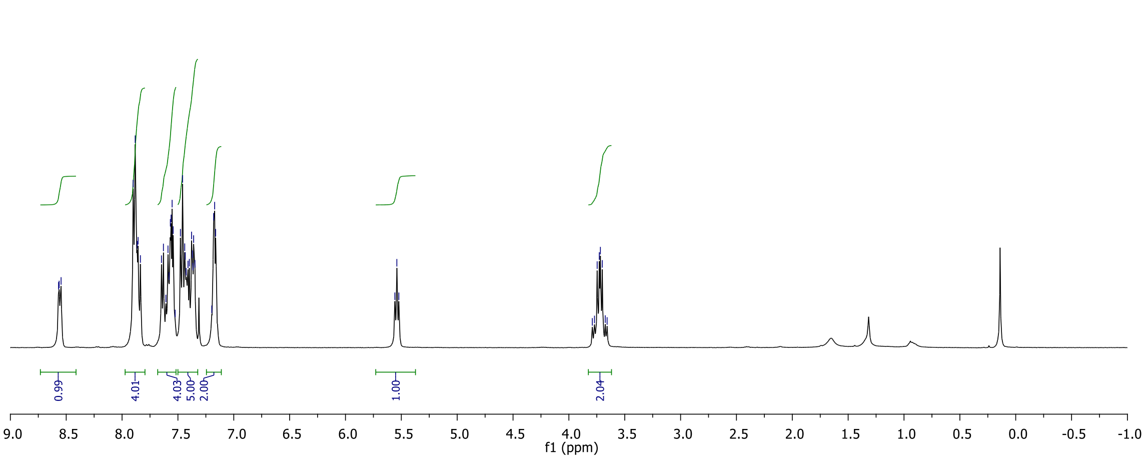

8.57
8.56
8.55
7.90
7.88
7.86
7.86
7.84
7.65
7.63
7.59
7.57
7.56
7.55
7.54
7.48
7.46
7.44
7.43
7.38
7.37
7.36
7.18
7.17
7.16
5.56
5.54
5.52
3.79
3.77
3.75
3.73
3.72
3.70
3.67
3.66
0.99
4.01
4.03
5.00
2.00
1.00
2.04
f1 (ppm)

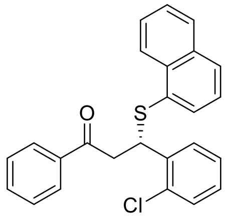

O
S
Cl

12h

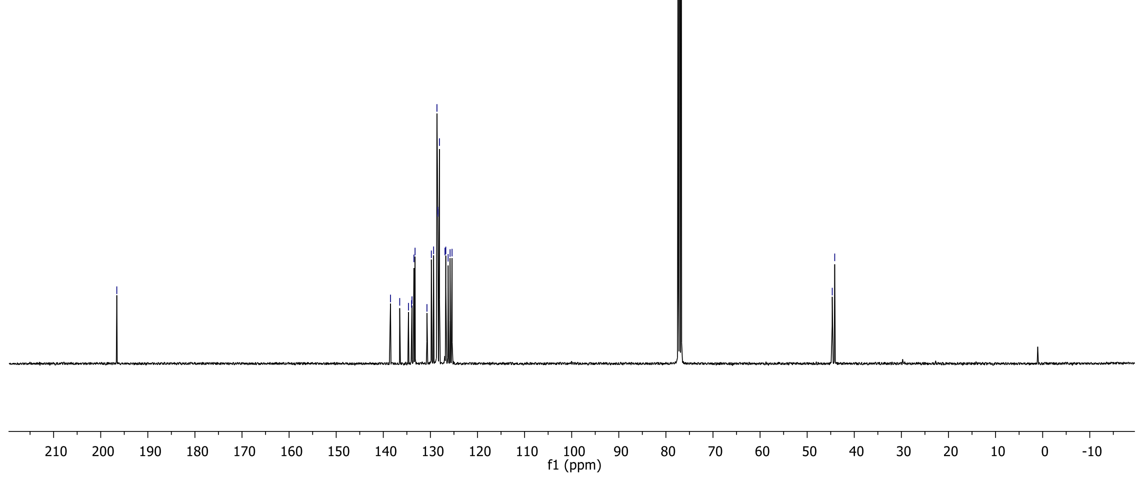

196.58
138.47
136.51
134.66
134.00
133.91
133.51
133.26
130.73
129.80
129.32
128.61
128.42
128.41
128.08
126.90
126.73
126.24
125.82
125.40
44.68
44.15
f1 (ppm)

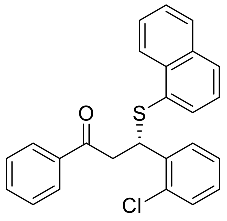

O
S
Cl

12h

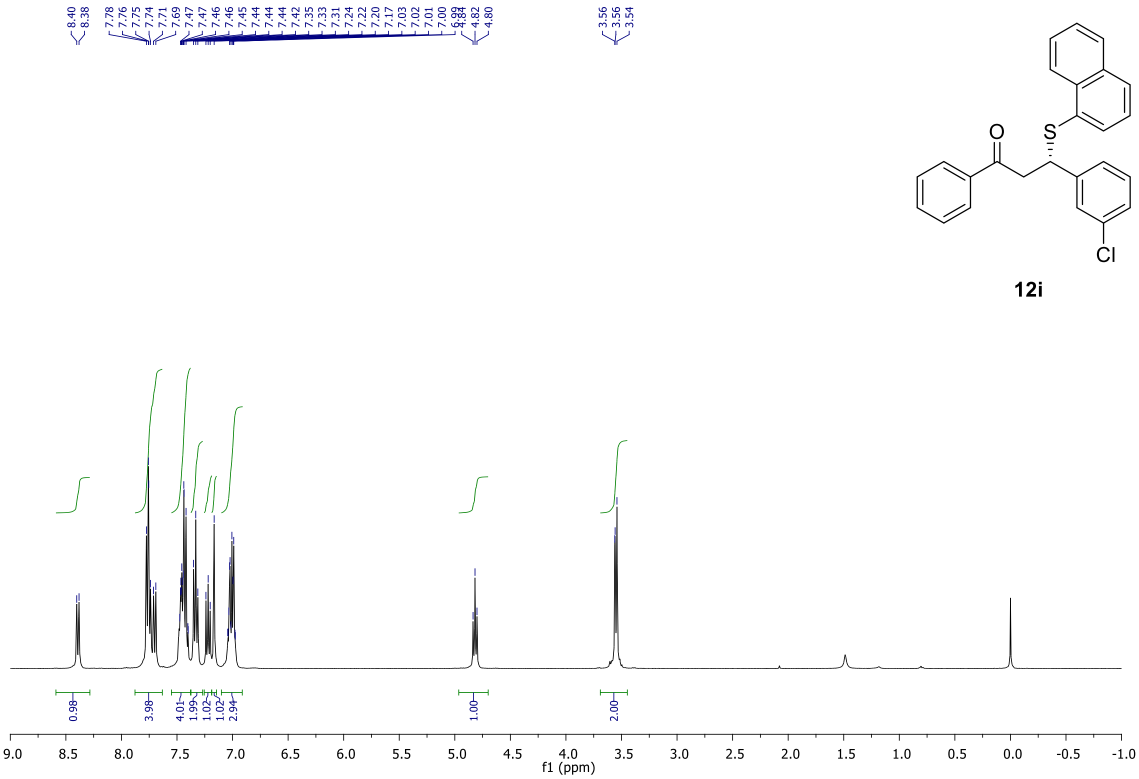
8.40
8.38
7.78
7.76
7.75
7.74
7.71
7.69
7.47
7.47
7.46
7.46
7.45
7.44
7.44
7.44
7.42
7.35
7.33
7.31
7.24
7.22
7.20
7.17
7.03
7.02
7.01
7.00
4.82
4.80
3.56
3.56
3.54
0.98
3.98
4.01
1.99
1.02
1.02
2.94
1.00
2.00
f1 (ppm)

12i

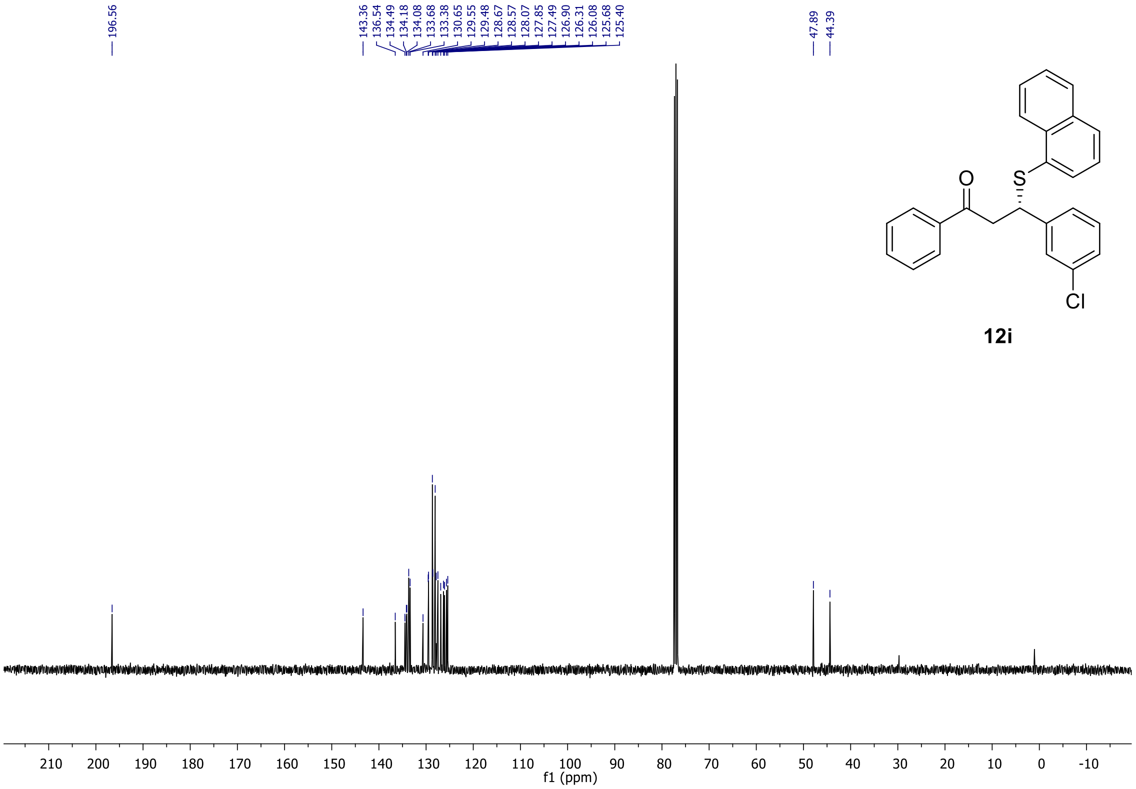
196.56
143.36
136.54
134.49
134.18
134.08
133.68
133.38
130.65
129.55
129.48
128.67
128.57
128.07
127.85
127.49
126.90
126.31
126.08
125.68
125.40
47.89
44.39
f1 (ppm)

12i

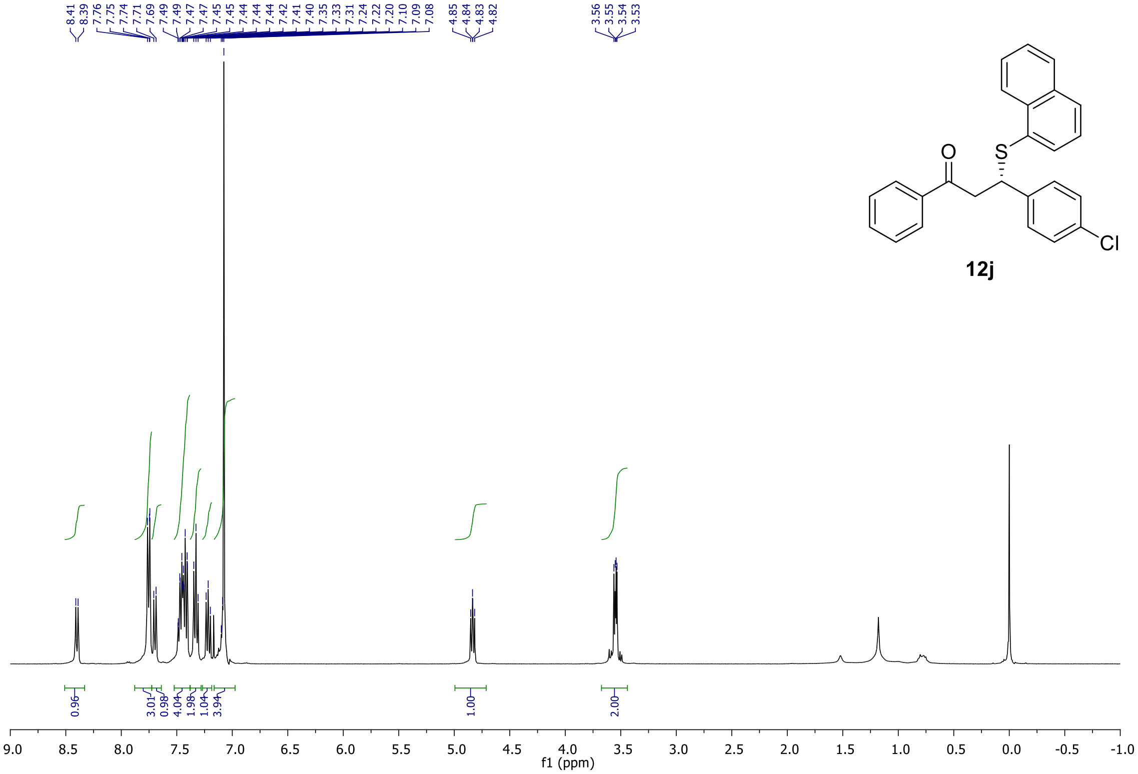
8.41
8.39
7.76
7.75
7.74
7.71
7.69
7.49
7.49
7.47
7.47
7.45
7.45
7.44
7.44
7.44
7.42
7.41
7.40
7.35
7.33
7.31
7.24
7.22
7.20
7.10
7.09
7.08
4.85
4.84
4.83
4.82
3.56
3.55
3.54
3.53
O
S
Cl
12j
0.96
3.01
0.98
4.04
1.98
1.04
3.94
1.00
2.00
f1 (ppm)

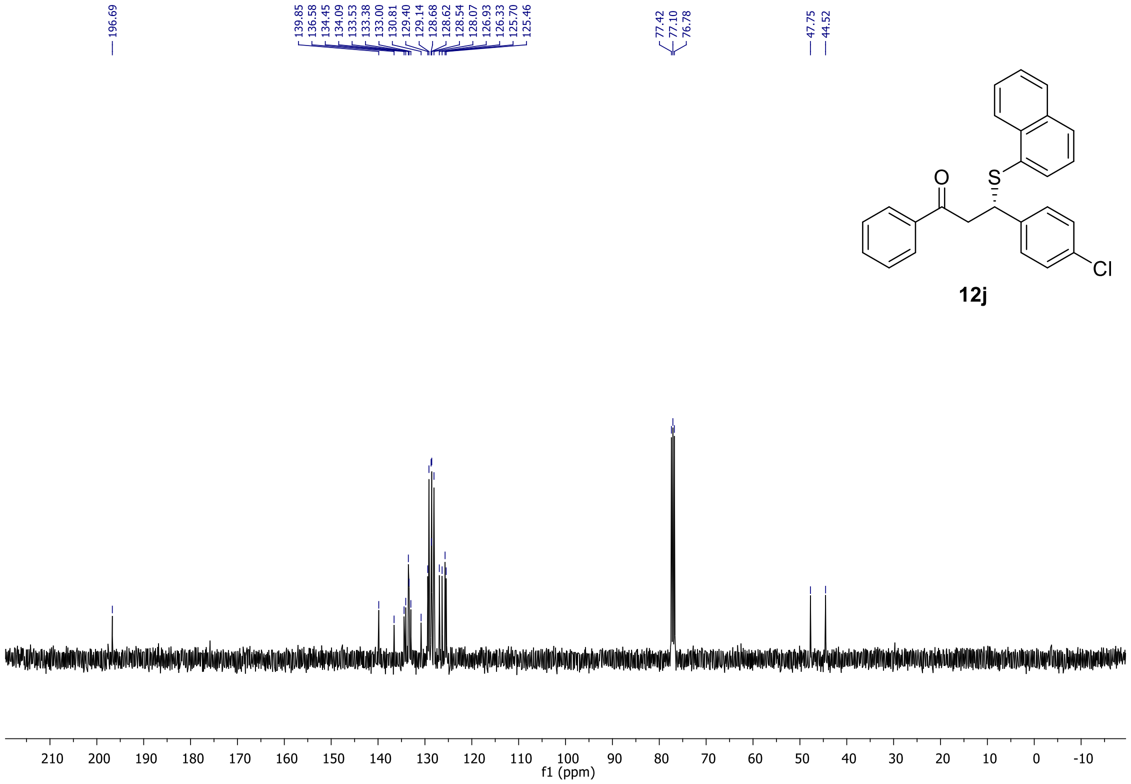
196.69
139.85
136.58
134.45
134.09
133.53
133.38
133.00
130.81
129.40
129.14
128.68
128.62
128.54
128.07
126.93
126.33
125.70
125.46
77.42
77.10
76.78
47.75
44.52
O
S
Cl
12j
f1 (ppm)

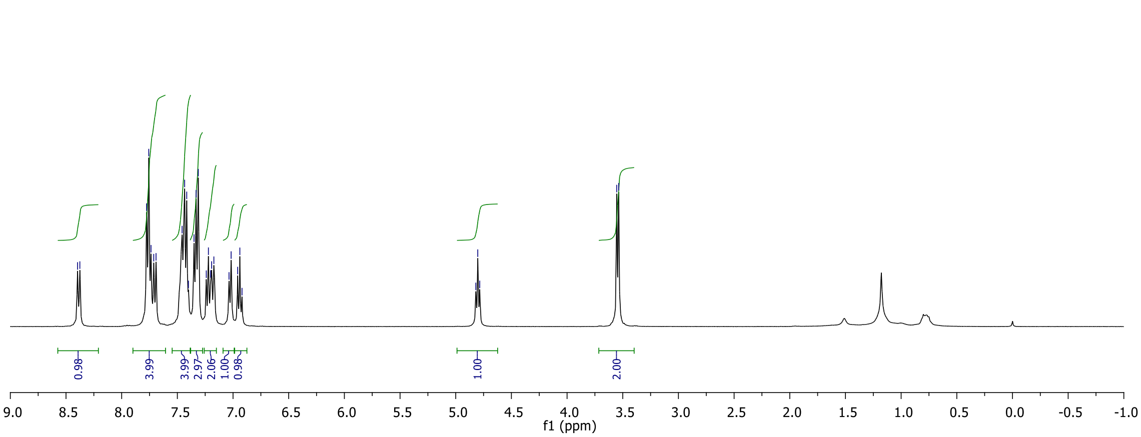

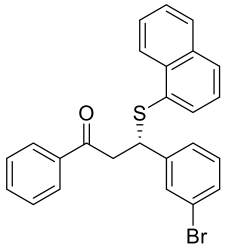

12k

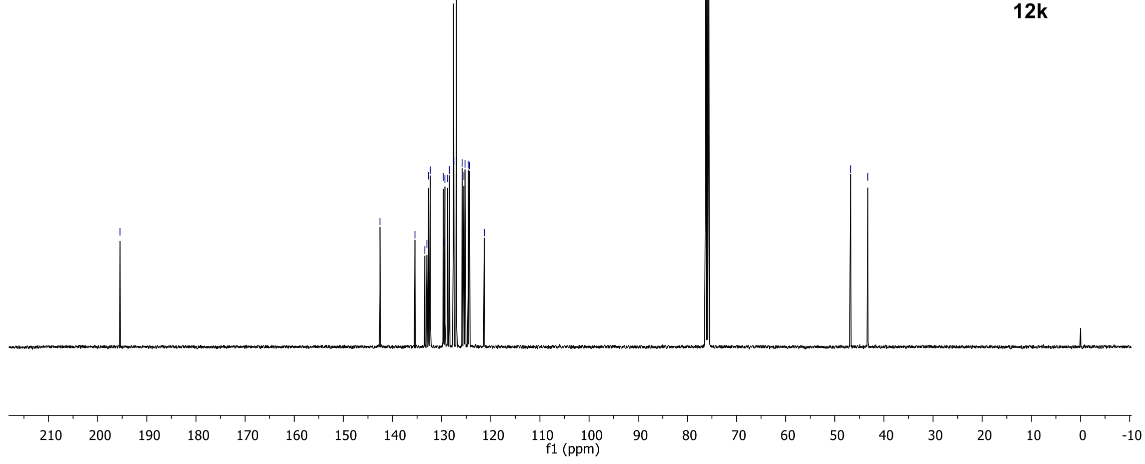

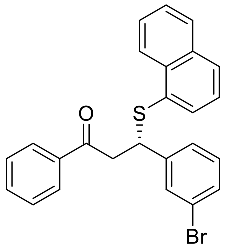

12k

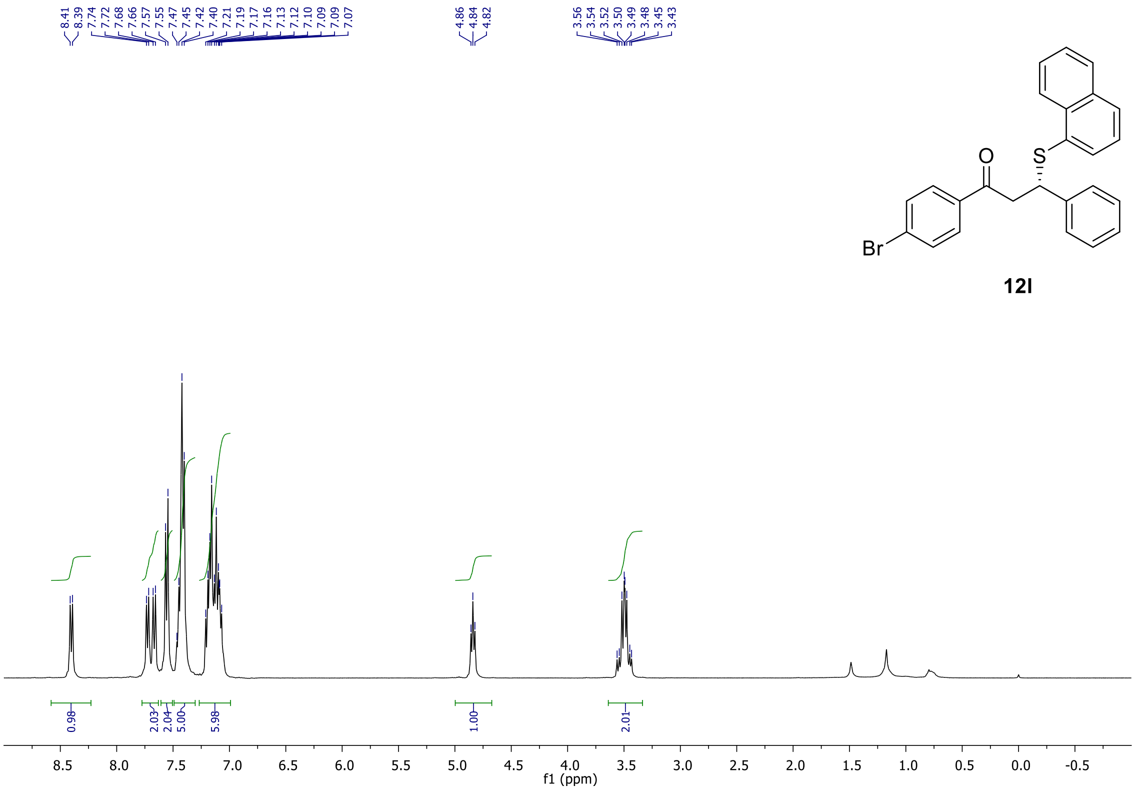
8.41
8.39
7.74
7.72
7.68
7.66
7.57
7.55
7.47
7.45
7.42
7.40
7.21
7.19
7.17
7.16
7.13
7.12
7.10
7.09
7.07
4.86
4.84
4.82
3.56
3.54
3.52
3.50
3.49
3.48
3.45
3.43
O
S
Br
12l
0.98
2.03
2.04
5.00
5.98
1.00
2.01
8.5 8.0 7.5 7.0 6.5 6.0 5.5 5.0 4.5 4.0 3.5 3.0 2.5 2.0 1.5 1.0 0.5 0.0 -0.5
f1 (ppm)

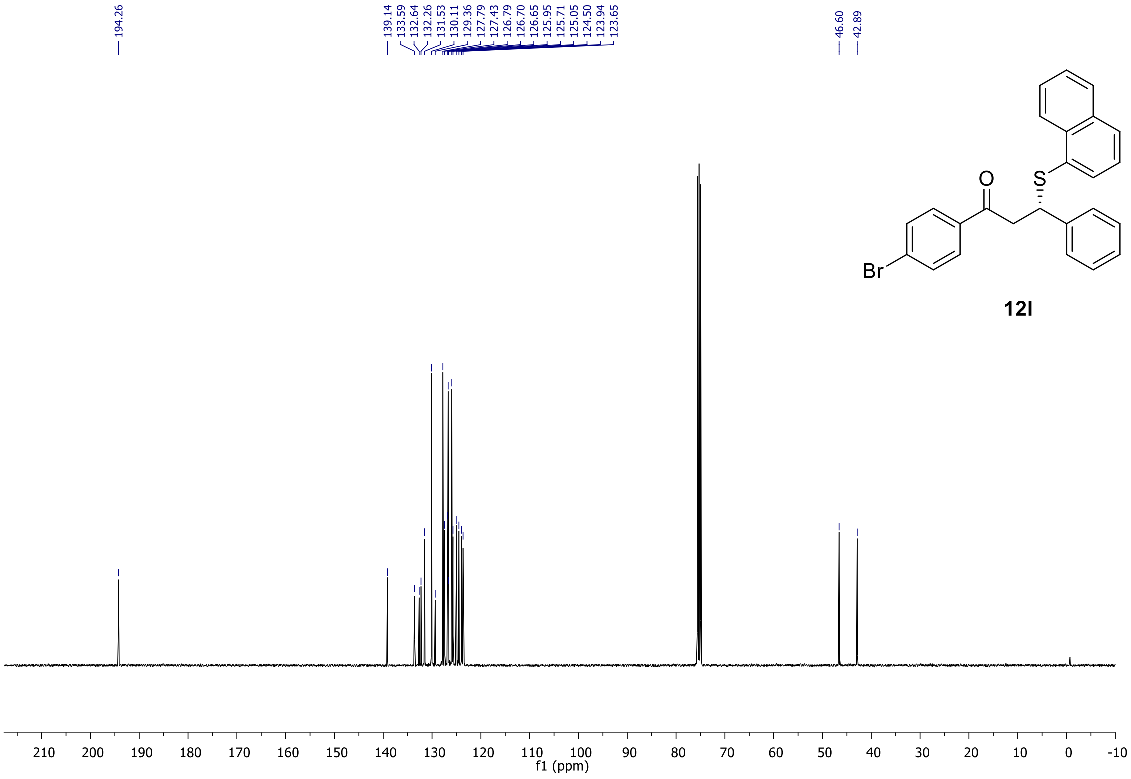
194.26
139.14
133.59
132.64
132.26
131.53
130.11
129.36
127.79
127.43
126.79
126.70
126.65
125.95
125.71
125.05
124.50
123.94
123.65
46.60
42.89
O
S
Br
12l
210 200 190 180 170 160 150 140 130 120 110 100 90 80 70 60 50 40 30 20 10 0 -10
f1 (ppm)

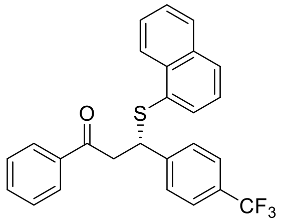

12m

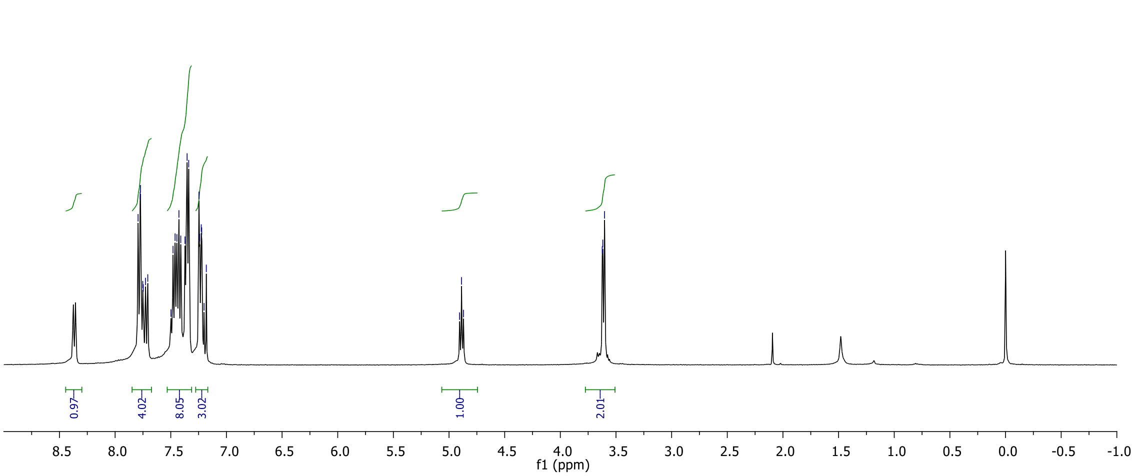
7.79
7.78
7.77
7.75
7.75
7.73
7.71
7.50
7.48
7.46
7.45
7.43
7.41
7.37
7.35
7.34
7.25
7.24
7.23
7.22
7.20
7.18
4.91
4.89
4.87
3.62
3.62
3.60
0.97
4.02
8.05
3.02
1.00
2.01
f1 (ppm)

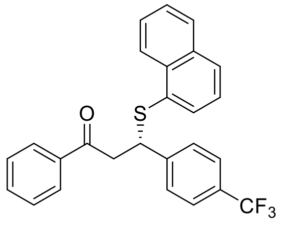

12m

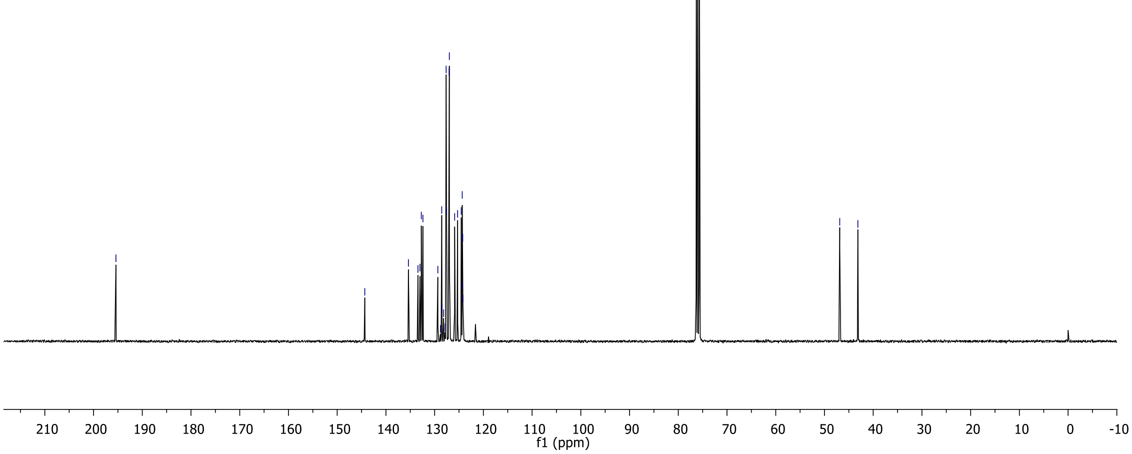
195.41
144.34
135.38
133.42
133.01
132.74
132.41
129.34
128.82
128.55
128.50
128.17
127.85
127.64
127.54
127.02
126.99
125.89
125.29
124.54
124.34
124.31
124.27
124.23
124.20
46.87
43.15
f1 (ppm)

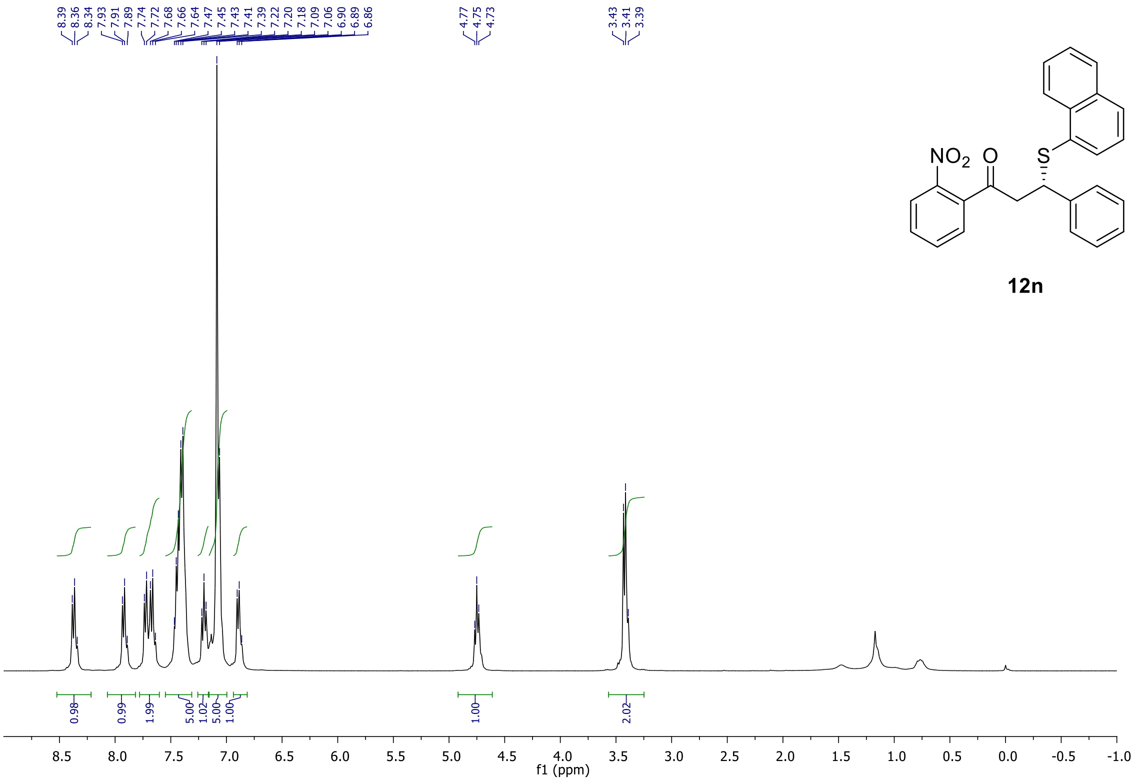
12n
f1 (ppm)

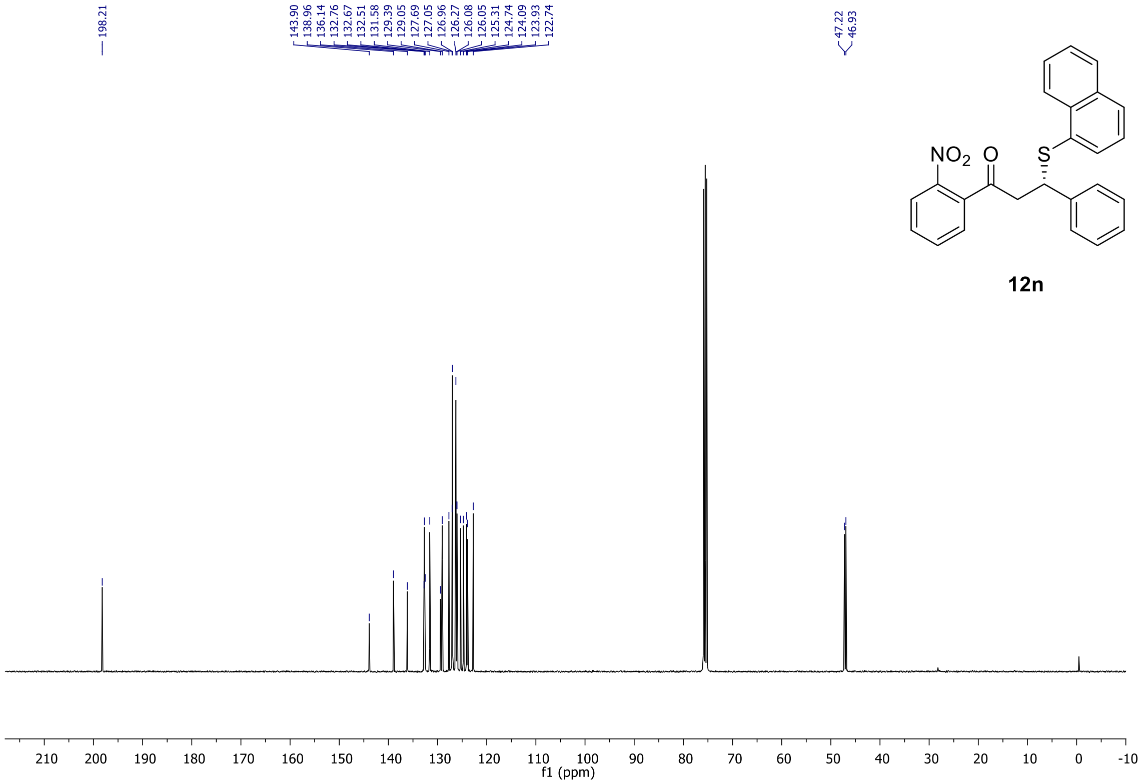
12n
f1 (ppm)

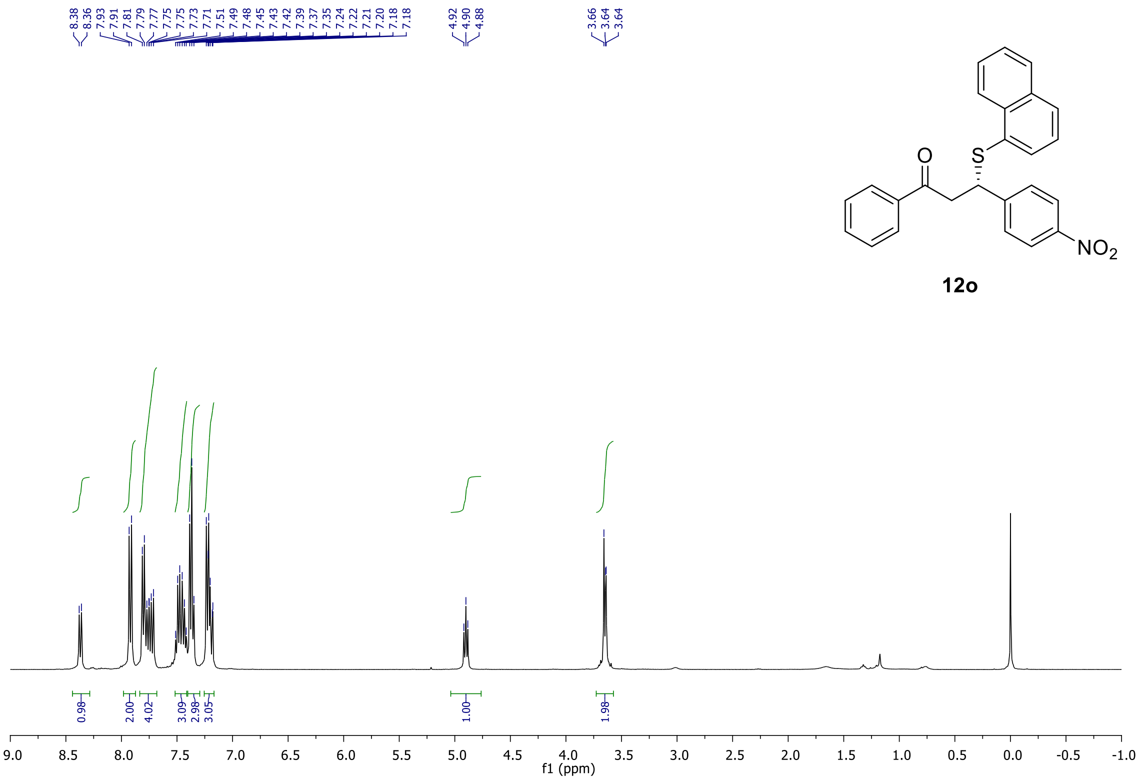
8.38
8.36
7.93
7.91
7.81
7.79
7.77
7.75
7.75
7.73
7.71
7.51
7.49
7.48
7.45
7.43
7.42
7.39
7.37
7.35
7.24
7.22
7.21
7.20
7.18
7.18
4.92
4.90
4.88
3.66
3.64
3.64
O
S
NO2
12o
0.98
2.00
4.02
3.09
2.98
3.05
1.00
1.98
9.0 8.5 8.0 7.5 7.0 6.5 6.0 5.5 5.0 4.5 4.0 3.5 3.0 2.5 2.0 1.5 1.0 0.5 0.0 -0.5 -1.0
f1 (ppm)

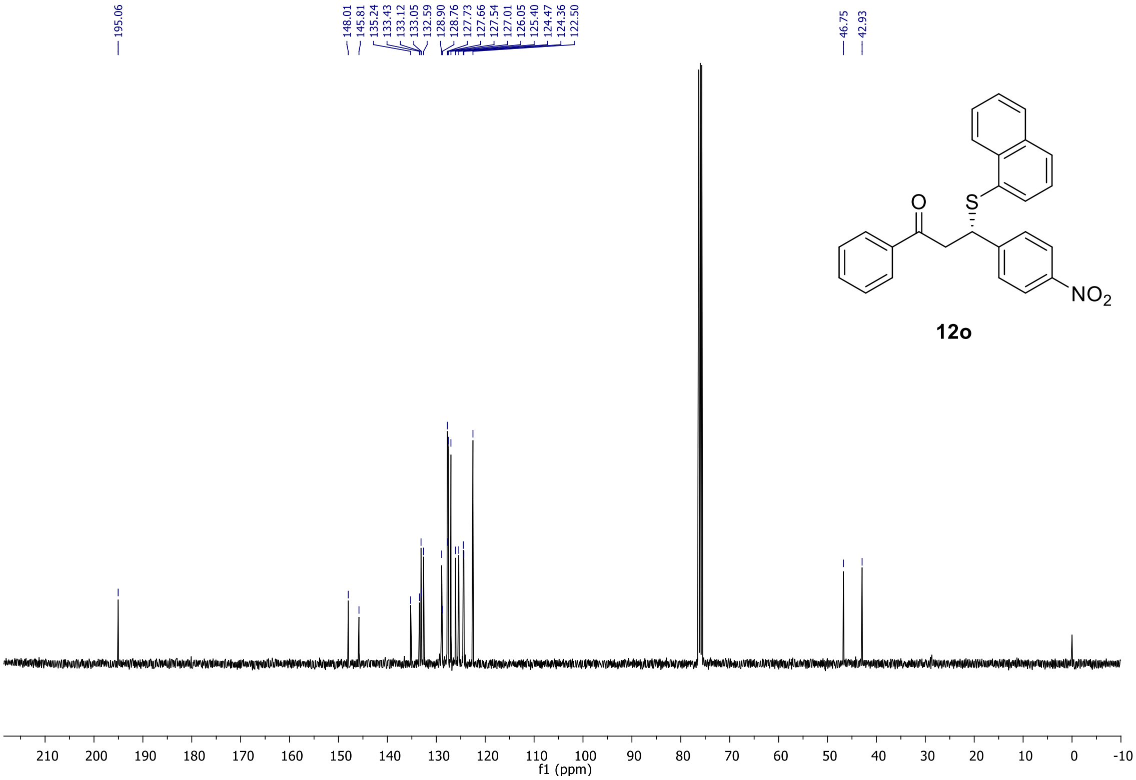
195.06
148.01
145.81
135.24
133.43
133.12
133.05
132.59
128.90
128.76
127.73
127.66
127.54
127.01
126.05
125.40
124.47
124.36
122.50
46.75
42.93
O
S
NO2
12o
210 200 190 180 170 160 150 140 130 120 110 100 90 80 70 60 50 40 30 20 10 0 -10
f1 (ppm)

V. HPLC chromatograms of products 12a–o

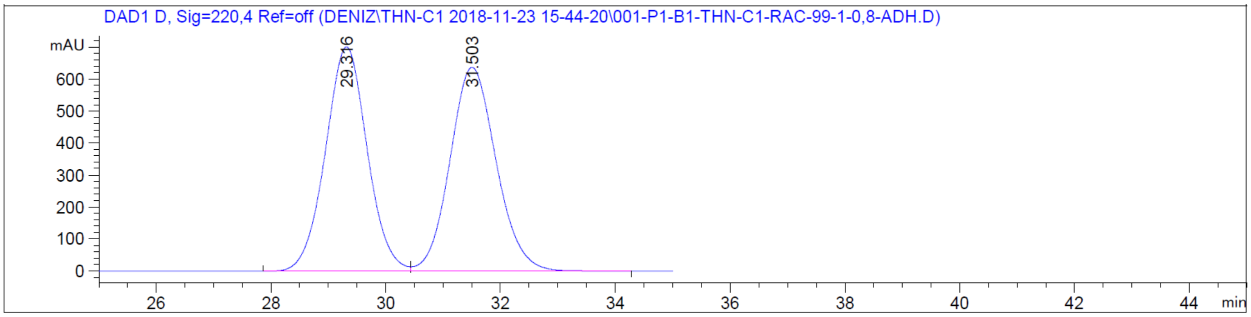

Signal 4: DAD1 D, Sig=220,4 Ref=off

Peak #	RetTime [min]	Type	Width [min]	Area [mAU*s]	Height [mAU]	Area %
1	29.316	BV	0.7698	3.55025e4	701.65533	49.8950
2	31.503	VB	0.8501	3.56520e4	637.84937	50.1050
Totals :				7.11545e4	1339.50470	

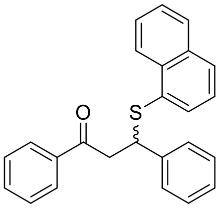

***(rac)*-12a**

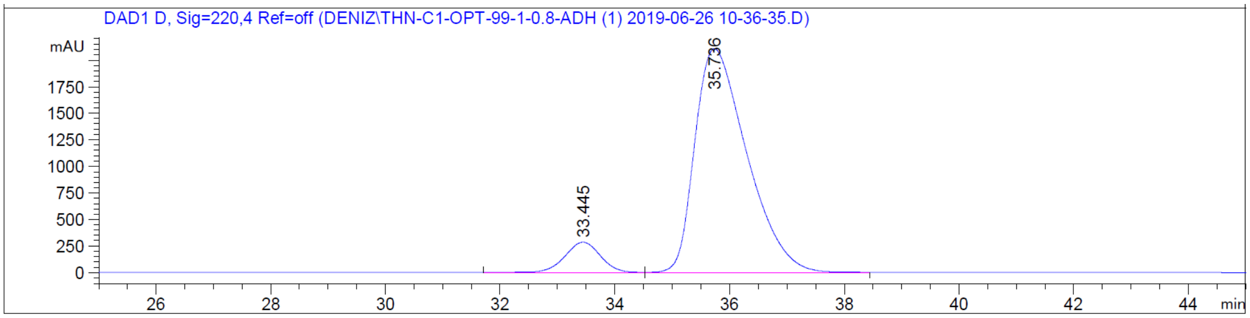

Signal 4: DAD1 D, Sig=220,4 Ref=off

Peak #	RetTime [min]	Type	Width [min]	Area [mAU*s]	Height [mAU]	Area %
1	33.445	BB	0.6536	1.21139e4	283.73450	8.3928
2	35.736	BB	0.8428	1.32223e5	2105.19702	91.6072
Totals :				1.44337e5	2388.93152	

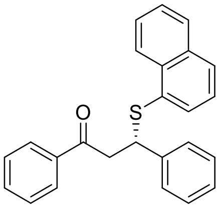

***(S)*-12a**

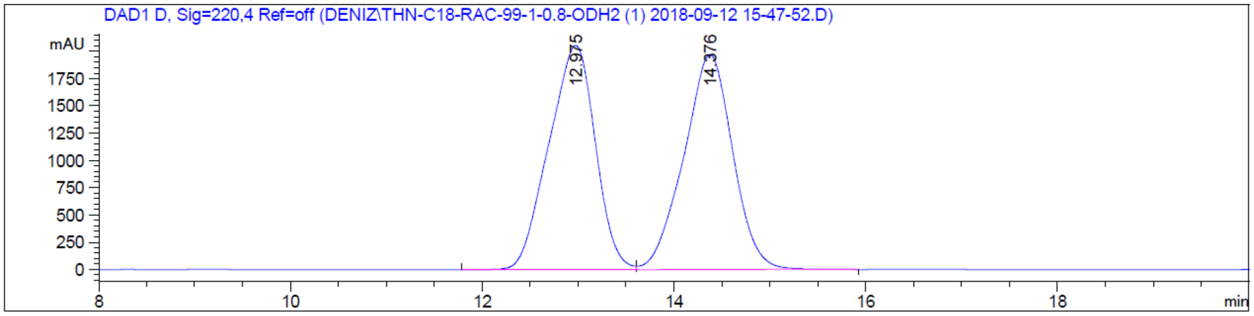

Signal 4: DAD1 D, Sig=220,4 Ref=off

Peak #	RetTime [min]	Type	Width [min]	Area [mAU*s]	Height [mAU]	Area %
1	12.975	BV	0.4956	6.92892e4	2056.39526	49.7526
2	14.376	VB	0.5261	6.99784e4	1971.32837	50.2474
Totals :				1.39268e5	4027.72363	

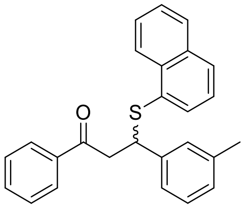

***(rac)*-12b**

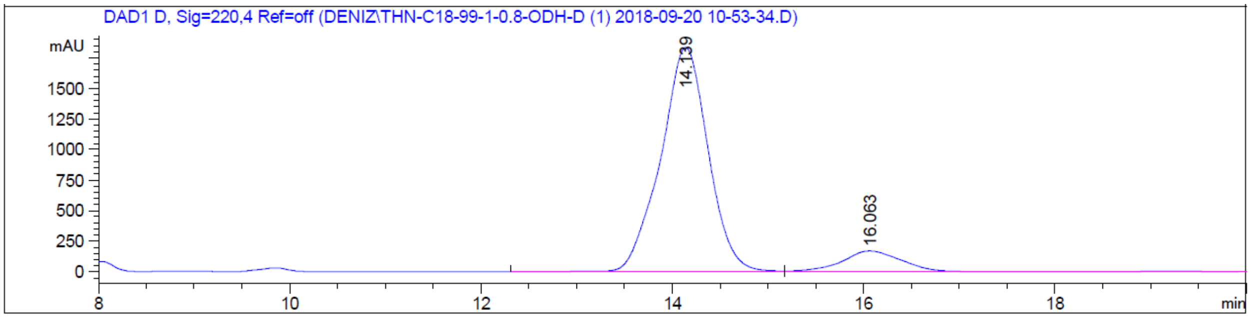

Signal 4: DAD1 D, Sig=220,4 Ref=off

Peak #	RetTime [min]	Type	Width [min]	Area [mAU*s]	Height [mAU]	Area %
1	14.139	BV	0.5148	6.34026e4	1836.55701	89.1188
2	16.063	VV R	0.6765	7741.29199	170.84424	10.8812
Totals :				7.11439e4	2007.40125	

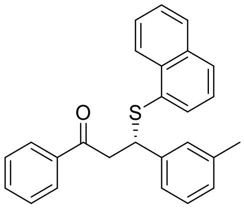

***(S)*-12b**

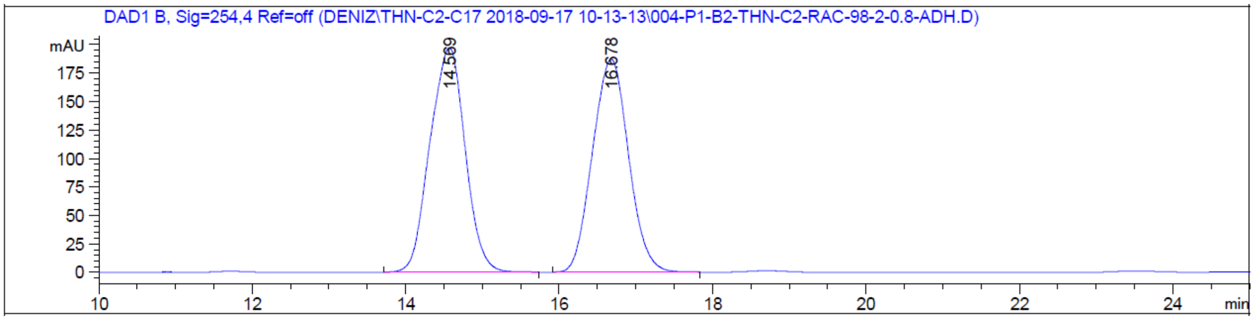

Signal 2: DAD1 B, Sig=254,4 Ref=off

Peak #	RetTime [min]	Type	Width [min]	Area [mAU*s]	Height [mAU]	Area %
1	14.569	BB	0.5150	6273.52637	197.29555	50.6657
2	16.678	BB	0.5030	6108.66602	187.07419	49.3343

Totals : 1.23822e4 384.36974

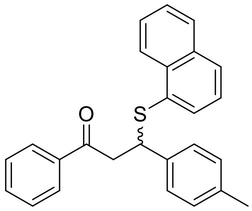

***(rac)*-12c**

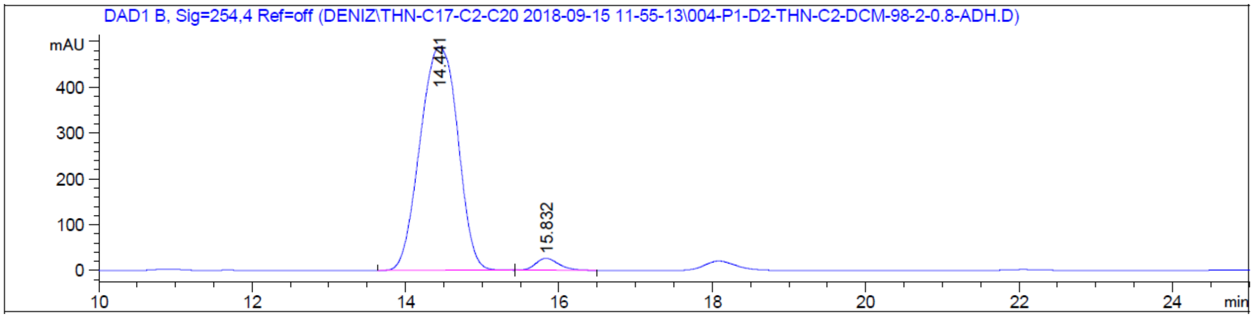

Signal 2: DAD1 B, Sig=254,4 Ref=off

Peak #	RetTime [min]	Type	Width [min]	Area [mAU*s]	Height [mAU]	Area %
1	14.441	BB	0.5500	1.66455e4	488.53439	96.8666
2	15.832	BB	0.3112	538.44464	26.15174	3.1334

Totals : 1.71839e4 514.68613

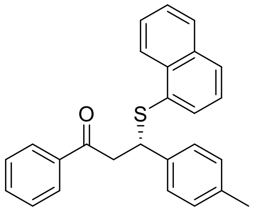

***(S)*-12c**

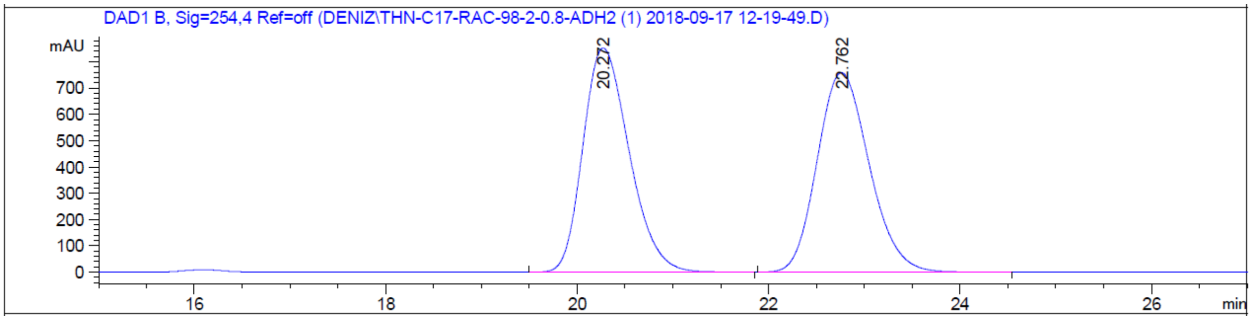

Signal 2: DAD1 B, Sig=254,4 Ref=off

Peak #	RetTime [min]	Type	Width [min]	Area [mAU*s]	Height [mAU]	Area %
1	20.272	BB	0.5211	2.85410e4	852.49890	49.9960
2	22.762	BB	0.5825	2.85456e4	758.69543	50.0040

Totals : 5.70866e4 1611.19434

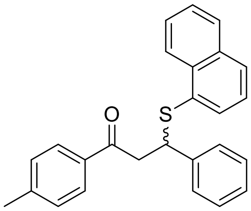

***(rac)*-12d**

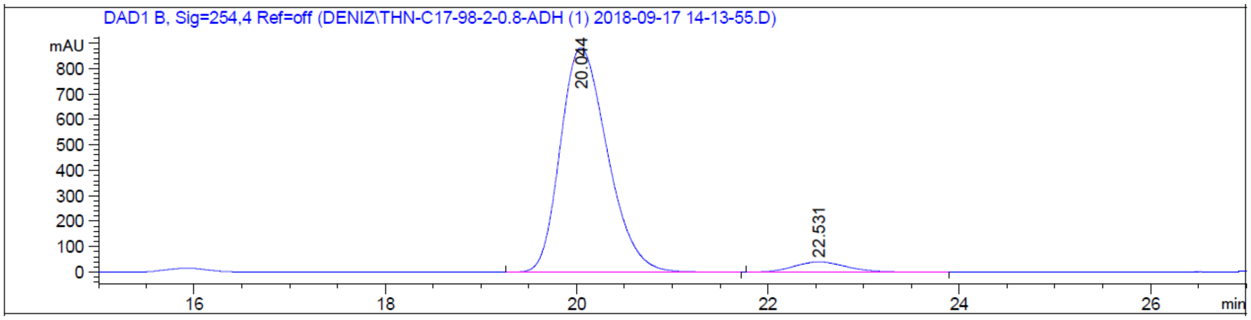

Signal 2: DAD1 B, Sig=254,4 Ref=off

Peak #	RetTime [min]	Type	Width [min]	Area [mAU*s]	Height [mAU]	Area %
1	20.044	BB	0.5308	3.00184e4	881.47162	95.3108
2	22.531	BB	0.5002	1476.88599	39.27663	4.6892

Totals : 3.14953e4 920.74825

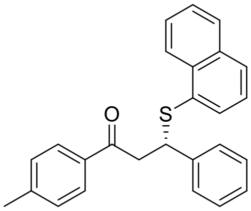

***(S)*-12d**

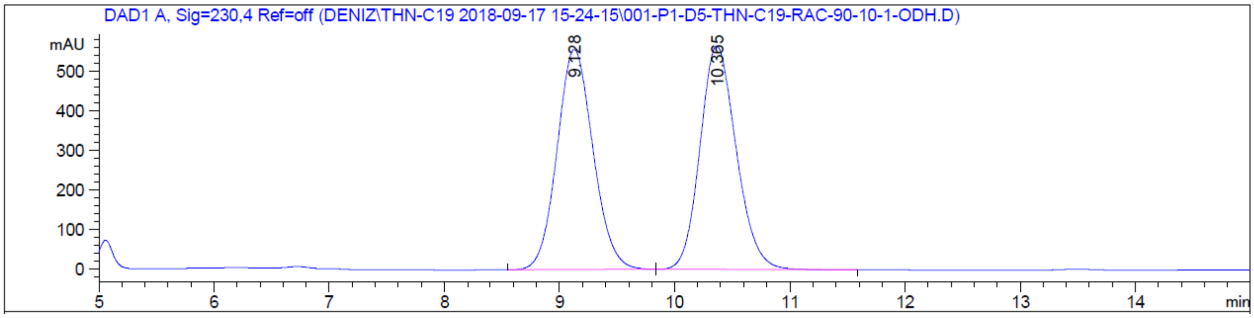

Signal 1: DAD1 A, Sig=230,4 Ref=off

Peak #	RetTime [min]	Type	Width [min]	Area [mAU*s]	Height [mAU]	Area %
1	9.128	BB	0.3359	1.21252e4	557.19781	49.4275
2	10.365	BB	0.3386	1.24061e4	566.28217	50.5725

Totals : 2.45312e4 1123.47998

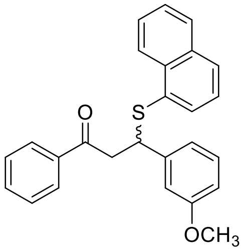

***(rac)*-12e**

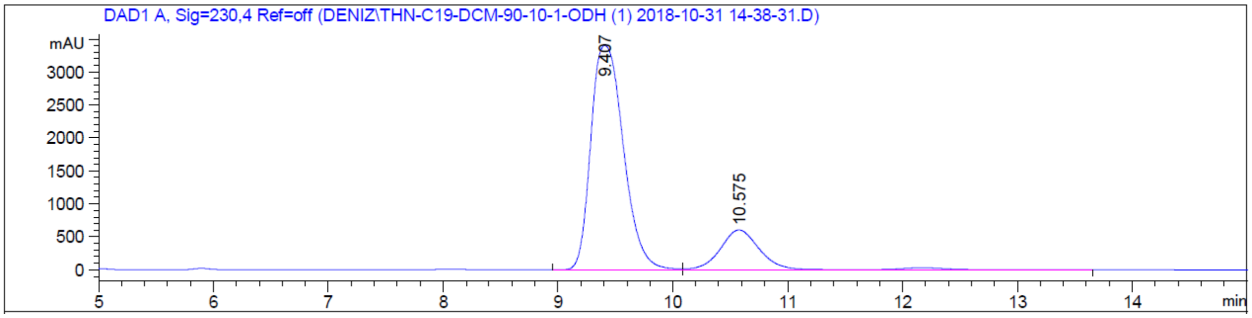

Signal 1: DAD1 A, Sig=230,4 Ref=off

Peak #	RetTime [min]	Type	Width [min]	Area [mAU*s]	Height [mAU]	Area %
1	9.407	BV	0.3099	6.70355e4	3402.12036	81.5014
2	10.575	VV R	0.3561	1.52152e4	604.47833	18.4986

Totals : 8.22507e4 4006.59869

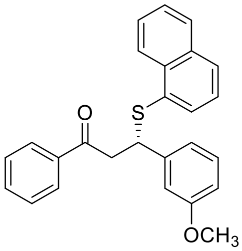

***(S)*-12e**

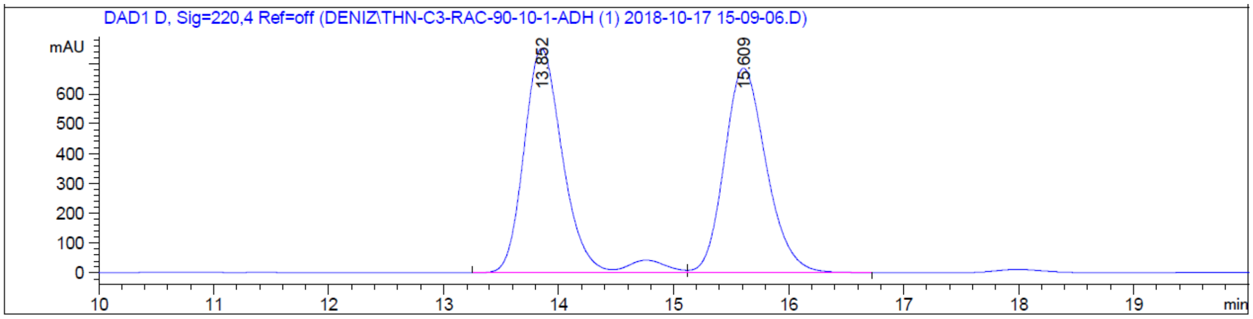

Signal 4: DAD1 D, Sig=220,4 Ref=off

Peak #	RetTime [min]	Type	Width [min]	Area [mAU*s]	Height [mAU]	Area %
1	13.852	BV R	0.3547	1.82112e4	752.14575	51.2721
2	15.609	VB	0.3882	1.73076e4	685.24670	48.7279

Totals : 3.55188e4 1437.39246

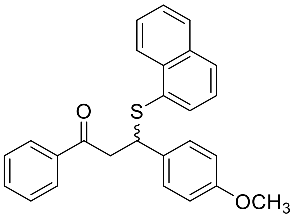

***(rac)*-12f**

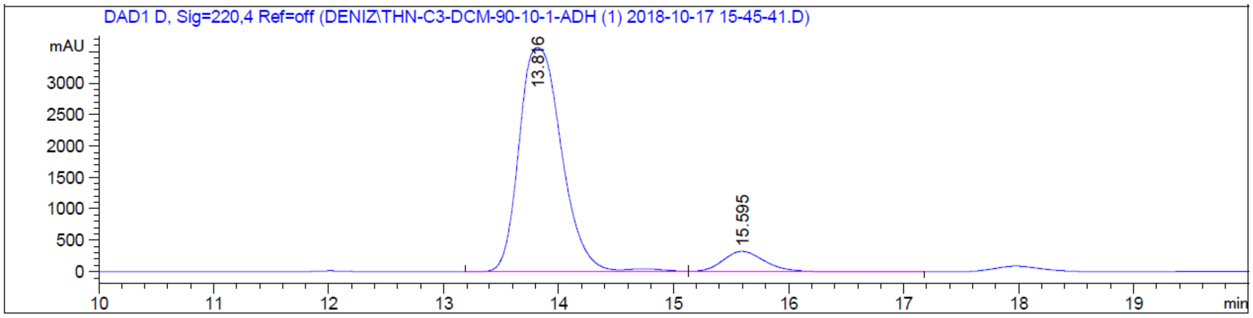

Signal 4: DAD1 D, Sig=220,4 Ref=off

Peak #	RetTime [min]	Type	Width [min]	Area [mAU*s]	Height [mAU]	Area %
1	13.816	BV R	0.2993	9.17834e4	3569.05933	91.7712
2	15.595	VB	0.3873	8229.84668	324.03961	8.2288

Totals : 1.00013e5 3893.09894

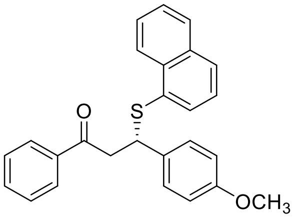

***(S)*-12f**

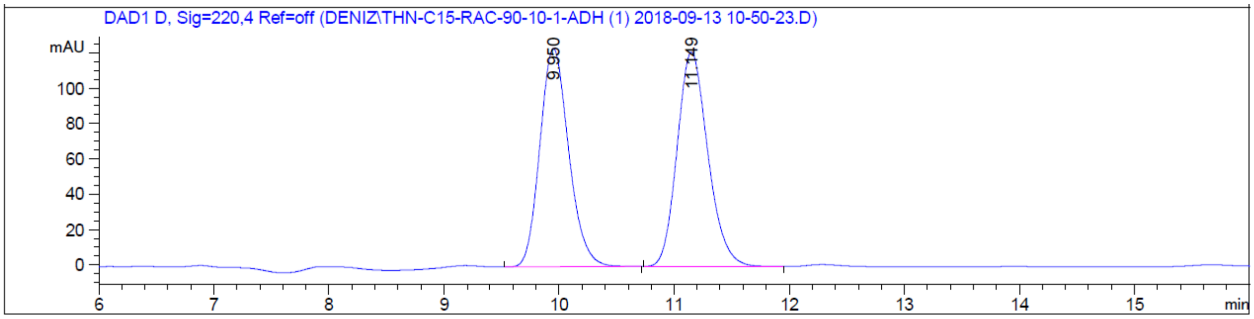

Signal 4: DAD1 D, Sig=220,4 Ref=off

Peak #	RetTime [min]	Type	Width [min]	Area [mAU*s]	Height [mAU]	Area %
1	9.950	BB	0.2683	2156.46191	123.72632	48.8890
2	11.149	BB	0.2862	2254.47583	121.39420	51.1110
Totals :				4410.93774	245.12052	

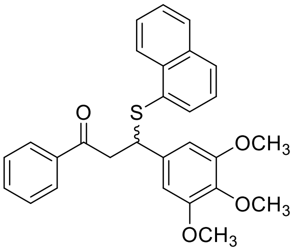

***(rac)*-12g**

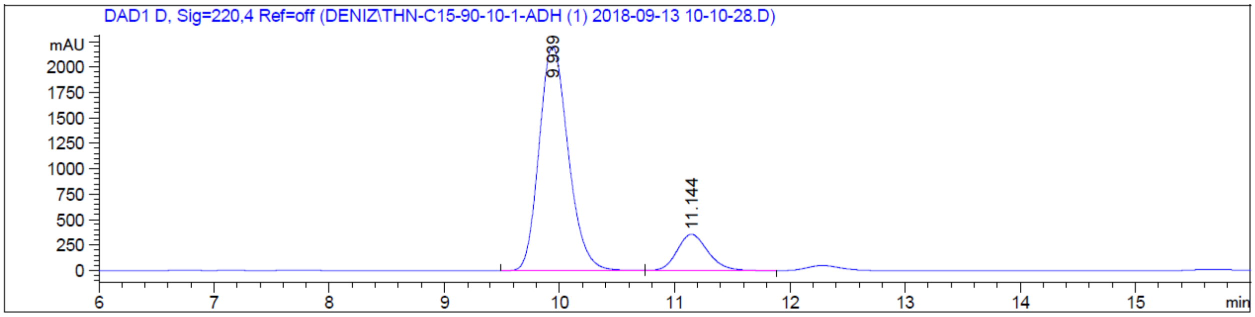

Signal 4: DAD1 D, Sig=220,4 Ref=off

Peak #	RetTime [min]	Type	Width [min]	Area [mAU*s]	Height [mAU]	Area %
1	9.939	BV	0.2725	3.87236e4	2203.32349	85.4165
2	11.144	VB	0.2856	6611.40771	356.05481	14.5835
Totals :				4.53350e4	2559.37830	

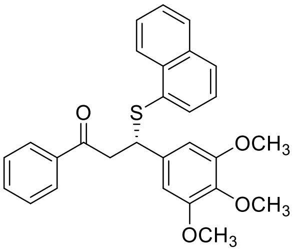

***(S)*-12g**

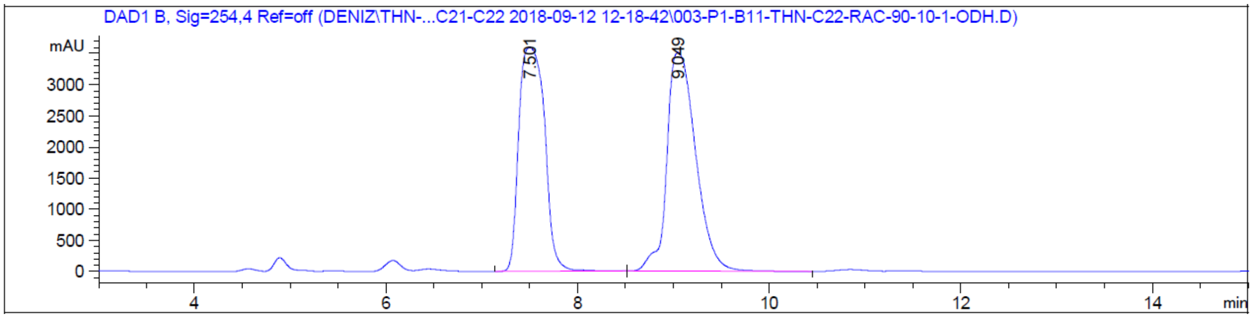

Signal 2: DAD1 B, Sig=254,4 Ref=off

Peak #	RetTime [min]	Type	Width [min]	Area [mAU*s]	Height [mAU]	Area %
1	7.501	BB	0.3103	6.72355e4	3593.60767	47.7895
2	9.049	BB	0.3240	7.34555e4	3512.53784	52.2105

Totals : 1.40691e5 7106.14551

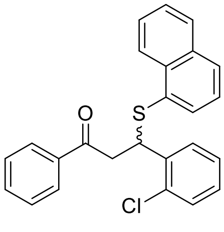

***(rac)*-12h**

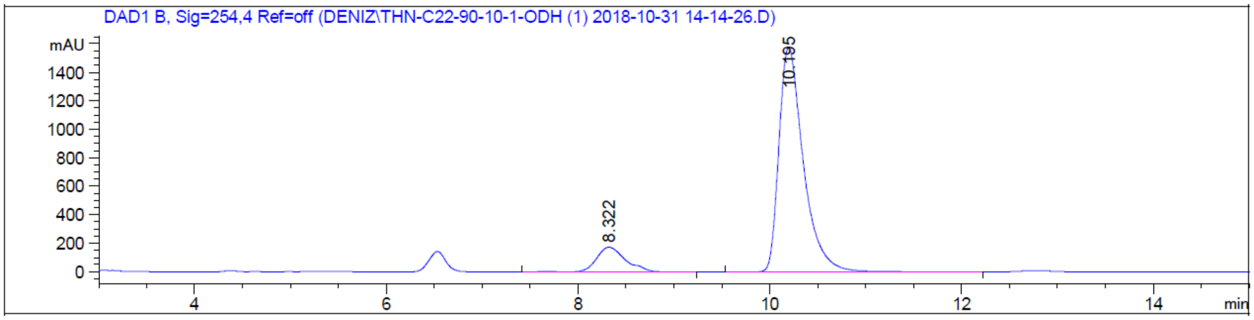

Signal 2: DAD1 B, Sig=254,4 Ref=off

Peak #	RetTime [min]	Type	Width [min]	Area [mAU*s]	Height [mAU]	Area %
1	8.322	VB R	0.3137	3624.86670	172.64355	11.5050
2	10.195	BB	0.2682	2.78821e4	1577.27319	88.4950

Totals : 3.15069e4 1749.91675

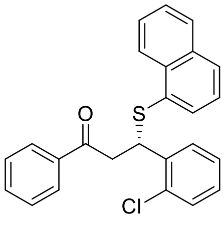

***(S)*-12h**

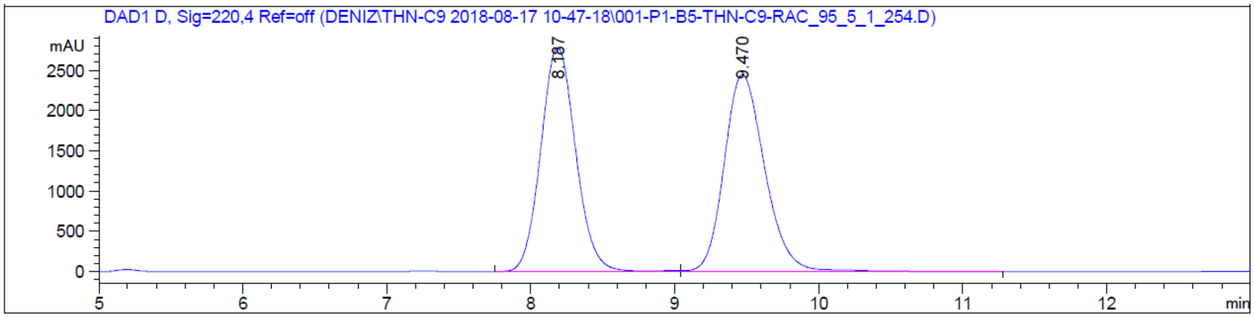

Signal 4: DAD1 D, Sig=220,4 Ref=off

Peak #	RetTime [min]	Type	Width [min]	Area [mAU*s]	Height [mAU]	Area %
1	8.187	BV R	0.2594	4.69352e4	2785.85107	49.4771
2	9.470	VB	0.3026	4.79274e4	2446.08301	50.5229

Totals : 9.48626e4 5231.93408

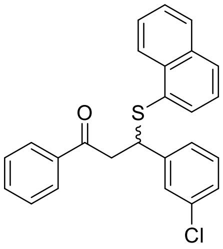

***(rac)*-12i**

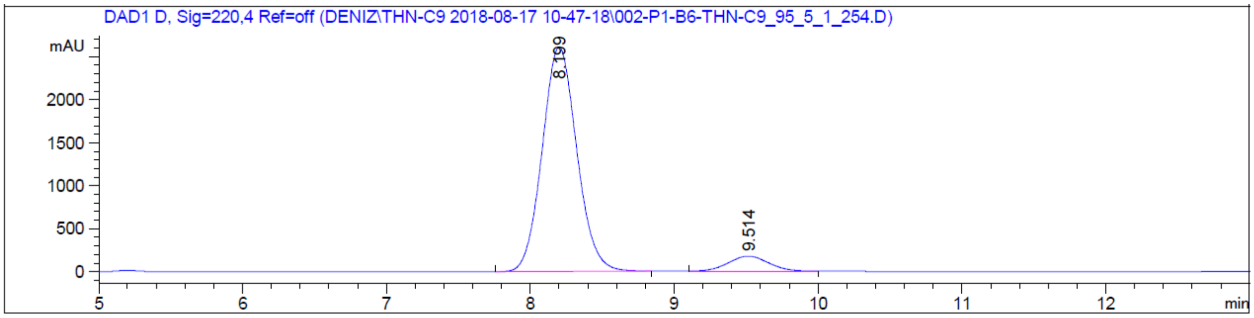

Signal 4: DAD1 D, Sig=220,4 Ref=off

Peak #	RetTime [min]	Type	Width [min]	Area [mAU*s]	Height [mAU]	Area %
1	8.199	BB	0.2573	4.35814e4	2609.86353	92.7353
2	9.514	BB	0.3016	3414.07690	175.42325	7.2647

Totals : 4.69955e4 2785.28677

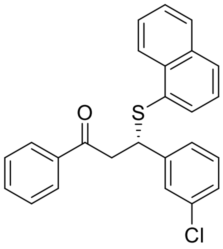

***(S)*-12i**

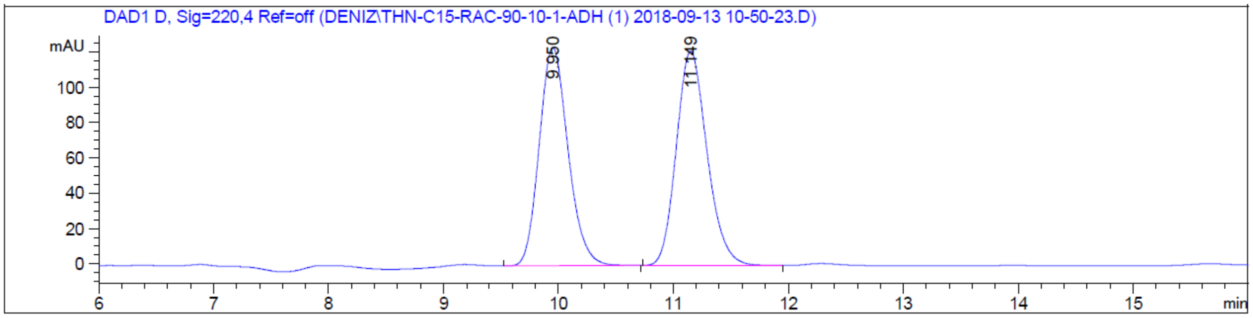

Signal 4: DAD1 D, Sig=220,4 Ref=off

Peak #	RetTime [min]	Type	Width [min]	Area [mAU*s]	Height [mAU]	Area %
1	9.950	BB	0.2683	2156.46191	123.72632	48.8890
2	11.149	BB	0.2862	2254.47583	121.39420	51.1110

Totals : 4410.93774 245.12052

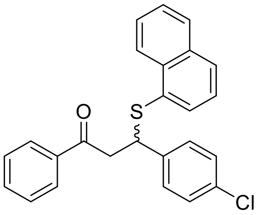

***(rac)*-12j**

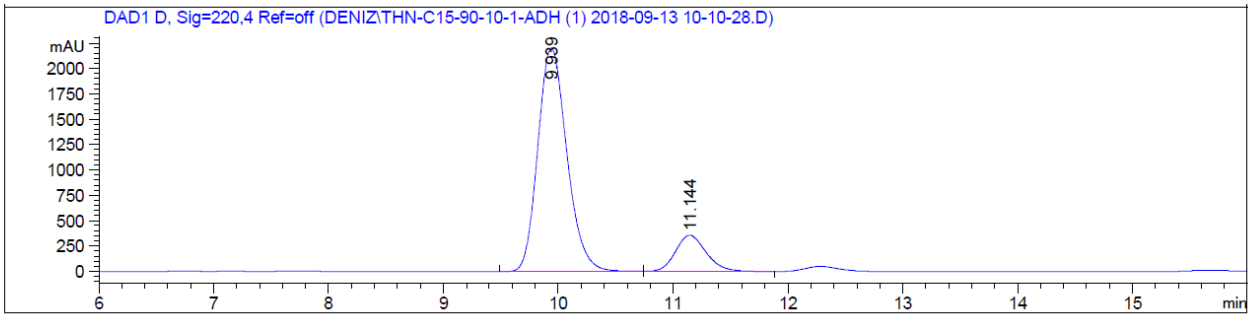

Signal 4: DAD1 D, Sig=220,4 Ref=off

Peak #	RetTime [min]	Type	Width [min]	Area [mAU*s]	Height [mAU]	Area %
1	9.939	BV	0.2725	3.87236e4	2203.32349	85.4165
2	11.144	VB	0.2856	6611.40771	356.05481	14.5835

Totals : 4.53350e4 2559.37830

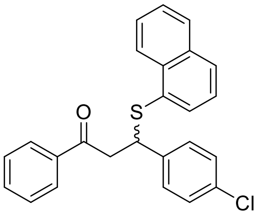

***(S)*-12j**

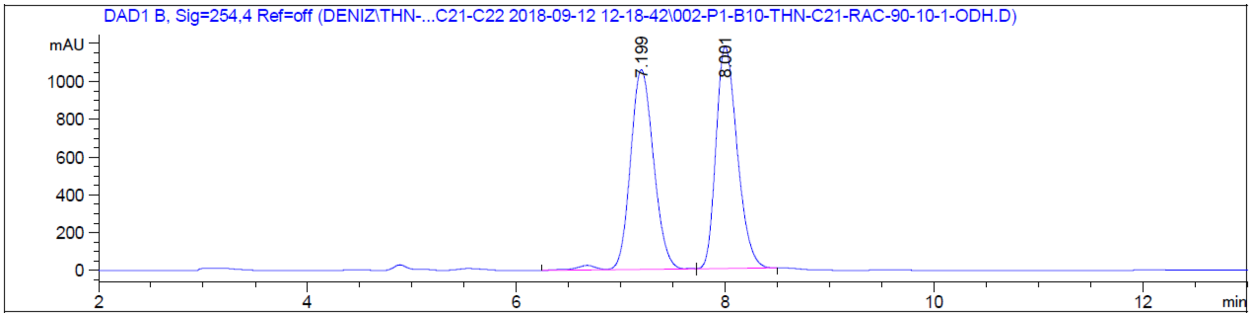

Signal 2: DAD1 B, Sig=254,4 Ref=off

Peak #	RetTime [min]	Type	Width [min]	Area [mAU*s]	Height [mAU]	Area %
1	7.199	VV R	0.2365	1.63263e4	1055.59644	49.5946
2	8.001	BB	0.2194	1.65933e4	1173.67212	50.4054

Totals : 3.29196e4 2229.26855

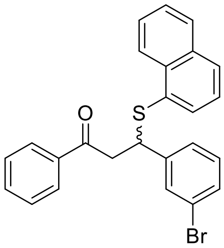

***(rac)*-12k**

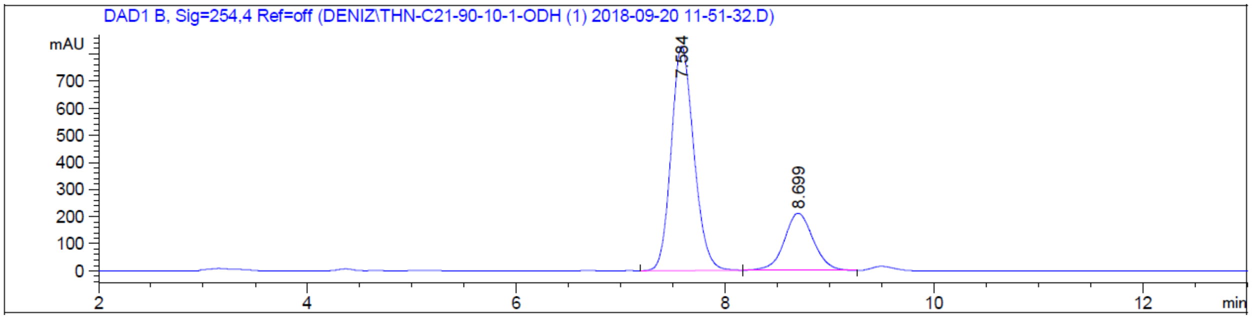

Signal 2: DAD1 B, Sig=254,4 Ref=off

Peak #	RetTime [min]	Type	Width [min]	Area [mAU*s]	Height [mAU]	Area %
1	7.584	BB	0.2336	1.24848e4	827.05249	75.4838
2	8.699	BV	0.2968	4054.90894	210.45692	24.5162

Totals : 1.65397e4 1037.50941

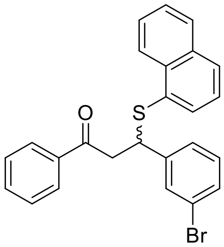

***(S)*-12k**

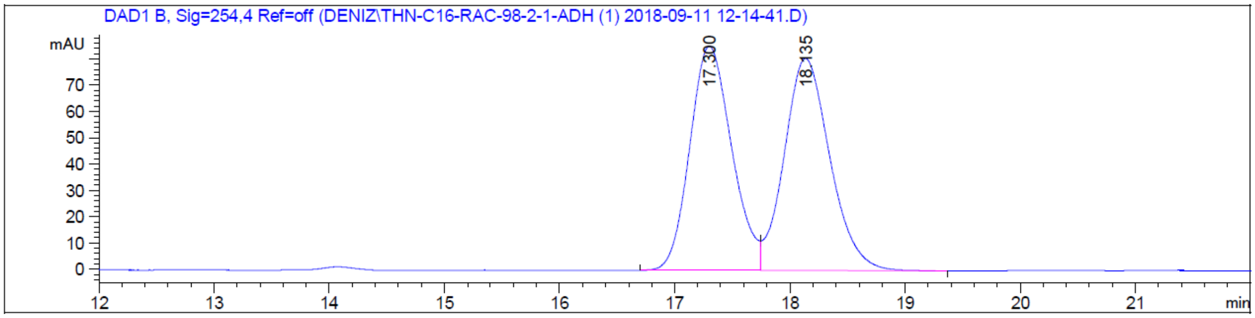

Signal 2: DAD1 B, Sig=254,4 Ref=off

Peak #	RetTime [min]	Type	Width [min]	Area [mAU*s]	Height [mAU]	Area %
1	17.300	BV	0.3896	2160.08887	85.40229	49.1325
2	18.135	VB	0.4128	2236.36548	80.82732	50.8675

Totals : 4396.45435 166.22961

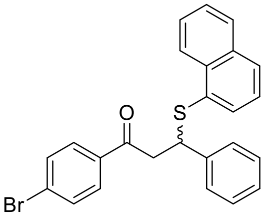

***(rac)*-12l**

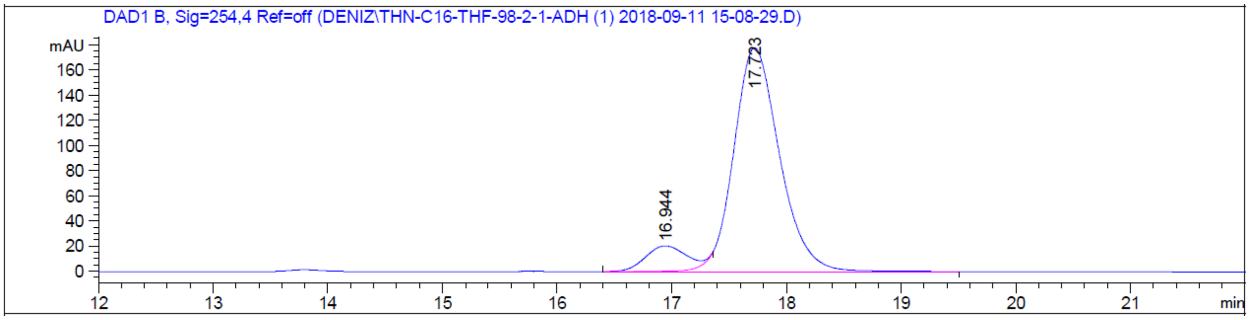

Signal 2: DAD1 B, Sig=254,4 Ref=off

Peak #	RetTime [min]	Type	Width [min]	Area [mAU*s]	Height [mAU]	Area %
1	16.944	BV E	0.3540	498.38351	20.36371	9.1561
2	17.723	VB R	0.4235	4944.82568	178.06689	90.8439

Totals : 5443.20920 198.43061

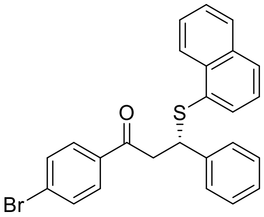

***(S)*-12l**

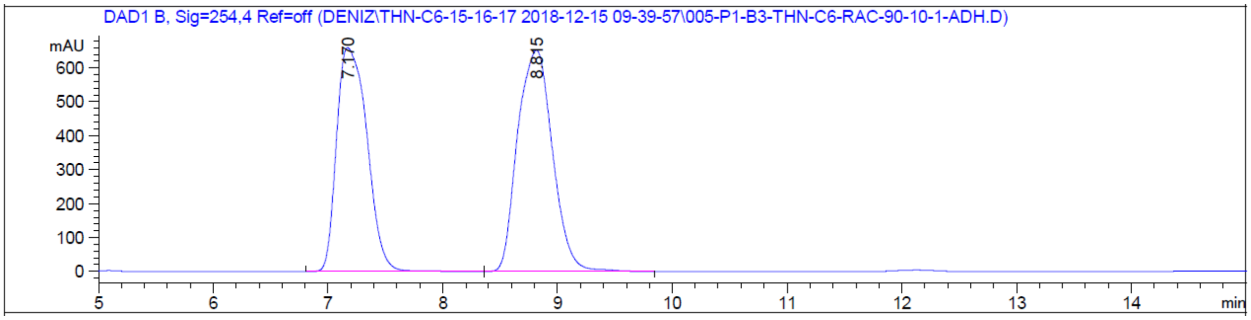

Signal 2: DAD1 B, Sig=254,4 Ref=off

Peak #	RetTime [min]	Type	Width [min]	Area [mAU*s]	Height [mAU]	Area %
1	7.170	BB	0.2999	1.21288e4	659.98712	46.8644
2	8.815	BB	0.3409	1.37519e4	652.39496	53.1356

Totals : 2.58807e4 1312.38208

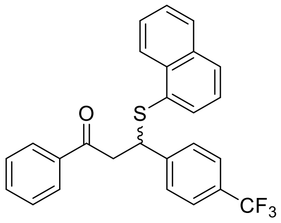

***(rac)*-12m**

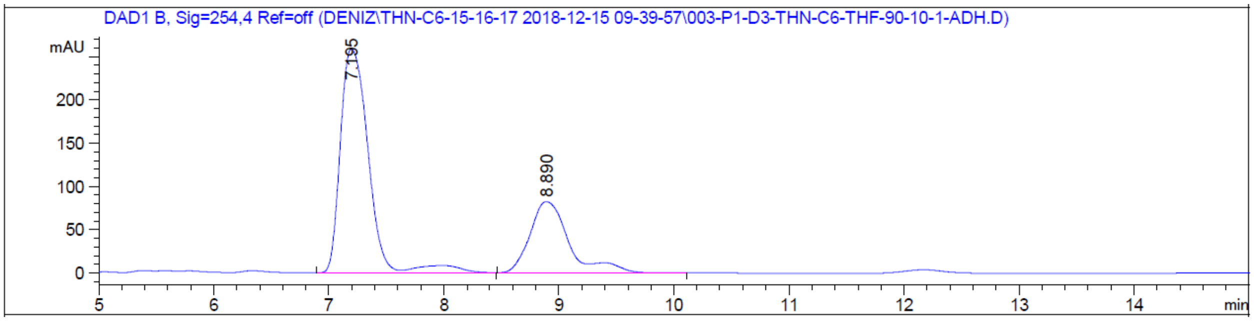

Signal 2: DAD1 B, Sig=254,4 Ref=off

Peak #	RetTime [min]	Type	Width [min]	Area [mAU*s]	Height [mAU]	Area %
1	7.195	BV R	0.2627	4425.58887	258.93863	69.4832
2	8.890	BV R	0.3337	1943.70544	82.17338	30.5168

Totals : 6369.29431 341.11201

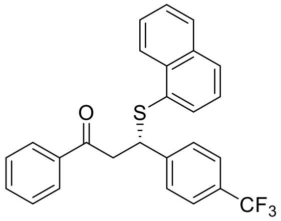

***(S)*-12m**

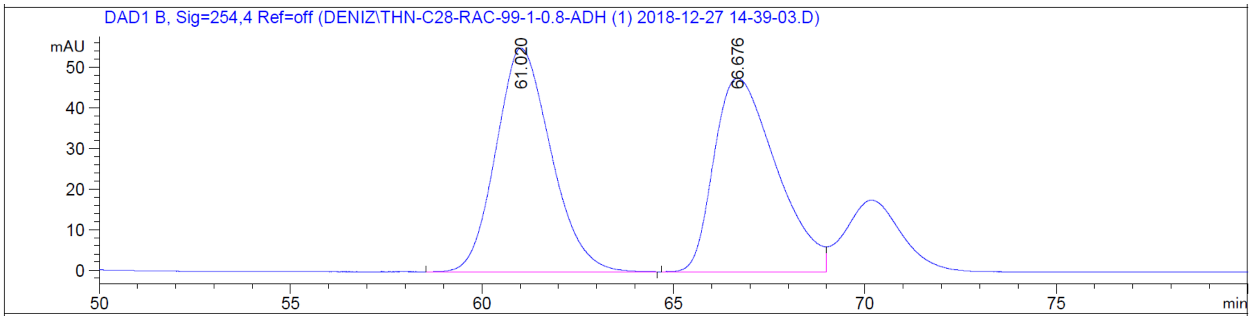

Signal 2: DAD1 B, Sig=254,4 Ref=off

Peak #	RetTime [min]	Type	Width [min]	Area [mAU*s]	Height [mAU]	Area %
1	61.020	BB	1.5805	5495.46240	55.05659	50.4427
2	66.676	BV	1.7547	5399.01172	47.62256	49.5573

Totals : 1.08945e4 102.67915

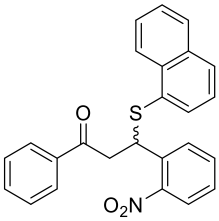

***(rac)*-12n**

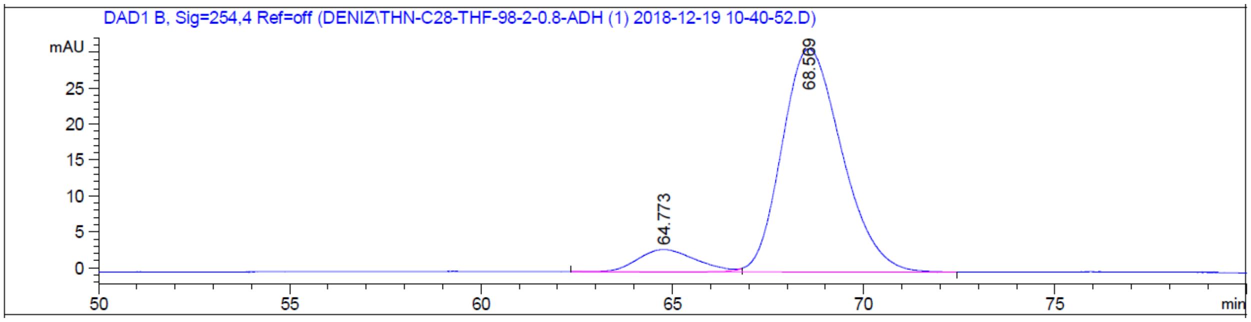

Signal 2: DAD1 B, Sig=254,4 Ref=off

Peak #	RetTime [min]	Type	Width [min]	Area [mAU*s]	Height [mAU]	Area %
1	64.773	BV E	1.6263	327.36319	3.11457	8.8062
2	68.569	VB R	1.7067	3390.04492	31.02762	91.1938

Totals : 3717.40811 34.14219

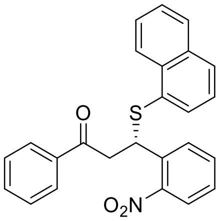

***(S)*-12n**

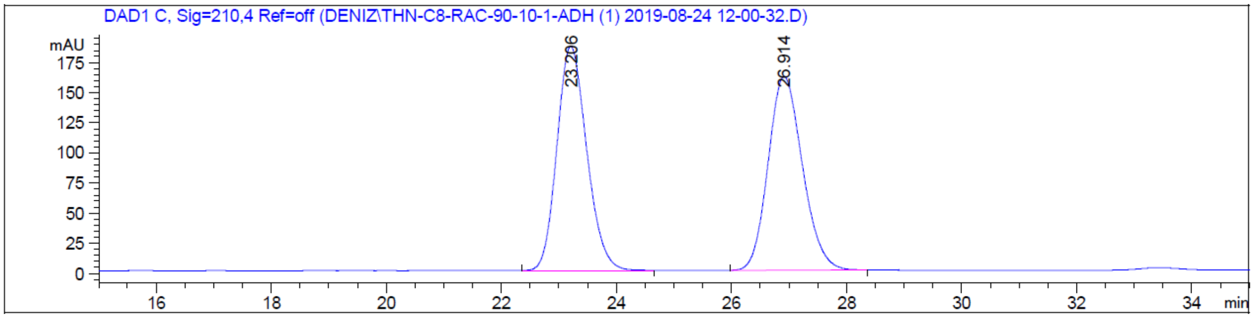

Signal 3: DAD1 C, Sig=210,4 Ref=off

Peak #	RetTime [min]	Type	Width [min]	Area [mAU*s]	Height [mAU]	Area %
1	23.206	BB	0.5442	6688.13721	185.93582	50.1028
2	26.914	BB	0.6263	6660.69531	160.79277	49.8972

Totals : 1.33488e4 346.72859

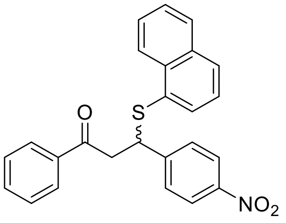

***(rac)*-12o**

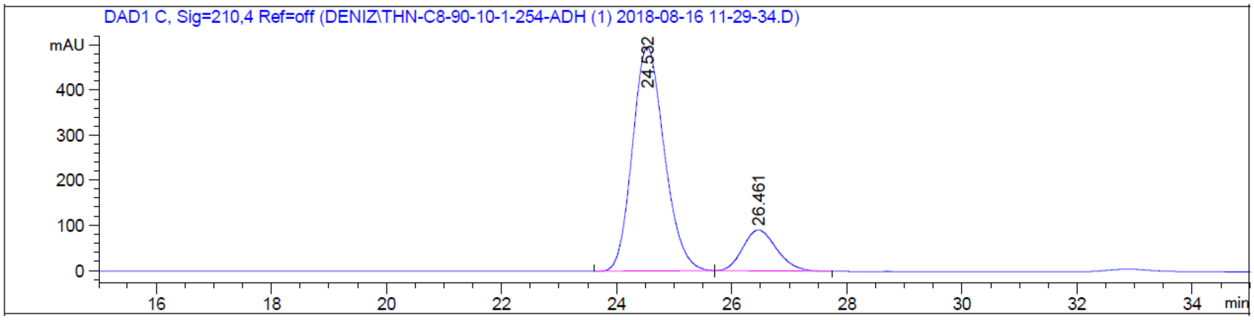

Signal 3: DAD1 C, Sig=210,4 Ref=off

Peak #	RetTime [min]	Type	Width [min]	Area [mAU*s]	Height [mAU]	Area %
1	24.532	BB	0.5899	1.91059e4	496.51318	83.7845
2	26.461	BB	0.5904	3697.71533	90.46827	16.2155

Totals : 2.28036e4 586.98145

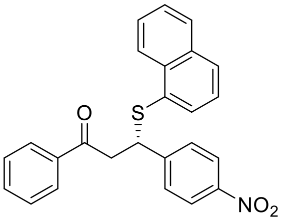

***(S)*-12o**

VI. ^{1}H and ^{13}C NMR spectra of sulfones

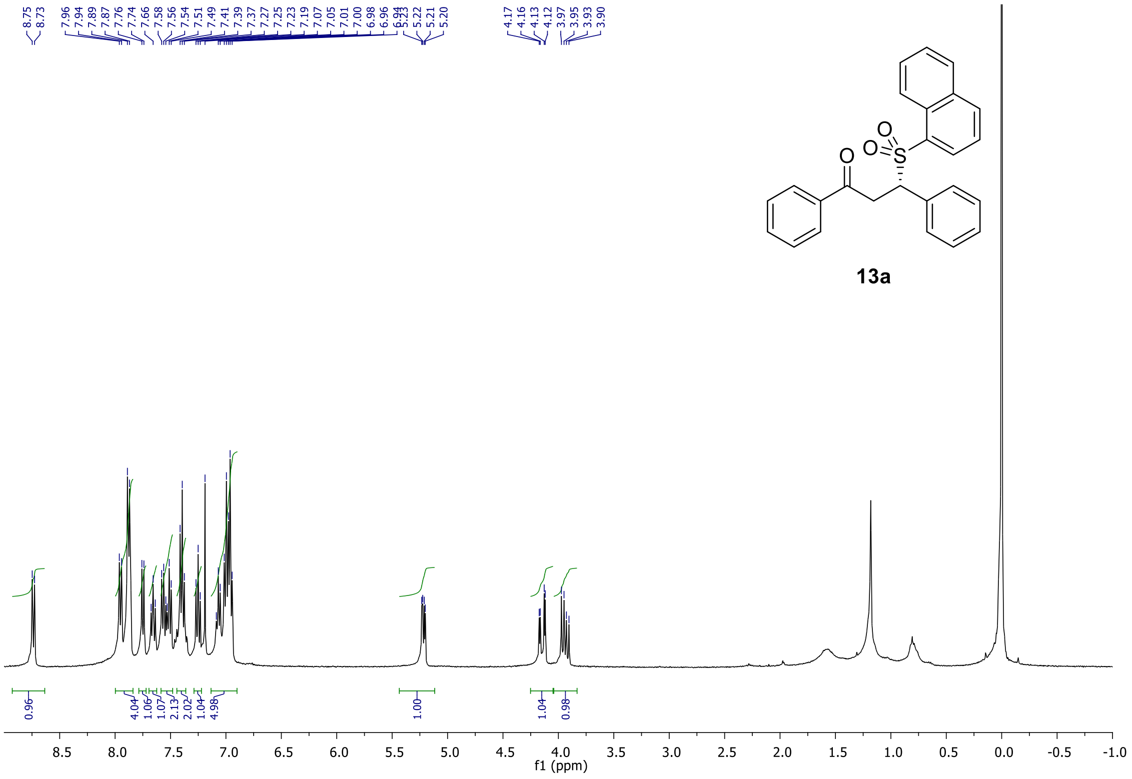

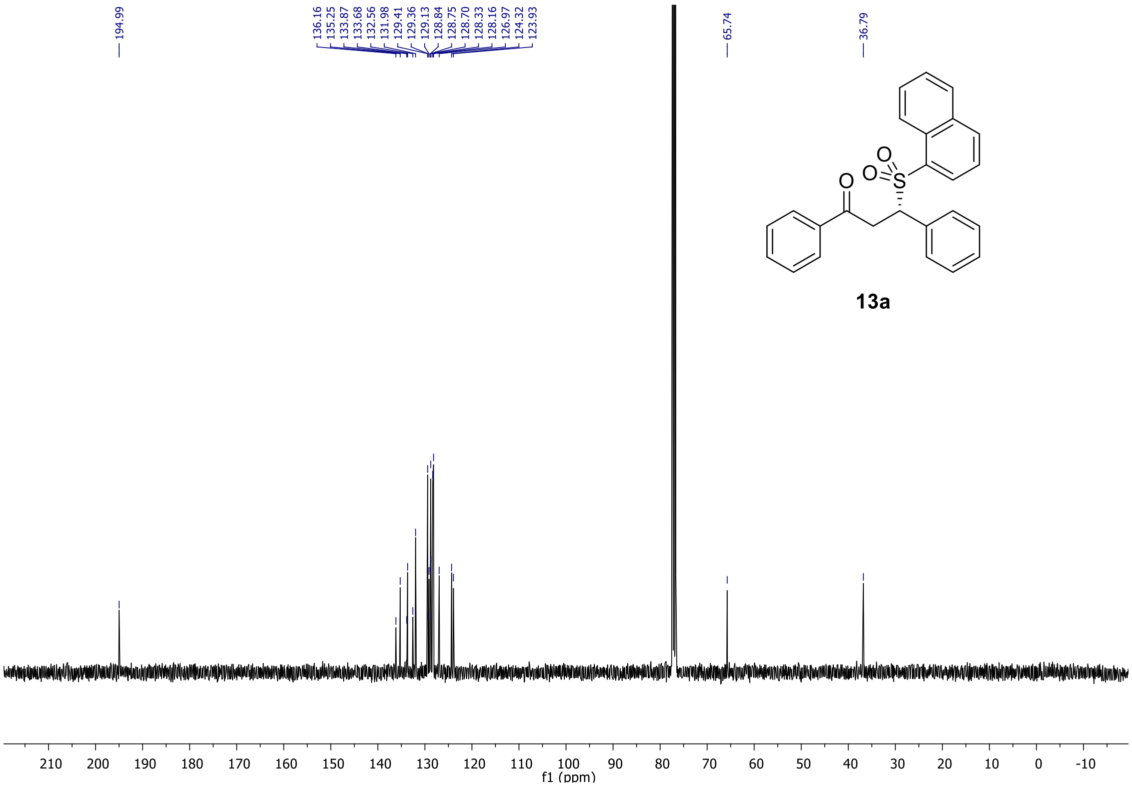

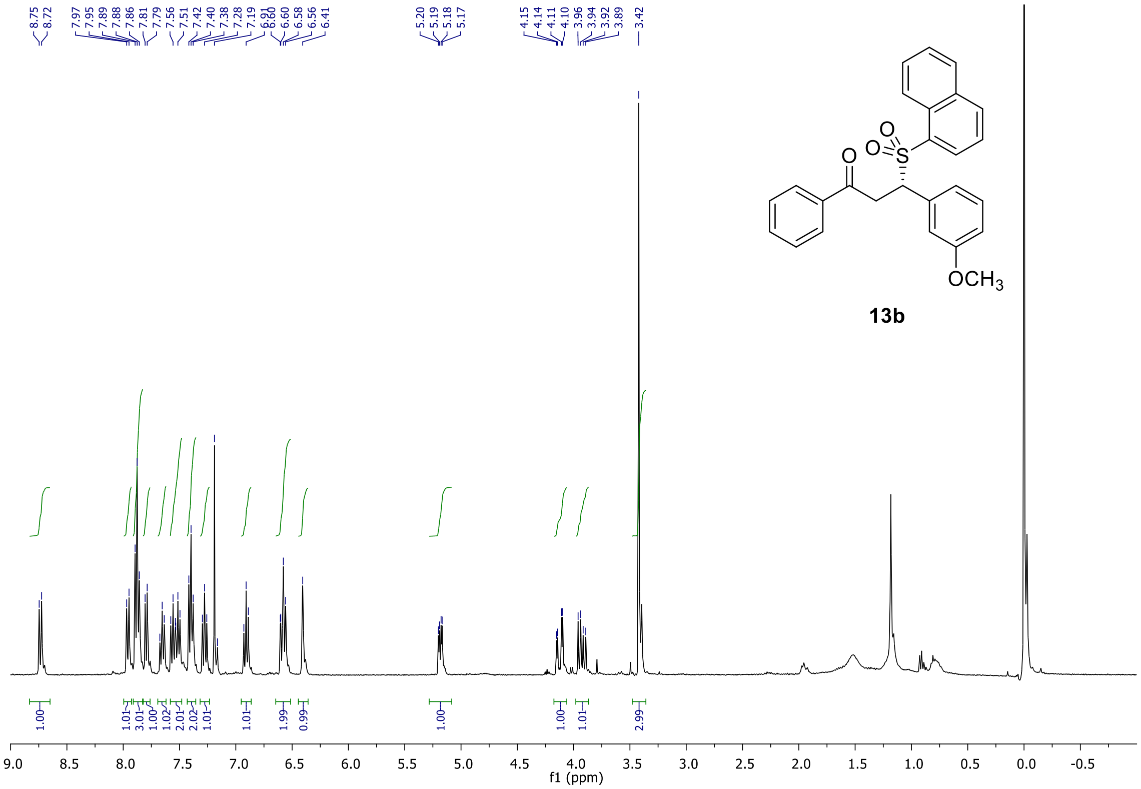

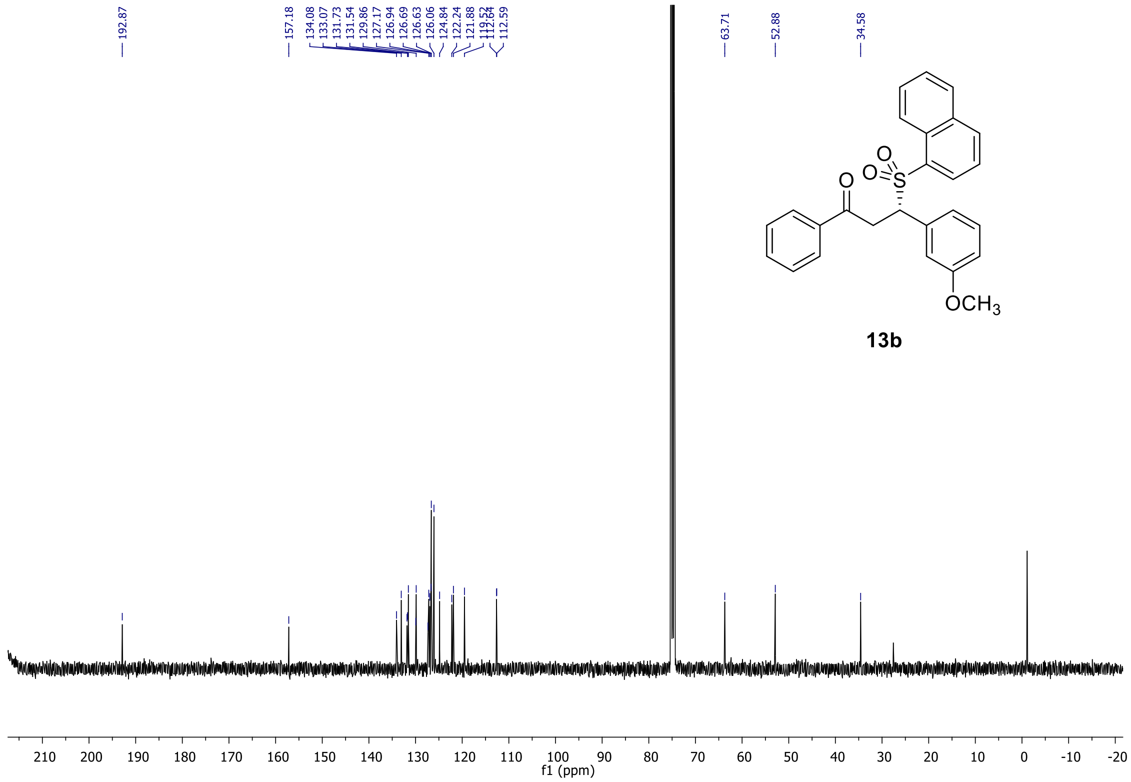

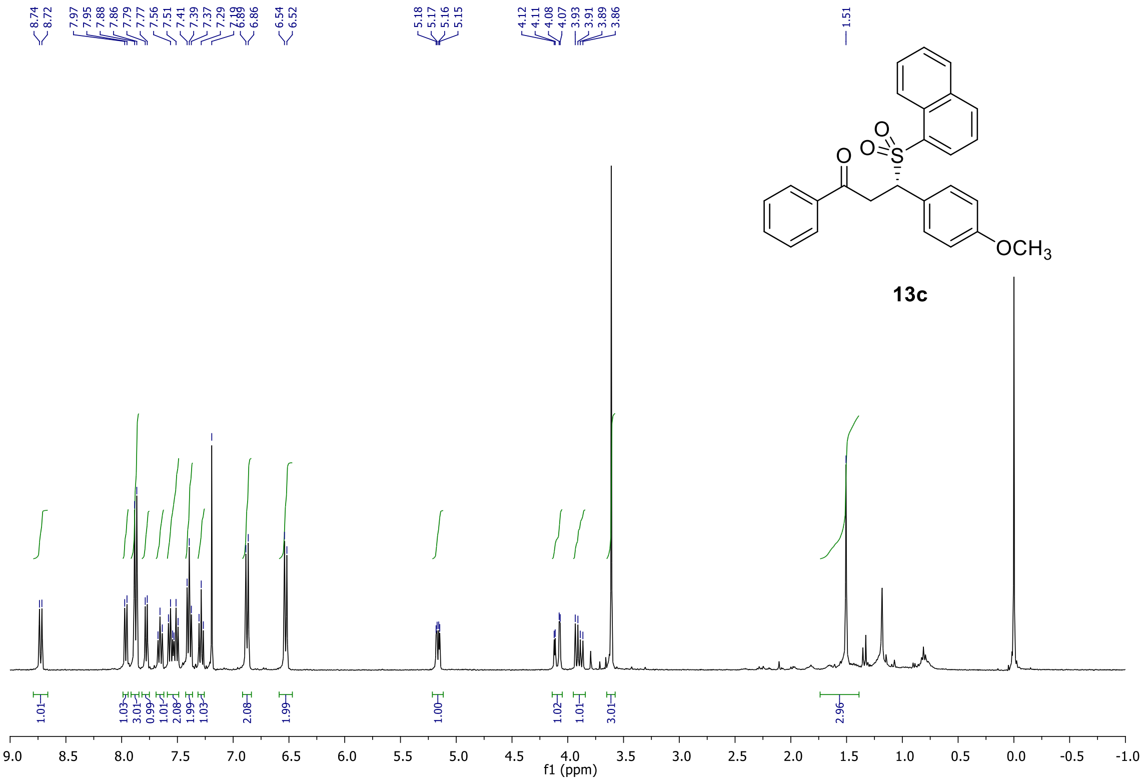
O
O
O
S
OCH3
13c
f1 (ppm)

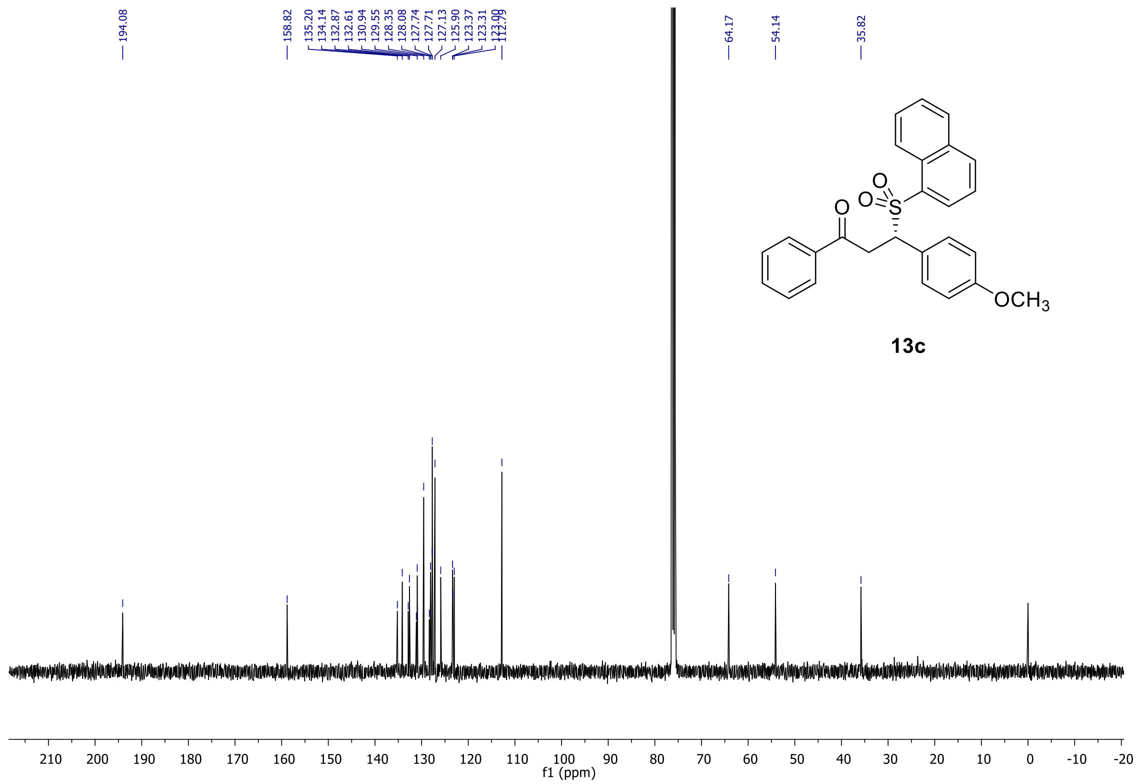
O
O
O
S
OCH3
13c
f1 (ppm)

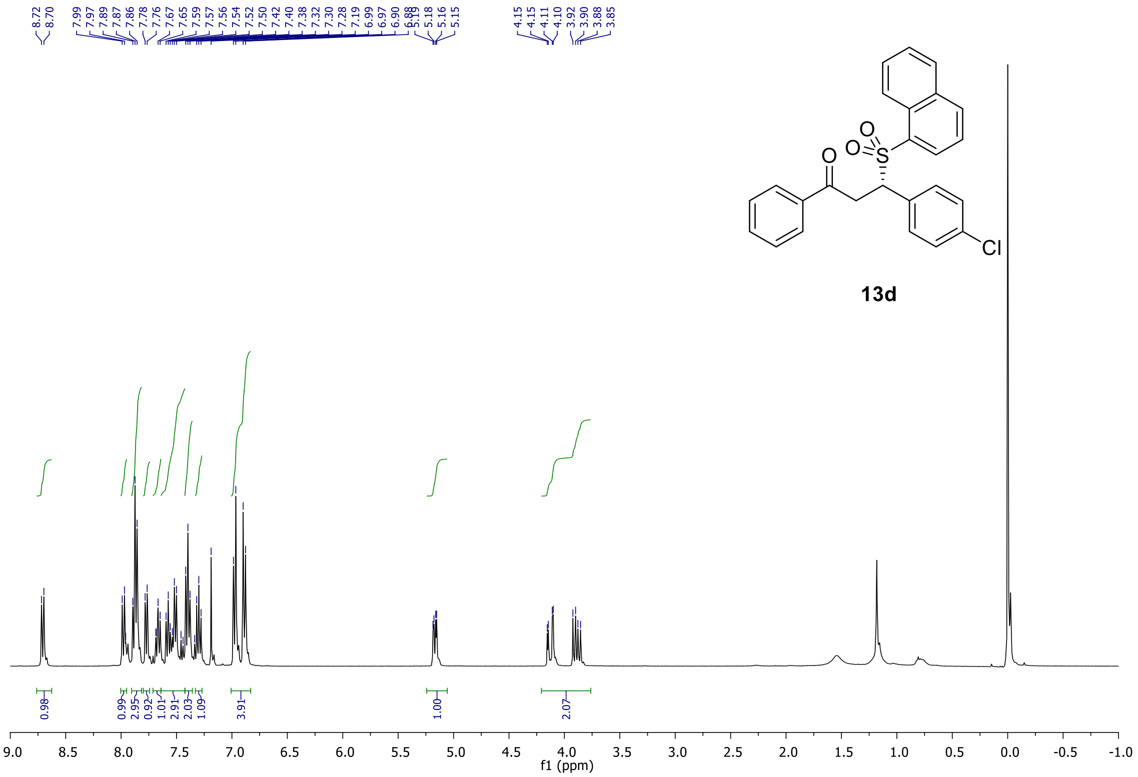

8.72
8.70
7.99
7.97
7.89
7.87
7.86
7.78
7.76
7.67
7.65
7.59
7.57
7.56
7.54
7.52
7.50
7.42
7.40
7.38
7.32
7.30
7.28
7.19
6.99
6.97
6.90
6.88
5.19
5.18
5.16
5.15
4.15
4.15
4.11
4.10
3.92
3.90
3.88
3.85
0.98
0.99
2.95
0.92
1.01
2.91
2.03
1.09
3.91
1.00
2.07
O
S
Cl
13d
f1 (ppm)

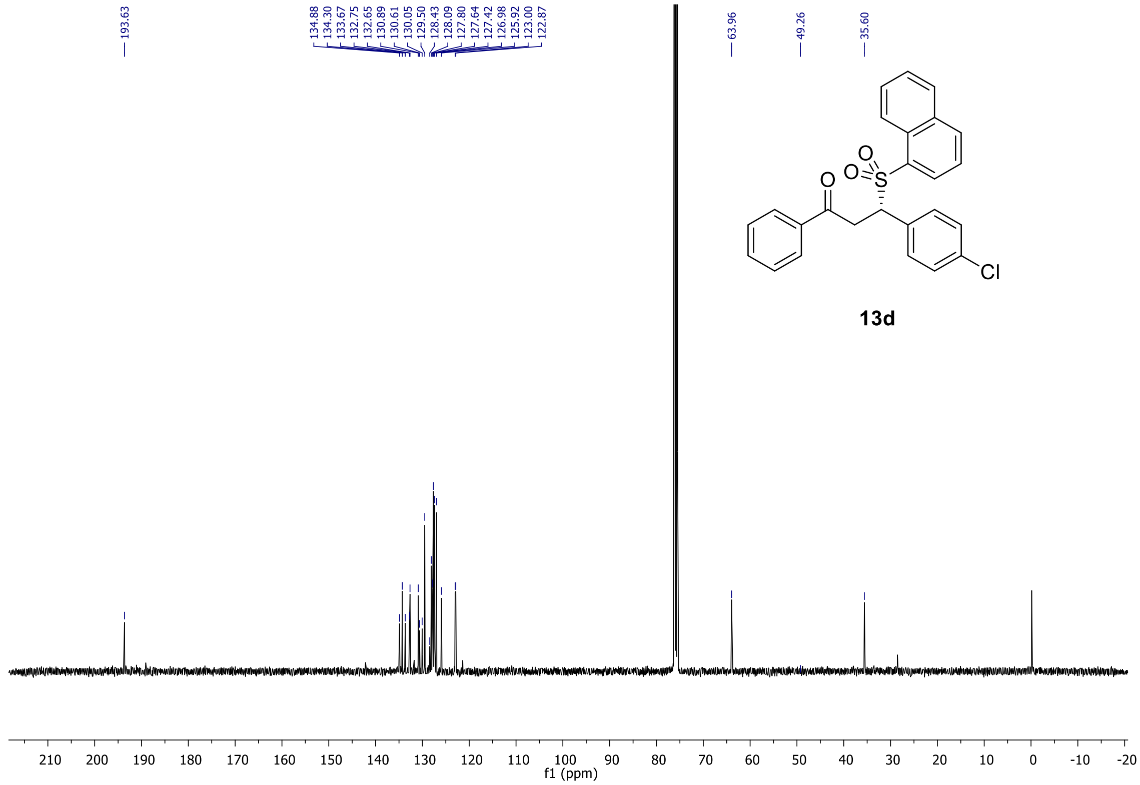

193.63
134.88
134.30
133.67
132.75
132.65
130.89
130.61
130.05
129.50
128.43
128.09
127.80
127.64
127.42
126.98
125.92
123.00
122.87
63.96
49.26
35.60
O
S
Cl
13d
f1 (ppm)

VII. HPLC chromatograms of sulfones

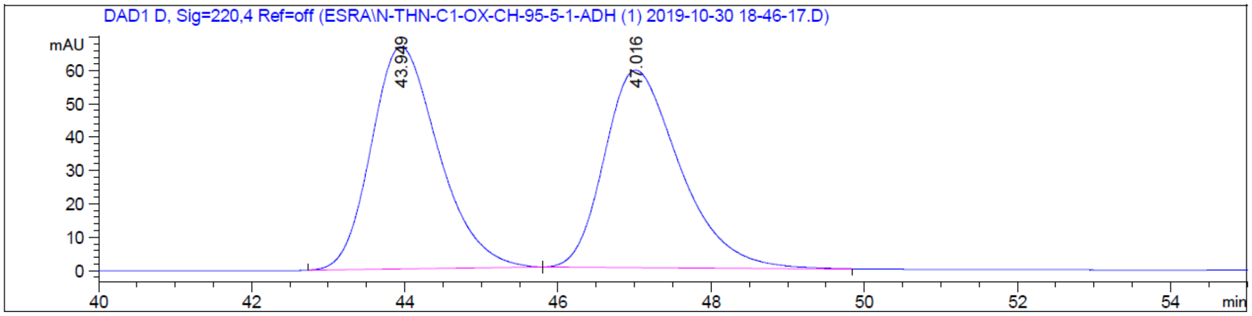

Signal 4: DAD1 D, Sig=220,4 Ref=off

Peak #	RetTime [min]	Type	Width [min]	Area [mAU*s]	Height [mAU]	Area %
1	43.949	BB	0.7672	4074.31372	66.64552	50.1447
2	47.016	BB	0.8190	4050.80054	59.22339	49.8553

Totals : 8125.11426 125.86890

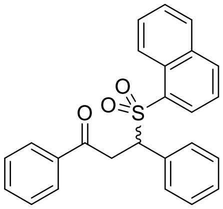

***(rac)*-13a**

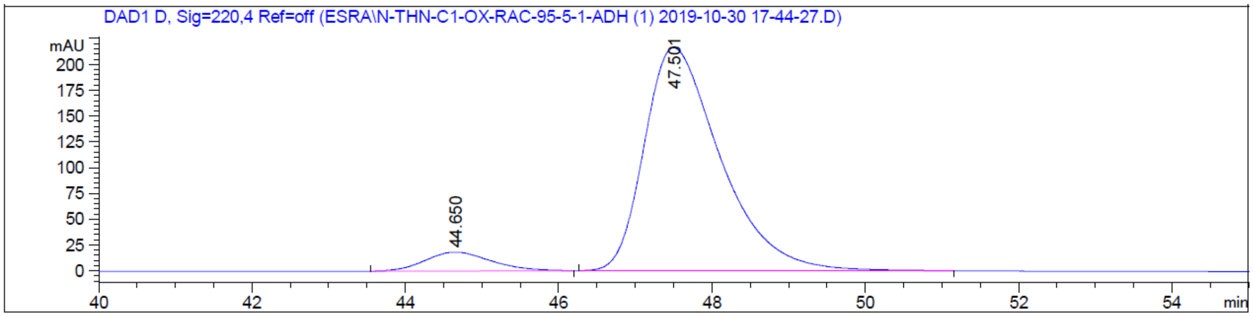

Signal 4: DAD1 D, Sig=220,4 Ref=off

Peak #	RetTime [min]	Type	Width [min]	Area [mAU*s]	Height [mAU]	Area %
1	44.650	BB	0.7026	1088.60718	18.16567	6.8077
2	47.501	BB	0.9581	1.49022e4	216.09126	93.1923

Totals : 1.59908e4 234.25693

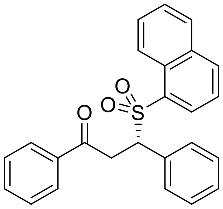

***(S)*-13a**

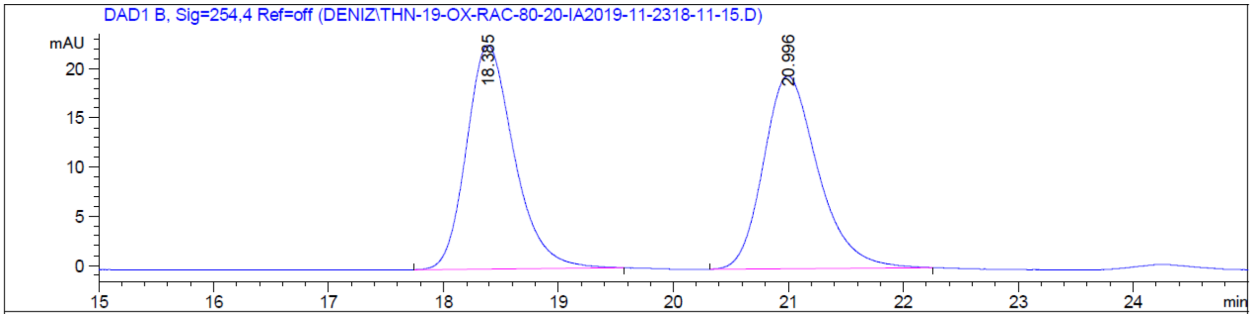

Signal 2: DAD1 B, Sig=254,4 Ref=off

Peak #	RetTime [min]	Type	Width [min]	Area [mAU*s]	Height [mAU]	Area %
1	18.385	BB	0.3980	654.52106	22.75792	50.3000
2	20.996	BB	0.4316	646.71472	19.61943	49.7000
Totals :				1301.23578	42.37735	

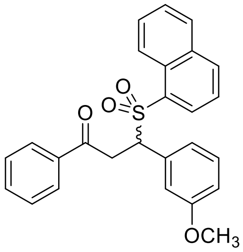

***(rac)*-13b**

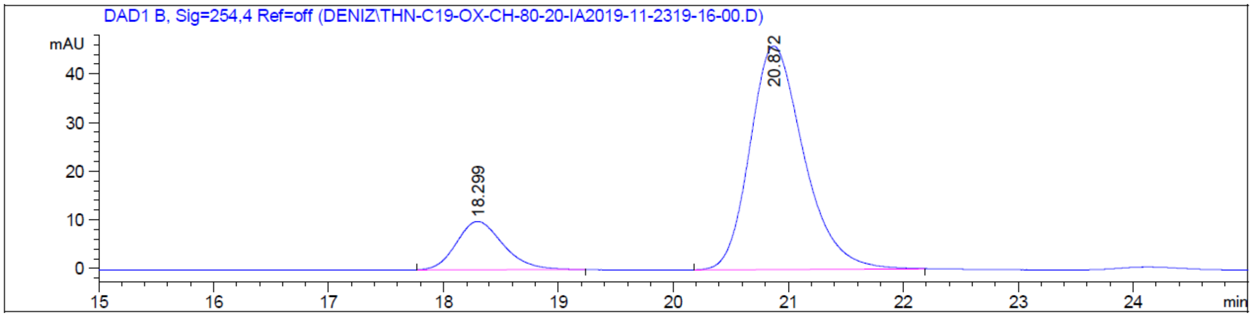

Signal 2: DAD1 B, Sig=254,4 Ref=off

Peak #	RetTime [min]	Type	Width [min]	Area [mAU*s]	Height [mAU]	Area %
1	18.299	BB	0.3332	278.74411	9.89344	15.7637
2	20.872	BB	0.4698	1489.52661	45.87250	84.2363
Totals :				1768.27072	55.76594	

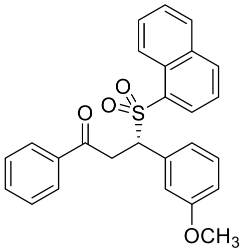

***(S)*-13b**

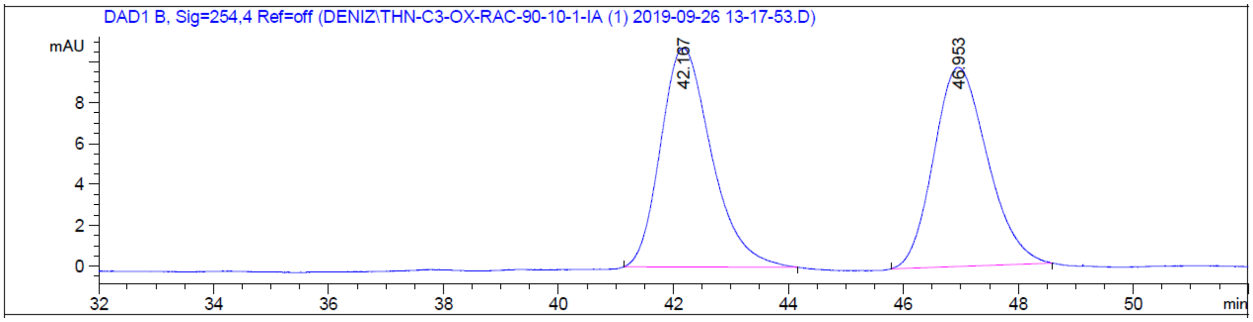

Signal 2: DAD1 B, Sig=254,4 Ref=off

Peak #	RetTime [min]	Type	Width [min]	Area [mAU*s]	Height [mAU]	Area %
1	42.167	BB	0.7203	659.29974	10.73143	50.9837
2	46.953	BB	0.7614	633.85889	9.75670	49.0163
Totals :				1293.15863	20.48813	

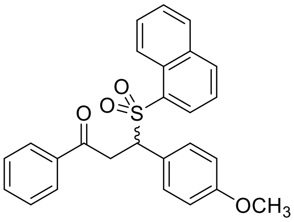

***(rac)*-13c**

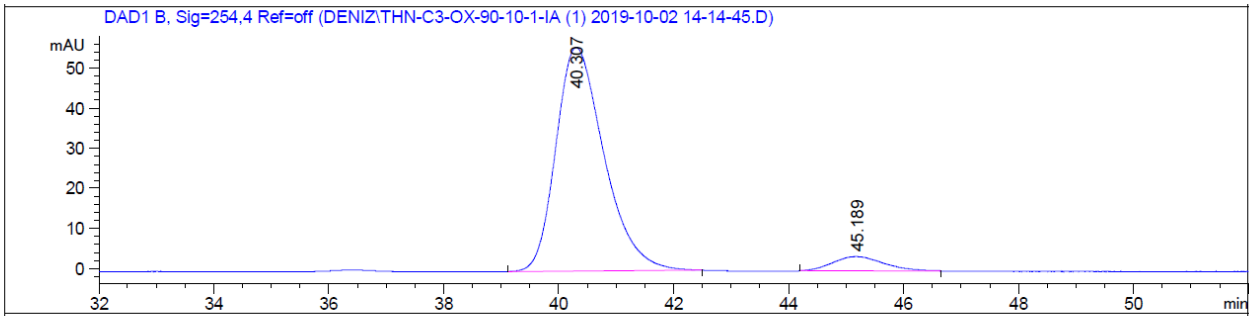

Signal 2: DAD1 B, Sig=254,4 Ref=off

Peak #	RetTime [min]	Type	Width [min]	Area [mAU*s]	Height [mAU]	Area %
1	40.307	BB	0.6901	3244.40845	55.78027	93.6986
2	45.189	BB	0.7186	218.19139	3.55215	6.3014
Totals :				3462.59984	59.33243	

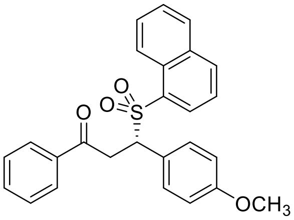

***(S)*-13c**

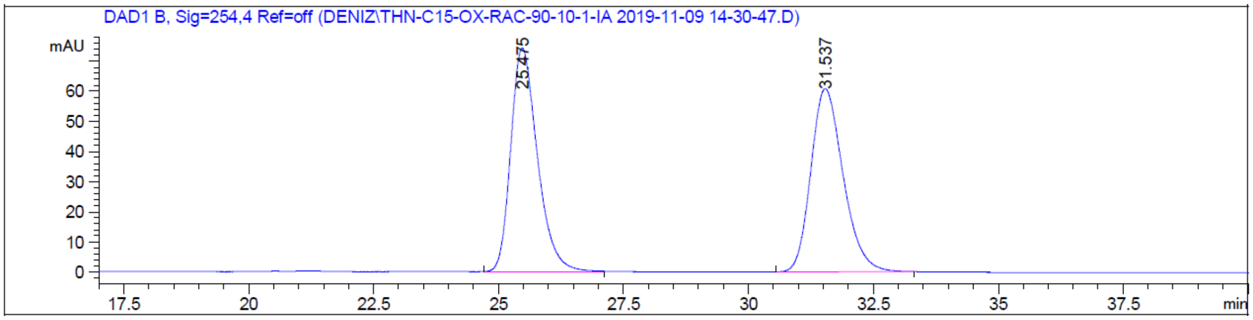

Signal 2: DAD1 B, Sig=254,4 Ref=off

Peak #	RetTime [min]	Type	Width [min]	Area [mAU*s]	Height [mAU]	Area %
1	25.475	BB	0.5347	2746.05493	74.20673	50.1017
2	31.537	BB	0.6004	2734.90576	60.78342	49.8983

Totals : 5480.96069 134.99014

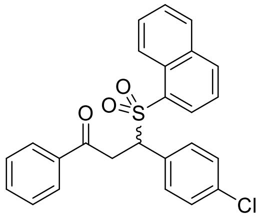

***(rac)*-13d**

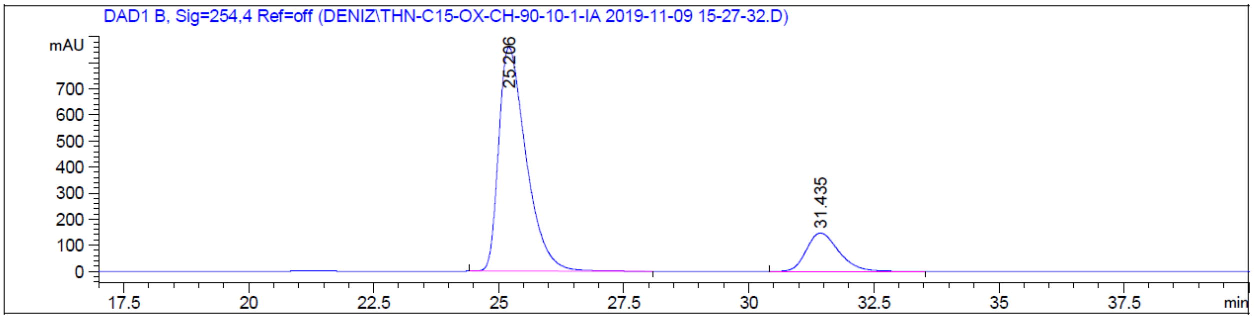

Signal 2: DAD1 B, Sig=254,4 Ref=off

Peak #	RetTime [min]	Type	Width [min]	Area [mAU*s]	Height [mAU]	Area %
1	25.206	BB	0.5778	3.27075e4	859.87500	83.1645
2	31.435	BB	0.6696	6621.16260	147.25027	16.8355

Totals : 3.93286e4 1007.12527

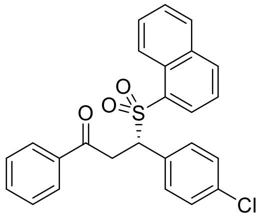

***(S)*-13d**
